# High-efficiency and stable deep-blue iridium phosphorescent OLEDs with enhanced charge transfer dynamics

**DOI:** 10.1038/s41377-026-02264-y

**Published:** 2026-06-02

**Authors:** Siqi Li, Kai-Ning Tong, Meng Zhang, Wei He, Chengcheng Wu, Junki Ochi, Di Wu, Kefei Shi, Xin Wang, Singyeong Jung, Feiyu Kang, Chihaya Adachi, Takuji Hatakeyama, Guodan Wei

**Affiliations:** 1 Institute of Materials Research, Tsinghua Shenzhen International Graduate School, Tsinghua University, Shenzhen, 518055 China; 2 Department of Chemistry, Graduate School of Science, Kyoto University, Kyoto, 606-8502 Japan; 3 Center for Organic Photonics and Electronic Research (OPERA), Kyushu University, Fukuoka, 819-0395 Japan

**Keywords:** Organic LEDs, Polymers

## Abstract

Deep-blue phosphorescent OLEDs (Ph-OLEDs) with high efficiency and stability are essential for advanced display technologies, ensuring sharp image quality and enhanced visibility. In this work, we report a novel class of asymmetric [3 + 2 + 1] coordinated iridium(III) complexes incorporate strongly electron-withdrawing trifluoromethyl (–CF_3_) and fluorine (–F) modified N-heterocyclic carbene ligands. This strategic molecular design enables efficient deep-blue emission. Among these complexes, the CF_3_-substituted Ir(III) complex (**CF**_**3**_**-2**) exhibits pronounced charge-transfer (CT) characteristics and a significantly enhanced radiative decay rate (${k}_{r}$ = 1.28 ×10⁶ s^-1^), enabling rapid and efficient phosphorescence at 443 nm. Devices employing **CF**_**3**_**-2** demonstrated exceptional maximum external quantum efficiency (*EQE*_max_) of up to 29.0%, with emission centered at 443 nm and Commission Internationale de L’Éclairage (CIE) coordinates of (0.147, 0.089), fulfilling National Television System Committee (NTSC) blue standards for high-quality displays. Meanwhile, devices employing **CF**_**3**_**-1** reached an *EQE*_max_ of 24.6% with a maximum luminance of 6542 cd m^−2^ and CIE_x,y_ of (0.152,0.126), demonstrating high color purity and efficiency. A control device fabricated without sensitization using **CF**_**3**_**-1** further confirms its intrinsic material stability by exhibiting a remarkable operational lifetime of LT_50_ of 3875 h at L = 100 cd m^−2^ with CIE_x,y_ of (0.132,0.131). Furthermore, hyper-OLEDs were developed using these complexes as phosphorescent sensitizers. The hyper-OLED incorporating **CF**_**3**_**-1** with the TADF emitter *v*-DABNA achieved an impressive device lifetime of LT_50_ = 2127 h at 100 cd m^−2^. In parallel, the **CF**_**3**_**-2**-sensitized hyper-OLED using DOB2-DABNA-A achieved a deep-blue emission with CIE coordinates of (0.146, 0.067) and a lifetime of LT_50_ = 373 h under the same luminance, representing a significant advancement in the practical stability of deep-blue OLEDs. Notably, we demonstrate the successful integration of these deep-blue Ph-OLEDs with OLED-on-TFT microdisplay technology, achieving a pixel resolution of 94 PPI (270 × 270 μm) with programmable emission patterns. This innovative molecular coordination design strategy provides valuable insights into ligand engineering and exciton management, opening new pathways toward high-efficiency, long-lifetime deep-blue OLEDs for next-generation microdisplay and display technologies.

## Abstract (web summary)

**CF**_**3**_**-2** exhibits enhanced transition dipole moments and radiative decay rate, enabling deep-blue OLEDs with 29.0% EQE, meeting National Television System Committee (NTSC) for deep-blue display application.

## Graphical abstract

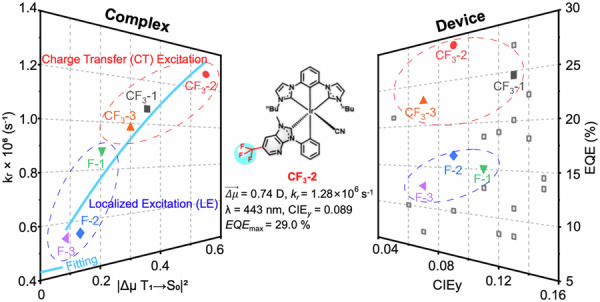

## Introduction

Phosphorescent complexes are crucial in OLED technology due to their ability to harvest both singlet and triplet excitons, theoretically achieving 100% quantum efficiency^1,2^. Among these, cyclometallated Ir(III) complexes stand out as highly promising candidates for display and lighting applications, with their high quantum efficiency, short excited state lifetimes, tunable emission colors, and good thermal stability^3–6^. However, despite significant progress in red and green phosphorescent materials, the development of efficient deep-blue light-emitting materials remains a critical challenge, limiting the practical application and advancement of OLED-based display technologies^7–9^. Thus, innovative molecular designs are urgently required to realize deep-blue phosphorescence with high quantum efficiency and long device lifetimes.

Traditionally, phosphorescent Ir(III) complexes employed symmetric [2 + 2 + 2] coordination mode, typically forming heteroleptic or homoleptic structures with bidentate ligands such as phenylpyridines or phenylimidazoles^6,10,11^. Representative deep-blue emitters such as FIrpic^12^ and mer-Ir(tfpi_tmBn)_3_^13^ have achieved notable Commission Internationale de L’Eclairage (CIE) coordinates of (0.16, 0.08), yet they suffer from relatively limited maximum external quantum efficiencies (*EQE*_max_ < 15%) and severe efficiency roll-off at practical luminance levels, impeding commercial application. To overcome these intrinsic limitations, our research group has pioneered the development of highly asymmetric [3 + 2 + 1] coordinate Ir(III) complexes specifically tailored for deep-blue emission through advanced ligand engineering strategies^14–20^. Through systematic optimization, our [3 + 2 + 1] coordinated Ir(III) complexes have achieved groundbreaking photophysical performance, including near-unity photoluminescence quantum yields (PLQYs ~100%), dramatically reduced excited-state lifetimes (~1 μs)^20^, and record-setting OLED device efficiencies (*EQE*_max_ of 30.7%) with exceptional deep-blue color purity (CIE_x,y_:0.140, 0.148)^21^. Nevertheless, further molecular engineering strategies are necessary to break existing performance bottlenecks, particularly to surpass efficiency roll-off and stability limitations.

Increasing ligand field strength through the incorporation of N-heterocyclic carbene (NHC) ligands presents an innovative pathway to address these challenges. NHC ligands are renowned for their exceptional electron-donating strength and steric bulk, significantly enhancing the ligand field to suppress undesirable non-radiative metal-centered (^3^MC) decay pathways and promoting deep-blue emission by widening highest occupied molecular orbital (HOMO)- the lowest unoccupied molecular orbital (LUMO) gaps^22–26^. For instance, Thompson and Forrest introduced Ir(III) complexes featuring NHC ligands emitting violet-blue luminescence at wavelengths around 400 nm, achieving notable EQE performances exceeding 10%^26,27^ Inspired by these breakthroughs, we designed a new family of [3 + 2 + 1]-coordinated Ir(III) complexes integrating imidazole-based NHC ligands paired with strongly electron-withdrawing trifluoromethyl (-CF_3_) and fluorine (-F) substituents. The electronic modulation from these substituents profoundly influences excited-state characteristics, distinctly favoring charge-transfer (CT) or locally excited (LE) states, thereby enabling precise emission tuning and enhanced device stability.

**Scheme 1:**
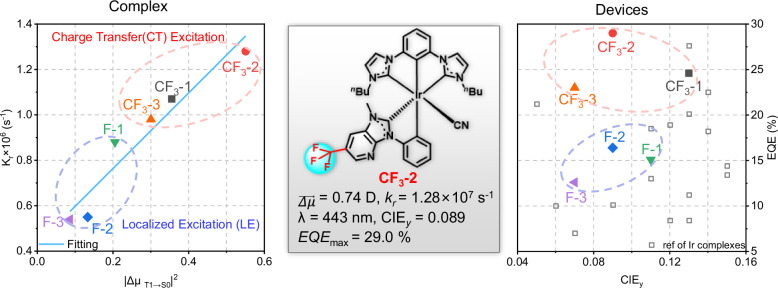
Phosphorescent Ir complexes exhibit a linear relationship between the radiative decay rate (K_r_) and the square of transition dipole moments (TDMs) from T_1_ to S_0_ (|$\vec{{\boldsymbol{\Delta }}{\boldsymbol{\mu }}}$_T1_${\boldsymbol{\to }}$_S0_|), with the **CF**_**3**_**-2** complex showing the highest values of both $\vec{{\boldsymbol{\Delta }}{\boldsymbol{\mu }}}$_T1_${\boldsymbol{\to }}$_S0_ and ${k}_{r}$. Owing to the outstanding performance of complex **CF**_**3**_**-2**, deep-blue OLEDs have achieved a record maximum external quantum efficiency (*EQE*_max_) of 29.0% and Commission Internationale de L’Eclairage (CIE) coordinates of (0.147, 0.089), meeting the deep-blue requirements specified by the National Television System Committee (NTSC) for display application

In this study, we report a comprehensive study of six newly synthesized NHC-based [3 + 2 + 1] Ir(III) complexes(**CF**_**3**_**-1,** **CF**_**3**_**-2,** **CF**_**3**_**-3,** **F-1,** **F-2**, and **F-3)**, demonstrating unprecedented deep-blue emission at wavelengths ranging from 416 to 456 nm. The complex **CF**_**3**_**-2**, particularly noteworthy for its high decay rate (${k}_{r}$ = 1.28 × 10⁶ s⁻¹), achieved an *EQE*_max_ of 29.0% and deep-blue CIE_x,y_ coordinates of (0.147, 0.089). Moreover, the hyper-OLED incorporating **CF**_**3**_**-1** as a phosphorescent sensitizer and *v*-DABNA as an emitter attained an excellent device lifetime of LT_50_ = 2127 h at L = 100 cd m^−2^ with CIE_x,y_ of (0.120, 0.120). Meanwhile, hyper-OLED based on **CF**_**3**_**-2** as a phosphorescent sensitizer and DOB2-DABNA-A as an emitter demonstrated deep-blue CIE_x,y_ of (0.146, 0.067), with device lifetime of LT_50_ = 373 h at L = 100 cd m^−2^, significantly advancing the practical deep-blue application. Finally, we demonstrate the successful integration of these emitters in thin-film transistor (TFT) micro-displays at a resolution of 94 PPI (270 × 270 μm) for programmable patterns, representing a substantial leap forward for next-generation deep-blue OLED technology (Scheme 1).

## Results

### Syntheses of the Ir(III) complexes

**Fig. 1:**
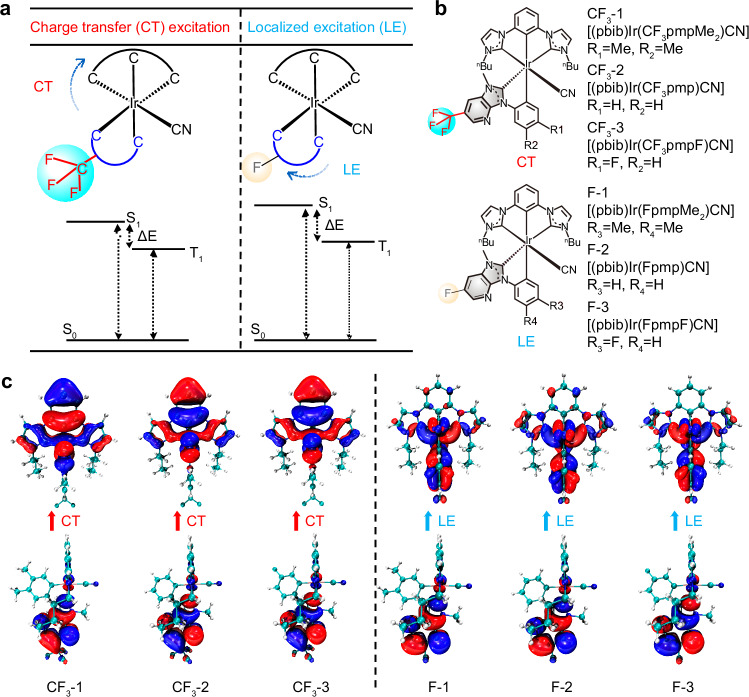
Molecular design and their triplet excited stated charge distribution of the Ir(III) complexes. **a** Schematic of the energy levels of complexes with charge transfer (CT) excitation and localized excitation (LE). The energy difference between the excited state (S_1_/T_1_) and ground state (S_0_) is lower in complexes with CT than those with LE. **b** chemical structure, **c** natural transition orbital (NTO) of different Ir(III) complexes

A series of six asymmetric [3 + 2 + 1] Ir(III) complexes (Fig. 1 and Supplementary Fig. S1) were synthesized featuring a tridentate NHC ligand (pbib) and a series of C^C-type bidentate ligands modified with CF_3_ or F substituents^18,20^. The bidentate ligands—CF_3_pmpMe_2_, CF_3_pmp, CF_3_pmpF, FpmpMe_2_, Fpmp, and FpmpF—were prepared via condensation and cyclization of substituted anilines and nitropyridines, followed by methylation and coordination with Ir(III). The final complexes [(pbib)Ir(C^C)CN], labeled as **CF**_**3**_**-1/2/3** and **F-1/2/3**, were obtained via a two-step process involving initial cyclometalation and subsequent cyanation. The electronic nature of the substituents strongly influenced the excited-state characteristics: CF_3_ groups promoted charge-transfer (CT) character, while F substituents favored locally excited (LE) states (Fig. 1a, b). The energy difference between the excited state (S_1_/T_1_) and ground state (S_0_) is lower in complexes with CT than those with LE. Surprisingly, the introduction of CF_3_/F to carbene alters the excited state of the Ir(III) complexes, suggesting a charge-transfer (CT) excitation for complexes with CF_3_ and a locally excited (LE) state for complexes with F, as confirmed by natural transition orbital (NTO) calculations (Fig. 1c). These findings are crucial for enhancing OLED performance by modulating the excited state, which in turn affects energy transfer within the emitting layer.

All complexes were fully characterized by ^1^H (400 MHz in CDCl_3_) and ^19^F (376 MHz in CDCl_3_) nuclear magnetic resonance (NMR) spectroscopy (Supplementary Figs. S2–50), high-resolution mass spectroscopy (Supplementary Figs. S51–56) and thermogravimetry analysis (TGA) (Supplementary Fig. S57 and Supplementary Table S1), confirming their structures and high thermal stability.

### Crystallography

Single crystals of the precursor complex [(pbib)Ir(Fpmp)I] and target complex of **F-2** and **CF**_**3**_**-3** were successfully obtained by slow vapor diffusion of chloroform into hexyl hydride solutions (Supplementary Table S2). The X-ray crystal structures (Supplementary Fig. S58) confirms distorted octahedral geometry, with the rigid tridentate NHC ligand (pbib) providing structural stability^14^. In [(pbib)Ir(Fpmp)I], the Ir-C(NHC) bonds are shorter than Ir-C (benzene) (2.002–2.065 Å), indicating the stronger electron-withdrawing nature of the NHC ligand. This trend is consistent in the **F-2** and **CF**_**3**_**-3**, as shown in Supplementary Fig. S58B, C. The C-Ir-C bite angle of bidentate ligand in **[(pbib)Ir(Fpmp)I]** is 78.4°, decreasing slightly to 77.9° in **F-2** due to the withdrawing -CN group. For **CF**_**3**_**-3**, the Ir-C bonds of bidentate ligand (2.051 Å and 2.091 Å) are slightly elongated compared to **F-2** (2.065 Å and 2.083) Å, attributed to the electron-withdrawing fluorine and trifluoromethyl groups. These subtle variations in bond metrics support the electronic influence of ligand substituents on complex geometry.

To clarify the impact of substituents on intermolecular interactions, we analyzed the π-π stacking distances and spatial configurations of **CF**_**3**_**-3** and **F-2** using their X-ray single-crystal data (Supplementary Fig. S59). For **F-2** (LE-state dominant), the centroid distance between adjacent tridentate ligand is only 9.035 Å. This closer proximity enables partial π-π overlap, promoting intermolecular aggregation. Also, the bidentate ligands of **F-2** exhibit even closer stacking with distance of 8.478 Å, indicating strong π-π interaction. For **CF**_**3**_**-3** (CT-state dominant), the sterically bulky -CF_3_ group on the bidentate ligand could weaken intermolecular π-π stacking with centroid distance of 11.637 Å. This weak intermolecular stacking minimizes exciton migration between adjacent **CF**_**3**_**-3** molecules, reducing triplet-triplet annihilation (TTA) and concentration quenching.

### Photophysical properties

**Fig. 2:**
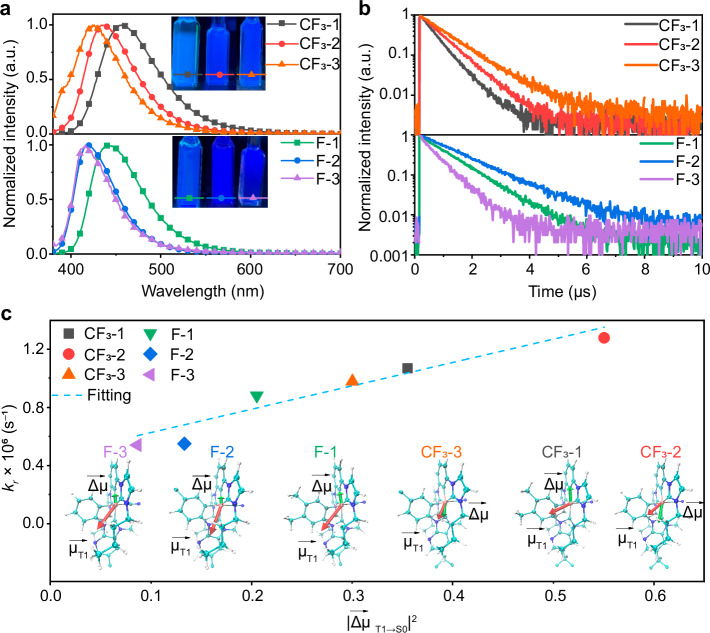
Photophysical properties of **CF3-1**, **CF3-2**, **CF3-3**, **F-1**, **F-2** and **F-3** Iridium complexes. **a** Emission spectra; **b** lifetime decay profiles of all complexes in toluene solution at 298 K; **c** Phosphorescent Ir complexes exhibit a linear relationship between the square of transition dipole moments (TDMs) from T_1_ to S_0_ (|$\vec{{\boldsymbol{\Delta }}{\boldsymbol{\mu }}}$_(T1_${\boldsymbol{\to }}$_S0)_|) and radiative decay rate (k_*r*_), (Listed below are each structure with the permanent dipole moments ($\vec{{\boldsymbol{\mu }}}$_T1_) red arrows) and the transition dipole moments from T_1_ to S_0_ ($\vec{{\boldsymbol{\Delta }}{\boldsymbol{\mu }}}$, green arrows) of six Ir(III) complexes

The absorption and emission spectra of these asymmetric Ir(III) complexes are depicted in Fig. 2a, Supplementary Figs. S60, 61 and Table 1. All complexes exhibit ligand-centered (LC) π-π* at 302–305 nm and strong ligand-to-ligand charge transfer (^1^LLCT) transition at 313–315 nm. Additionally, absorption peaks of moderate intensity are evident within the wavelength range of 329–350 nm, primarily attributed to spin-allowed singlet metal-ligand charge transfer (^1^MLCT) and intra-ligand charge transfer (^1^ILCT) transitions. Specifically, compared to the other complexes, **CF**_**3**_**-2** and **CF**_**3**_**-3** display broader absorption peaks around λ = 373–374 nm and higher extinction coefficients around λ = 313–314 nm, arising from the spin-forbidden triplet metal-ligand charge transfer (^3^MLCT), implying a greater contribution of CT excitations than that of LE, in accordance with the calculation results shown in Fig. 1.

**Table 1:** Photophysical properties of studied [3 + 2 + 1] coordinated Ir(III) complexes

Complex	Medium (T [K])	Absorption	Emission				
		λ_max_^a^ (nm)	λ_max_^a^ (nm)	Φ^a^ (%)	τ^a^ (μs)	K_rb_ (10^6 ^s^−1^)	K_nrb_ (10^6 ^s^−1^)
**CF**_**3**_**-1**	Toluene (298 K)	303, 313, 330	456	80.0	0.51	1.57	0.39
	Toluene (77 K)	–	427	–	1.48		
	12.5 wt% in CzSi	–	459	83.2	0.78	1.07	0.22
**CF**_**3**_**-2**	Toluene (298 K)	303, 314, 331, 374	434	80.5	0.68	1.19	0.29
	Toluene (77 K)	–	408	–	6.55		
	12.5 wt% in CzSi	–	443	83.8	0.66	1.28	0.25
**CF**_**3**_**-3**	Toluene (298 K)	303, 313, 329, 373	425	67.0	0.91	0.74	0.36
	Toluene (77 K)	–	405	–	10.71		
	12.5 wt% in CzSi	–	432	73.6	0.75	0.98	0.35
**F-1**	Toluene (298 K)	305, 315, 339, 350	442	78.0	0.98	0.80	0.22
	Toluene (77 K)	–	412	–	2.60		
	12.5 wt% in CzSi	–	453	76.6	0.87	0.88	0.15
**F-2**	Toluene (298 K)	304, 315, 329	419	62.4	1.32	0.47	0.28
	Toluene (77 K)	–	402.422	–	10.19		
	12.5 wt% in CzSi	–	434	61.3	1.11	0.55	0.35
**F-3**	Toluene (298 K)	302, 314, 346	416	55.6	1.15	0.48	0.39
	Toluene (77 K)	–	399,422	–	10.64		
	12.5 wt% in CzSi	–	426	51.0	0.94	0.54	0.52

^a^Recorded at a concentration of 2 ⨯ 10^−5^ M in solution

The photoluminescence (PL) spectrum of all complexes in toluene at 298 K exhibits main emission peaks that are distributed within the wavelength range of 416–456 nm, representing a broad deep blue emission, as shown in Fig. 2a. Additionally, complexes featuring various bidentate ligands showcase relative red shifts. The presence of -CF_3_, an strong electron-withdrawing group, influences and modifies the distribution of the electron cloud in the complex, thereby causing a blue shift in the emission peak of the complex. Similarly, the methyl group possesses electron-donating properties, which mildly impacts the emission spectrum of the complex, resulting in a red shift in the emission peak for **CF**_**3**_**-1** complex. Additionally, when the substituents change from trifluoromethyl to fluorine, the emission spectrum of the Ir(III) complexes is significantly blue-shifted. To investigate their PL behavior at 77 K, the emission spectra of all the complexes display a vibronic structure with a 17–30 nm blue shift and narrowed full-width at half maximum (FWHM), as shown in Supplementary Fig. S59b. Notably, for complexes bearing LE excited states, changes in peak shape are observed more significantly at room and low temperatures. **F-2** and **F-3** exhibit shoulder peaks with a maximum at 422 nm. Additionally, decreasing the temperature to 77 K resulted in the following lifetimes of all Ir(III) complexes: 1.48 μs (0.51 μs at 298 K), 6.55 μs (0.68 μs), 10.71 μs (0.91 μs), 2.60 μs (0.98 μs), 10.19 μs (1.32 μs), and 10.64 μs (1.15 μs), respectively. This reduces the rate of non-radiative transitions and leads to much longer lifetimes at 77 K.

Then, we investigated the emission spectra in various solvents at 298 K of Ir(III) complexes, as shown in Supplementary Figs. S61, 62 and Supplementary Table S3. We could observe a red shift in the emission wavelength as the polarity of the solvent decreased. The observed variation in emission wavelengths can be primarily attributed to the ligand-to-ligand charge transfer (LLCT) of the complexes, demonstrating the positive influence of solvent polarity on intra-ligand charge transfer. The charge characters shown in Supplementary Table S12 could link the LLCT contribution to the complexes’ substituent-dependent photophysical behaviors. All CF_3_-series complexes exhibit a high LLCT proportion (~61.8–63.7%). In contrast, F-series complexes show a negligible LLCT proportion (~13.7–15.9%). As described in the main text, **CF**_**3**_**-2**’s emission wavelength red-shifts with decreasing solvent polarity (DMF: 471 nm → THF: 453 nm → DCM: 457 nm → toluene: 437 nm). This behavior is driven by its high LLCT component. **F-3** exhibits minimal solvent dependence (DMF: 430 nm → toluene: 416 nm, a mere 14 nm blue shift), which correlates with its low LLCT proportion. The LLCT excited state is more sensitive to changes in the environment, such as the polarity of solvents and molecules in the solid state or at different temperatures, which will lead to a narrowing of the energy gap between the IL and LLCT states of the complex.

In addition, we investigated the emission spectra and lifetime of an Ir(III) complex doped film with a concentration of 12.5% in CzSi. The results of the tests are presented in Supplementary Fig. S60c. All the complexes displayed emission peaks in the deep-blue range, specifically from λ = 426–459 nm, which is consistent with the emission behavior observed in solution. However, there was a notable red shift in the emission spectra of the Ir(III) complexes within the doped thin films as compared to the toluene solution. The primary driver of the red shift is the interaction between the Ir(III) complexes (guests) and the CzSi host, which stabilizes the complexes’ excited states and reduces the emission energy. Another driver is conformational restriction in the solid state of Ir(III) complexes. The [3 + 2 + 1] coordinated Ir(III) complexes exhibit flexible rotation in solution. In the CzSi film, however, solid-state packing restricts this rotation, locking the complexes into a more planar conformation, indirectly contributing to the red shift. Furthermore, all the complexes exhibited transient PL features, indicating a short excited-state lifetime ranging from 0.66 μs to 1.11 μs. This difference in lifetime suggests that these complexes demonstrated reduced triplet-triplet annihilation (TTA) and singlet-triplet quenching (STQ) processes, leading to an accelerated radiative decay rate in the emission layer. As a result of these findings, all the complexes displayed high photoluminescence quantum yield (PLQY) values, with Φ_PL_ of 51.0–83.8%.

### Electrochemistry

The electrochemical properties of Ir(III) complexes have a direct impact on their photophysical properties, rendering them an important consideration in the design of subsequent device structures. Through voltammetry (CV) analysis, the obtained CV curve in Supplementary Fig. S62a provides insight into the corresponding energy levels and energy gap differences. The oxidation process arises from the Ir³⁺/Ir⁴⁺ redox couple at the metal center^28^. However, this effect is also affected by the electron-withdrawing substituents (-CF_3_/-F) on the bidentate C^C ligand. For CF_3_-series complexes: The strongly electron-withdrawing -CF_3_ group reduces the electron-donating ability of the bidentate carbene ligand. This lowers electron density on the Ir center, making Ir³⁺ harder to oxidize (higher oxidation potential: **CF**_**3**_**-1**, +1.01 V; **CF**_**3**_**-2**, +1.11 V; **CF**_**3**_**-3**, +1.18 V). For F-series complexes, the Ir center has higher electron density, leading to lower oxidation potentials (**F-1**, +0.89 V; **F-2**, +1.02 V; **F-3**, +1.10 V). The complexes **CF**_**3**_**-1** and **F-1** exhibit reversible oxidation potentials in the range of +1.15 V to +1.30 V, predominantly attributed to the oxidation of bidentate ligands featuring nitrogen-hybridization carbene groups attached to the dimethylbenzene ring. Conversely, the other complexes demonstrate irreversible redox peaks, potentially due to the increased steric hindrance resulting from the larger volume of the benzene ring^18^. During the multiscan cyclic voltammetry experiments as shown in Supplementary Fig. S62B, we observed a dramatic decrease in the oxidation peak current of **CF**_**3**_**-1**, while **F-1** exhibited a more moderate decrease over 10 repeated cycles. The multiscan cyclic voltammograms revealed that the Ir(III) complexes bearing -CF_3_ substituent exhibit greater electrochemical stability than those with -F. This enhanced stability is due to the synergistic effects of electron withdrawal and induction caused by CF_3_^29^. Consequently, the higher operational stability of the **CF**_**3**_**-1**-based device can be attributed to the superior electrochemical stability compared to that of **F-1**.

The HOMO energy levels of the complexes were determined using oxidation potential data. The energy gap difference (E_gap_) was then calculated through the ultraviolet-visible (UV-Vis) absorption spectrum, thereby obtaining the corresponding LUMO energy levels. The relevant data are listed in Table 2. The estimated HOMO energy levels for the complexes were as follows: −5.38 eV, −5.49 eV, −5.57 eV, −5.28 eV, −5.43 eV, and −5.50 eV for **CF**_**3**_**-1,** **CF**_**3**_**-2,** **CF**_**3**_**-3,** **F-1,** **F-2,** **F-3**, respectively, with their measured LUMO energy levels being −2.17 eV, −2.10 eV, −2.11 eV, −1.92 eV, −2.06 eV, and −2.08 eV. These estimations were found to be in good agreement with the theoretical calculations, as shown in Table 2. Small variations in the structure of the bidentate ligand can result in slight perturbations in the HOMO energy level. Notably, when the trifluoromethyl group in the bidentate ligand is replaced by a fluorine atom, there is a gradual increase in the HOMO energy level. In addition, no significant reduction peak was observed for the complex in the solvent, which aligns with the previously reported such structural Ir(III) complexes^18^. This absence of pronounced reduction waves suggests that the complex is difficult to reduce, resulting in a higher LUMO energy level and a larger HOMO-LUMO band gap. This characteristic is advantageous for achieving deep blue emission in Ir(III) complexes.

**Table 2:** Experimental and accordingly computational frontline orbital (HOMO/LUMO) energy level data of all Ir(III) complexes

Complex	Experimental values (eV)				Computational values (eV)		
	E_ox_	HOMO	LUMO	Bandgap	HOMO	LUMO	Bandgap
**CF**_**3**_**-1**	+1.01	−5.38	−2.17	3.21	−5.26	−1.57	3.69
**CF**_**3**_**-2**	+1.11	−5.49	−2.10	3.39	−5.39	−1.60	3.79
**CF**_**3**_**-3**	+1.18	−5.57	−2.11	3.46	−5.46	−1.64	3.82
**F-1**	+0.89	−5.28	−1.92	3.36	−5.19	−1.41	3.78
**F-2**	+1.02	−5.43	−2.06	3.37	−5.33	−1.44	3.89
**F-3**	+1.10	−5.50	−2.08	3.42	−5.40	−1.49	3.91

### Theoretical calculation

In order to investigate the distribution of electron and molecular orbitals within Ir(III) complexes, theoretical calculations were conducted using density functional theory (TD-DFT) with the B3LYP/6-31 G(d,p) method, the LanL2DZ basis set, and Gaussian 16 software. The complexes were analyzed to obtain the distribution data of HOMO/LUMO and several similar orbitals, which are presented in Table 2. Additionally, the optimized molecular structure and electron distribution diagram are depicted in Fig. 1c and Supplementary Tables S63–S71.

For the Ir(III) complexes, the energy differences between the lowest triplet excited states (T_1_) and the ground states (S_0_) were determined as follows, as shown in Supplementary Table S6: 418 nm, 417 nm, 411 nm, 413 nm, 409 nm, and 404 nm for complexes **CF**_**3**_**-1,** **CF**_**3**_**-2,** **CF**_**3**_**-3,** **F-1,** **F-2,** **F-3**, respectively. These values align with the trend observed in the experimental emission wavelengths (456 nm, 437 nm, 425 nm, 442 nm, 419 nm, and 416 nm).

Theoretical calculations reveal that the spin density in all Ir(III) complexes is primarily located on the bidentate ligand and partially on the Ir(III) metal center. The HOMO is mainly found on the benzene ring of the tridentate ligand NHC and the bidentate ligand C^C, while the LUMO is predominantly situated on the nitrogen-hybridization carbene of the bidentate ligand. **CF**_**3**_**-1/-2/-3** share the same -CF_3_ substituent on the carbene moiety but differ in benzene ring substituents (-Me₂, -H, -F). Their HOMO energies show a clear downward trend: −5.26 eV (**CF**_**3**_**-1**) → −5.39 eV (**CF**_**3**_**-2**) → −5.46 eV (**CF**_**3**_**-3**). This is because electron-donating ability decreases in the order -Me₂ > -H > -F. Also, **CF3-1** and **F-1** have the same -Me₂ substituent on the benzene ring but different ones on the carbene moiety (-CF_3_, -F), and their HOMO energies increases (−5.26 eV vs. −5.19 eV). This is due to their different electron-withdrawing ability (-CF_3_ > -F). LUMO energy is dominated by substituents on the bidentate ligand’s N-heterocyclic carbene. **CF**_**3**_**-1/-2/-3** all bear -CF_3_ on the carbene moiety, and their LUMO energies only change slightly: −1.57 eV (**CF**_**3**_**-1**) → −1.60 eV (**CF**_**3**_**-2**) → −1.64 eV (**CF**_**3**_**-3**). However, significant variations exist between **CF3-1** and **F-1** (−1.57 eV vs. −1.41 eV). This is due to -F’s weaker electron-withdrawing ability, thus to stabilize the unoccupied LUMO orbital.

Analysis of natural transition orbitals (NTO) for S_0_- > T_1_ transition demonstrates that excitation modes differ among complexes: charge transfer (CT) excitation for **CF**_**3**_**-1,** **CF**_**3**_**-2,** **CF**_**3**_**-3**, and locally excited (LE) for **F-1,** **F-2,** **F-3**, respectively. The charge characters are analyzed using IFCT calculations (Mulliken-like method). The results (Supplementary Table S12) directly support our transition assignments. F-series complexes show a dominant LE contribution (52.3–57.1%), while CF_3_-series complexes have dominant CT (~94.5–95.0%). This aligns with the localized electron/hole density on the bidentate ligand in F-series and delocalized density in CF_3_-series, as shown in Fig. 1c. The LE contribution mostly attributes to the local excitation within bidentate ligand C^C. And the CT contributions are composed of the ^3^MLCT (Ir→C^C) and ^3^LLCT (pbib→C^C).

The permanent dipole moments (PDM) of T_1_ and the transition dipole moments (TDM) from T_1_ to S_0_ states were calculated using time-dependent density functional theory (TD-DFT) with the B3LYP functional, the def2-SVP basis set for Ir, the SARC/J auxiliary basis set, and the ZORA (Zero - Order Regular Approximation) method to account for relativistic effects, all implemented in the Orca software. The results are presented in Fig. 2c and Table 3. The PDMs of all Ir(III) complexes were oriented within the plane of the bidentate ligands, showing varying magnitudes. Interestingly, the Ir(III) complexes with CT excited states exhibited high PDM values: 3.35 Debye for **CF**_**3**_**-1**, 2.74 Debye for **CF**_**3**_**-2**, and 2.67 Debye for **CF**_**3**_**-3**. In contrast, the Ir(III) complexes with LE states exhibited even higher PDM values: 4.38 Debye for **F-1**, 4.00 Debye for **F-2**, and 4.21 Debye for **F-3**. A large PDM could intensify aggregation of Ir(III) complexes and reduce interactions with the host materials, leading to concentration quenching in OLED devices^30^. However, TDMs from T₁ to S₀ states (${\vec{{\boldsymbol{\Delta }}{\boldsymbol{\mu }}}}_{{T}_{1}{\boldsymbol{\to }}{{\rm{S}}}_{0}}$) were 0.60, 0.74, 0.55, 0.45, 0.37, and 0.29 Debye for **CF**_**3**_**-1,** **CF**_**3**_**-2,** **CF**_**3**_**-3,** **F-1,** **F-2**, and **F-3**, respectively, indicating higher values for Ir(III) complexes with CT excited states. The radiative emission rate (${k}_{r}$), defined as the transition from the electronic triplet (T_1_) to the ground state of S_0_ of these Ir(III) complexes, is predominantly occurs via a triplet metal-to-ligand-charge transfer (^3^MLCT) transition^30^. The ${k}_{r}$ is determined by the energy difference ($\Delta {E}_{{T}_{1}\to {S}_{0}}$) and transition dipole moment from T₁ to S₀ states (${\vec{{\boldsymbol{\Delta }}{\boldsymbol{\mu }}}}_{{T}_{1}{\boldsymbol{\to }}{{\rm{S}}}_{0}}$), and can be expressed as^31^,

$${k}_{r}=\frac{64\,{\pi }^{4}}{3{\hslash }^{4}{c}^{2}}{\Delta E}_{{T}_{1\,}{\to S}_{0}\,}^{3}{\left|{\vec{{\boldsymbol{\Delta }}{\boldsymbol{\mu }}}}_{{T}_{1}{\boldsymbol{\to }}{{\rm{S}}}_{0}}\right|}^{2}$$

Where $c$ is the speed of light. Therefore, ${k}_{r}$ is inherently dependent on ${|{\vec{{\boldsymbol{\Delta }}{\boldsymbol{\mu }}}}_{{T}_{1}{\boldsymbol{\to }}{{\rm{S}}}_{0}}|}^{2}$ since all these six Ir complexes have very close energy difference $\Delta {E}_{{T}_{1}\to {S}_{0}}$ with deep emission in film between 426 nm and 459 nm (Table 1). Therefore, the larger transition dipole moment from T₁ to S₀ states (${\vec{{\boldsymbol{\Delta }}{\boldsymbol{\mu }}}}_{{T}_{1}{\boldsymbol{\to }}{{\rm{S}}}_{0}}$) for **CF**_**3**_**-2** inherently accelerate ${k}_{r}$ as shown in Fig. 2c, which is consistent with the phosphorescent efficiency listed in Table 2. Additionally, there is a strong correlation between balanced charge mobility and a larger dipole moment, suggesting that **CF**_**3**_**-2** is expected to exhibit more balanced charge transport properties due to its larger transition dipole moment of 0.74 Debye.

**Table 3:** The permanent dipole moments (PDMs) of T_1_ and transition dipole moments (TDMs) from T_1_ to S_0_ for the Ir(III) complexes by calculations based on their corresponding T_1_ states

Complex	State	x	y	z	|$\vec{{\boldsymbol{\mu }}}$_*T1*_ | (Debye)	|$\vec{{\boldsymbol{\Delta }}{\boldsymbol{\mu }}}$_(T1_${\boldsymbol{\to }}$_S0)_|(Debye)
**CF**_**3**_**-1**	T_1_	−0.635	−0.006	1.153	3.35	0.60
**CF**_**3**_**-2**	T_1_	0.739	0.003	0.784	2.74	0.74
**CF**_**3**_**-3**	T_1_	0.962	0.003	0.417	2.67	0.55
**F-1**	T_1_	−1.418	0.000	0.981	4.38	0.45
**F-2**	T_1_	−1.393	0.000	0.736	4.00	0.37
**F-3**	T_1_	−1.629	0.000	0.289	4.21	0.29

### Electroluminescence

**Fig. 3:**
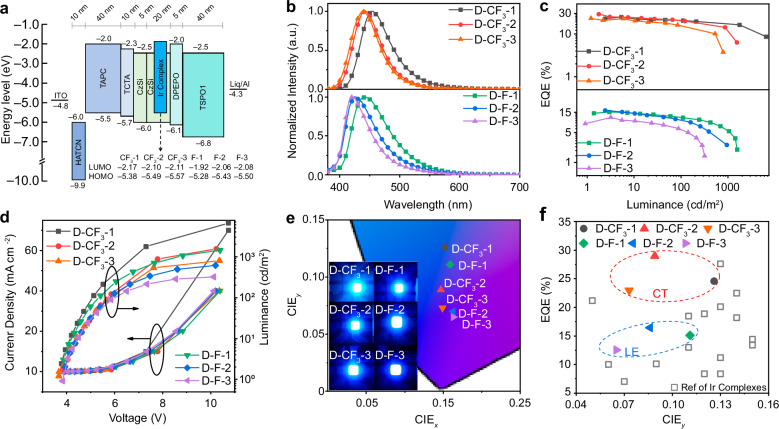
The electroluminescent performances of the studied Ir(III) complexes. **a** The device structure with the energy level; **b** Electroluminescence spectrum, **c** *EQE*-Luminance Characteristics, **d** Current density–voltage–luminance (J–V–L) characteristics and **e** CIE coordinates and according device luminance figures of Ph-OLEDs **D-CF**_**3**_**-1/2/3** and **D-F-1/2/3**; **f** *EQE*-CIE_y_ characteristic curve of reported references about deep-blue Ph-OLED based on Ir(III) complexes

The blue Ph-OLED device was prepared using the vacuum evaporation method employing these six Ir(III) complexes as dopants. The optimized device structure is ITO/HAT-CN (10 nm)/TAPC (40 nm)/TCTA (10 nm)/CzSi (5 nm)/CzSi: Ir(III) complexes (12.5%) (20 nm)/DPEPO (5 nm)/ TSPO1 (40 nm)/Liq (2 nm)/Al (120 nm), as shown in Fig. 3a, along with the molecular structures and energy levels of all the materials involved. Six Ir(III) complexes **CF**_**3**_**-1,** **CF**_**3**_**-2,** **CF**_**3**_**-3,** **F-1,** **F-2,** **F-3** were used as emitters in the emissive layer, and the devices were labeled **D-CF**_**3**_**-1/2/3** and **D-F-1/2/3** accordingly. Based on prior experiments, CzSi was selected as the host material due to its high triplet energy and excellent thermal stability^19^. In this device structure, HAT-CN and Liq serve as hole and electron injection layers, respectively, while TAPC and TCTA efficiently inject and transport holes due to their favorable energy levels. To prevent exciton quenching and balance carrier concentration, a 5 nm DPEPO layer is incorporated as a hole-blocking layer (HBL), forming an exciplex interface with the adjacent electron-transporting layer. TSPO1, containing a diphenylphosphine oxide group, functions as the primary electron transport material^27^. Holes encounter a CzSi layer before reaching the emissive region, which facilitates uniform triplet exciton distribution, enhances electron blocking, and improves hole injection into the emissive layer—ultimately boosting external quantum efficiency (*EQE*). As the host material, CzSi features a lower HOMO level than the Ir(III) dopants and a symmetric structure that promotes efficient exciton recombination with the twisted [3 + 2 + 1] coordinated Ir(III) complexes. A 12.5% dopant concentration was chosen based on pre-experimental optimization to achieve the best device performance^19^.

Figure 3b–d and Supplementary Fig. S73 illustrate the electroluminescent (EL) characteristics of the devices including the EL spectra, *EQE*-luminance plots, and current density-voltage-luminance curve (J-V-L). As shown in Fig. 3b, all Ir(III) complexes exhibit emission in the deep-blue region with EL emission peaks ranging from 423 to 454 nm. This deep-blue emission is driven by the dopant Ir(III) complexes, which incorporate bidentate ligands modified by either strong electron-withdrawing group (-CF_3_/-F) or an electron-donating methyl group. Figure 3c reveals that the **D-CF**_**3**_**-1/2/3** devices consistently outperform their **D-F-1/2/3** counterparts in terms of *EQE*, with **D-CF**_**3**_**-1** has the maximum luminance of 6542 cd m^−2^. The EL images inserted in Fig. 3e clearly show a saturated deep-blue emission across all devices, closely matching the luminescence observed for **CF**_**3**_**-1,** **CF**_**3**_**-2,** **CF**_**3**_**-3,** **F-1,** **F-2,** **F-3** in solution. This confirms that the EL behavior of the device originates from the triplet excited state of the dopant Ir(III) complex. The CIE coordinates of OLEDs are summarized in Table 4, demonstrating successfully tuning of the CIE_y_ value from 0.126 to as low as 0.065. Notably, **D-CF**_**3**_**-2** device exhibits CIE coordinates (0.147, 0.089), which are remarkably close to NTSC deep-blue standard of (0.149, 0.085)^32^. In addition to its ideal color purity, **D-CF**_**3**_**-2** achieves outstanding device performance, including an *EQE*_max_ of 29.0%, a maximum current efficiency (CE) of 17.7 cd A^−1^, a maximum power efficiency (PE) of 14.4 lm W^−1^, and maximum luminance of 1554 cd m^−2^. These results highlight the excellent potential of **D-CF**_**3**_**-2** as a dopant material for high-performance, saturated deep-blue phosphorescent OLEDs, making it a strong candidate for next-generation display applications.

**Table 4:** The EL performance parameters of devices based on the phosphor [3 + 2 + 1] coordinated Ir(III) complexes

Device	Drive voltage [V]^a^	CE/PE/EQE [cd A^−^^1^]/ [lm W^−1^]/[%]^b^	CE/PE/EQE [cd A^−1^]/ [lm W^−1^]/[%]^c^	CIE [x,y]^c^	MAX luminance [cd m^−2^]	EL Peak^c^	LT_50_^d^	LT_50_^e^
**D-CF**_**3**_**-1**	3.78	26.7/21.3/24.6	17.7/7.6/16.3	(0.152, 0.126)	6542	454		
**L-CF**_**3**_**-1**	2.57	10.4/10.2/8.5	9.9/7.5/8.5	(0.132, 0.131)	9217	460	662	3875
**H-CF**_**3**_**-1**	2.81	21.9/25.0/23.4	16.2/8.5/17.6	(0.120, 0.120)	6474	472	15	2127
**D-CF**_**3**_**-2**	3.84	17.7/14.4/29.0	8.7/3.5/14.3	(0.147, 0.089)	1554	443		
**H-CF**_**3**_**-2**	3.04	10.3/11.4/14.3	9.4/5.7/13.8	(0.146, 0.067)	4093	452	8	373
**D-CF**_**3**_**-3**	3.68	12.1/10.3/23.0	5.3/2.2/10.1	(0.149, 0.073)	793	438		
**D-F-1**	3.88	14.9/11.1/15.1	6.9/2.8/7.0	(0.159, 0.111)	1579	443		
**D-F-2**	3.92	12.1/9.8/16.4	4.0/1.5/5.3	(0.163, 0.085)	608	427		
**D-F-3**	3.84	7.5/5.9/12.6	2.6/1.0/4.3	(0.163, 0.065)	319	423		

^a^At 1 cd m^−2^

^b^The maximum current efficieny, power efficiency and external quantum efficiency (*EQE*)

^c^At 10 mA cm^−2^

^d^**L-CF**_**3**_**-1**: L_0_ = 267 cd m^−2^; **H-CF**_**3**_**-1**: L_0_ = 1580 cd m^−2^; **H-CF**_**3**_**-2**: L_0_ = 870 cd m^−2^)

^e^At 100 cd m^−2^, T_50_ = T_0_$\times$[(L_0_/100 cd m^-2^]^*n*^), *n* = 1.8^36,37^]

**D-CF**_**3**_**-1** and **D-CF**_**3**_**-3** devices exhibit excellent performance, achieving *EQE*_max_ values of 24.6% and 23.0%, respectively, due to their dopants sharing a similar C^C ligand with the trifluoromethyl-modified carbene of **D-CF**_**3**_**-2**. **D-CF**_**3**_**-3** emits an extremely deep blue light with a CIE_y_ value of 0.073. In contrast, devices **D-F-1/2/3** although less efficient, still achieving extremely deep blue emission with CIE_y_ values ranging from 0.065 to 0.111. Their reduced efficiency is attributed to the large bandgaps of **F-1/2/3**, respectively, resulting in LE-state-dominated energy transfer and slower intersystem crossing (ISC) due to larger singlet-triplet energy gaps (as illustrated in Fig. 1a and Table 2)^33^. In addition, the large permanent dipole moments (PDM) values of **F-1,** **F-2**, and **F-3** complexes, as shown in Table 3, may induce molecular aggregation and weaken host-guest interactions with CzSi. On the other hand, **CF**_**3**_**-1,** **CF**_**3**_**-2** and **CF**_**3**_**-3** benefit from higher transition dipole moments (TDM), as presented in Fig. 2c, which correlate with faster radiative decay rates, reduced exciton accumulation, and suppressed triplet quenching—thus enhancing device efficiency.

Overall, the deep-blue Ph-OLEDs based on **CF**_**3**_**-1/2/3** and **F-1/2/3** Ir (III) complexes achieve *EQE*_max_ values ranging from 12.6% to 29.0%, which align with PLQY, *k*_*r*_ and TDMs. While PLQY describes the static ratio of radiative to total decay in a film, *k*_*r*_ could directly quantify how quickly excitons are converted to photons—critical for avoiding non-radiative losses, such as suppression of exciton quenching and reduction of exciton diffusion loss. In addition, as shown in Fig. 2c, *k*_*r*_ is directly correlated with TDMs as an indicator of horizontal dipole ratios. A larger TDM implies a higher density of horizontally aligned dipoles. This explains why **CF**_**3**_**-2** (largest TDM) achieves the highest EQE—not only due to faster *k*_*r*_, but also better outcoupling efficiency. As summarized in Fig. 3f and Supplementary Table S13, these devices are among the most efficient Ir (III)-based deep-blue OLEDs reported to date. These high performances are attributed to several structural advantages of the emitters: short phosphorescence lifetimes that minimize triplet-triplet annihilation (TTA), and a sterically hindered [3 + 2 + 1] coordination geometry that increases intermolecular distances and reduces self-quenching. Furthermore, the incorporation of nitrogen-hybridized carbene-containing bidentate ligands effectively lowers the LUMO energy level, promoting efficient electron injection and transport. These combined features contribute to improved charge balance and enhanced overall device performance.

**Fig. 4:**
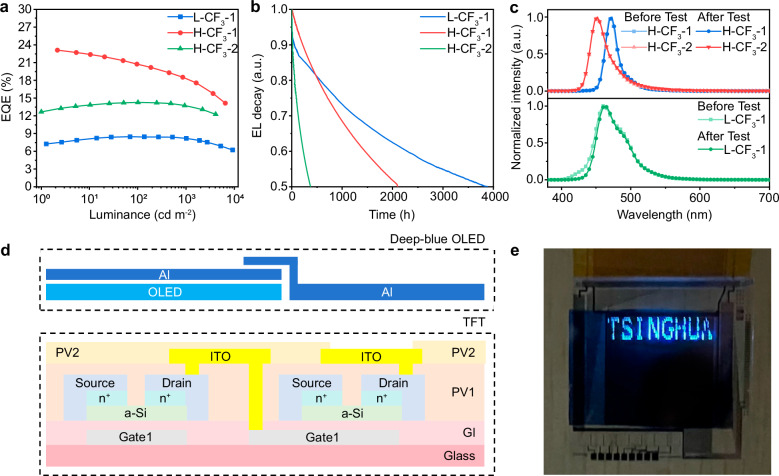
Deep-blue applications of Ir (III) complexes. **a** EQE-Brightness characteristics of **L-CF**_**3**_**-1**, **H-CF**_**3**_**-1/2**; **b** Device lifetime curves at initial luminance of 100 cd m^−2^ of **L-CF**_**3**_**-1** (**CF**_**3**_**-1** as an emitter), **H-CF**_**3**_**-1** (**CF**_**3**_**-1** as a sensitizer and *v*-DABNA as an emitter) and **H-CF**_**3**_**-2** (**CF**_**3**_**-2** as a sensitizer and DOB2-DABNA-A as an emitter), **c** Electroluminescence spectrum (the different curves refer to **L-CF**_**3**_**-1**, **H-CF**_**3**_**-1/2** before or after the stability measurement of several hours); **d** Schematic illustration of the cross-section of the microdisplay device with top-emitting deep-blue OLED-on-TFT; **e** The “Tsinghua” demonstration at this home-built deep-blue OLED-on-TFT chip operated at the switch state with a resolution of 94 PPI (pixel size 270 μm × 270 μm)

To further investigate the stability of devices incorporating asymmetric Ir(III) complexes, a hyper-OLED device was fabricated with the following structure: ITO/HAT-CN (10 nm)/ BCFN (60 nm)/SiCzCz (5 nm)/ SiCzCz : 30% SiTrzCz_2_ : 12.5% Ir(III) complex : 1% TADF (35 nm)/ mSiTrz (5 nm)/ mSiTrz : 50% Liq (30 nm)/ Liq (2 nm)/ Al (120 nm). The corresponding energy levels of each layer is shown in Supplementary Fig. S74. To optimize energy transfer from host to emitter based on HOMO/LUMO alignment, **CF**_**3**_**-1** was selected as a sensitizer for *v*-DABNA^34^ in the fabrication of hyper-OLED **H-CF**_**3**_**-1**, while **CF**_**3**_**-2** served as a sensitizer of DOB2-DABNA-A^35^ for hyper-OLED **H-CF**_**3**_**-2**. The EL performance of these hyper-OLED is presented in Fig. 4a–c and Supplementary Fig. S74, with detailed data summarized in Table 4. Both Hyper-OLEDs exhibit deep-blue emission, with **H-CF**_**3**_**-1** and **H-CF**_**3**_**-2** showing peak wavelengths at 472 and 452 nm, respectively, and narrow FWHM of 20 and 28 nm. The emission profiles remained stable after lifetime measurements, as shown in Fig. 4a, confirming effective sensitization by these two Ir(III) complexes. Significantly, hyper-OLED **H-CF**_**3**_**-1** based on **CF**_**3**_**-1** could reach an excellent device lifetime of LT_50_ = 2127 hours and **H-CF**_**3**_**-2** has obtained device lifetime of LT_50_ = 373 h at L = 100 cd m^−2^, as shown in Fig. 4b. **H-CF**_**3**_**-1** demonstrated excellent performance with an *EQE*_max_ of 23.4%, while **H-CF**_**3**_**-2** achieved a deep-blue color with CIE_xy_ of (0.146, 0.067), an *EQE*_max_ of 14.3% and minimal efficiency roll-off as shown in Fig. 4a.

To further validate the intrinsic stability of the Ir(III) complex **CF**_**3**_**-1**, a control Ph-OLED device (**L-CF**_**3**_**-1**) without a TADF sensitizer was fabricated using the structure: HIT:50% HT (10 nm)/HT (60 nm)/EB (5 nm)/BH:12.5% **CF**_**3**_**-1** (20 nm)/HB (5 nm)/ETL:50% Liq (30 nm)/Liq (2 nm)/Al (120 nm). The HOMO-LUMO energy levels and molecular structures are provided in Supplementary Fig. S76a, b, with device performance shown in in Fig. 4a, b and Supplementary Fig. S76c, d. While the **L-CF**_**3**_**-1** achieved a relatively modest *EQE*_*max*_ of 8.5%, it exhibited exceptional operational stability, with a device lifetime of LT_50_ = 3875 hours at L = 100 cd m^-2^.

### Applications

To investigate deep-blue OLED microdisplay applications, top-emitting arrays of OLEDs employing the **CF**_**3**_**-2** complex were fabricated and demonstrated on thin-film transistor (TFT) array as shown in Fig. 4d. The pixel density was 270 × 270 μm, and the active area of the panel measured 24 mm diagonally, achieving a resolution of 94 PPI. The bonding area of the OLED-on-TFT devices was prepared using thermo-compression technology and interfaces with a smartphone operating system to control the on-off states of the light-emitting units. The on state of the TFT was activated by a device voltage (V_dd_) of 5 V, followed by inputting a power signal to drive the deep-blue OLED. As shown in Fig. 4e, the panel was successfully operated, displaying deep-blue patterns of “Tsinghua”. This OLED-on-TFT device thus provides a platform for further exploration of the potential of Ir(III) complexes in deep-blue microdisplay applications.

## Discussion

In summary, we have successfully synthesized a series of highly asymmetric [3 + 2 + 1] coordinated Ir(III) complexes (**CF**_**3**_**-1,** **CF**_**3**_**-2,** **CF**_**3**_**-3,** **F-1,** **F-2** and **F-3)** as phosphorescent emitters for deep-blue OLED applications. These complexes adopt octahedral coordination geometries, in which the tridentate NHC ligand is oriented approximately perpendicular to the bidentate C^C ligand, effectively enabling precise emission tuning within the deep-blue region (416–456 nm at room temperature). The introduction of strong electron-withdrawing substituents, specifically -CF_3_ and -F on the C^C bidentate ligand distinctly modulates the excited-state characteristics: CF_3_-substituted complexes exhibit predominant CT excited states, whereas F-substituted complexes feature predominantly LE states. Remarkably, complexes with CT excited states display significantly enhanced transition dipole moment from T₁ to S₀ states (${\vec{{\boldsymbol{\Delta }}{\boldsymbol{\mu }}}}_{{T}_{1}{\boldsymbol{\to }}{{\rm{S}}}_{0}}$), inherently accelerating radiative decay and greatly enhancing phosphorescent efficiency. Notably, the Ph-OLED employing the **CF**_**3**_**-2** complex achieved outstanding performance with an *EQE*_max_ of 29.0% and CIE_x,y_ coordinates of (0.147, 0.089), fulfilling the NTSC standards for blue displays. Furthermore, hyper-OLEDs based on **CF**_**3**_**-2** as a sensitizer achieved an LT_50_ of 373 h at L = 100 cd m^−2^ with CIE_x,y_ of (0.146, 0.067). Even more remarkably, the hyper-OLED with **CF**_**3**_**-1** as the sensitizer demonstrated an outstanding stability with CIE_x,y_ of (0.120, 0.120) and LT_50_ of 2127 h at L = 100 cd m^−2^. A control device fabricated without sensitization using **CF**_**3**_**-1** further confirms its intrinsic material stability by exhibiting a remarkable operational lifetime of LT_50_ of 3875 h at L = 100 cd m^−2^ with CIE_x,y_ of (0.132,0.131).

Additionally, Ph-OLEDs employing Ir(III) complexes with LE excited states achieved deeper blue emission spectra with CIE_y_ as low as 0.065. Ultimately, these advanced phosphorescent materials have been successfully integrated into OLED-on-TFT micro-display technology, demonstrating programmable high-resolution (94 PPI, 270 × 270 μm) display capabilities, including the clear display of “**Tsinghua**” controlled via a smartphone application. This work opens a promising new pathway toward achieving high-performance and stable deep-blue OLEDs for practical display technology such as advanced microdisplay applications.

## Materials and methods

All commercially available reagents were used directly without further purification. The phosphorescent Ir complexes were synthesized by co-authors, and the detailed process was listed in the supporting information. The MR-TADF materials were provided by the research group of Chihaya Adachic and Takuji Hatakeyama. Single crystals of Ir complexes suitable for X-ray diffraction analysis were grown using the anti-solvent diffusion method, and the resulting crystals were isolated and used for structural characterization. The redox properties and energy levels of Ir complexes were determined using cyclic voltammetry with a concentration of 10⁻⁵ mol L⁻¹ in chromatographic-grade acetonitrile (CH₃CN). UV-vis absorption spectra were obtained using a Cary 5000 UV-vis-NIR spectrophotometer (Agilent, USA) at room temperature with a concentration of 10^−5^ M. Emission spectra were measured at different conditions using an Edinburgh Instruments Ltd. FS5 spectrofluorometer. Phosphorescent lifetime decay curves were recorded using an Edinburgh FLSP920 spectrometer with time-dependent single photon counting technique, using a sodium lamp as the light source. Device fabrication was conducted in a FS-450 chamber (Suzhou Fangsheng). Device characteristics, including EL spectra, *J-V-L* curves, and CIE coordinates, were measured using a Keithley 2400 system. Device lifetimes were assessed with an OLED aging lifetime tester (ADVANTECH, 610L). Details of materials syntheses, NMR spectra, ESI-MS spectra, TGA data, single crystal analyses, supportative photophysical and electrochemical data, computational study and TD-DFT results, OLED performance data of all iridium complexes were listed in the supporting information.

## Supplementary information

supporting figures and tables

## Data Availability

All data needed to evaluate the conclusions in the paper are present in the paper and/or the Supplementary Materials. Additional data related to this paper may be requested from the corresponding author.
