# Supplementary material for: High-efficiency and stable deep-blue iridium phosphorescent OLEDs with enhanced charge transfer dynamics
Source: Light Sci Appl. 2026 Jun 2;15:259. doi: 10.1038/s41377-026-02264-y (PMC13230643; doi:10.1038/s41377-026-02264-y)
Supplement: Supplementary file 1 — supporting figures and tables [file 41377_2026_2264_MOESM1_ESM.docx]

# Supporting information

**High-Efficiency and Stable Deep-Blue Iridium Phosphorescent OLEDs with Enhanced Charge Transfer Dynamics**

**Author:** Siqi Li, ^ⴕ,^ ^a^ Kai-Ning Tong, ^ⴕ,a^ Meng Zhang, ^ⴕ,^ ^a^ Wei He, ^a^ Chengcheng Wu, ^a,*^ Junki Ochi, ^b^ Di Wu, ^b^ Kefei Shi, ^a^ Xin Wang, ^a^ Singyeong Jung, ^a^ Feiyu Kang, ^a,*^ Chihaya Adachi, ^c,*^ Takuji Hatakeyama, ^b,*^ and Guodan Wei ^a,*^

^ⴕ^S.Q. L., K.N. T. and M. Z. contributed equally to this work.

^a^ Institute of Materials Research, Tsinghua Shenzhen International Graduate School, Tsinghua University, Shenzhen 518055, China

^b^ Department of Chemistry, Graduate School of Science, Kyoto University, Kyoto 606-8502, Japan

^c^ Center for Organic Photonics and Electronic Research (OPERA), Kyushu University, Fukuoka 819-0395, Japan

^*^ Correspondence should be addressed to:[weiguodan@sz.tsinghua.edu.cn](mailto:weiguodan@sz.tsinghua.edu.cn); [wucc@tsinghua.edu.cn;](mailto:wucc@tsinghua.edu.cn;) fykang@sz.tsinghua.edu.cn; [hatake@kuchem.kyoto-u.ac.jp](mailto:hatake@kuchem.kyoto-u.ac.jp); [adachi@cstf.kyushu-u.ac.jp](mailto:adachi@cstf.kyushu-u.ac.jp)

General Information

All reagents used in this study were of commercial grade and utilized without further purification. Nuclear magnetic resonance (NMR) spectra for protons (^1^H), fluorine (^19^F), and carbon (^13^C) were acquired on a Bruker 400 / 600 MHz spectrometer utilizing tetramethylsilane (TMS) as the internal reference standard. Chemical shifts (δ) are reported in parts per million (ppm) relative to TMS. The multiplicity of NMR signals is denoted as follows: s for singlet, d for doublet, t for triplet, q for quartet, and m for multiplet. Coupling constants (J) are expressed in Hertz (Hz). Mass spectrometry was conducted using electrospray ionization (ESI) on a Thermo Scientific Exactive Benchtop LC/MS Orbitrap Mass Spectrometer. Absorption and phosphorescence spectra were recorded using a UV-vis spectrophotometer (Cary 5000 UV–vis–NIR, Agilent, USA) and a spectrofluorometer (Edinburgh Instruments Ltd FS5), respectively. The photoluminescence quantum efficiency (PLQY) was determined using an absolute PL quantum yield measurement system (C11347-01 Hamamatsu Photonics) under a nitrogen atmosphere with an excitation wavelength of 350 nm. Transient decay measurements were performed with a Hamamatsu C11367-03 Quantaurus-Tau fluorescence lifetime measurement system. Electrochemical characterization was carried out using a PalmSens4 electrochemical workstation, with a platinum-carbon electrode serving as the working electrode, a platinum wire as the counter electrode, and a saturated calomel electrode (SCE) in saturated KCl aqueous solution as the reference electrode. The cyclic voltammograms were calibrated to the ferrocene / ferrocenium couple at a scan rate of 100 mV s⁻¹. Thermogravimetric analysis (TGA) was conducted on a Mettler TGA2 thermogravimeter, monitoring weight loss from 25 °C to 800 °C at a heating rate of 10 °C/min under a nitrogen atmosphere, with an isothermal hold at 100 °C for 15 minutes before the final heating segment.

OLED Fabrication

Indium-tin oxide (ITO) coated glass substrates were initially cleaned sequentially in deionized water, acetone, and ethanol, followed by drying in an oven and treatment with ultraviolet ozone for 20 minutes. Device fabrication was executed in a FS-450 (Suzhou Fangsheng) chamber. All organic layers were thermally evaporated at a rate of 0.5-1.5 Å s⁻¹ under a vacuum pressure of approximately 7.5 × 10⁻⁷ Torr. A lithium quinolate (Liq) layer (2 nm) was deposited at a rate of 0.2 Å s⁻¹. The aluminum (Al) cathode was deposited at a rate of 4 Å s⁻¹, with an active area of the diode segments measuring 3×3 mm². Device performance, including electroluminescence (EL) spectra, current density-voltage-luminance (*J-V-L*) curves, and Commission Internationale de L'Eclairage (CIE) coordinates, was characterized using a Keithley 2400 semiconductor characterization system.

Materials synthesis


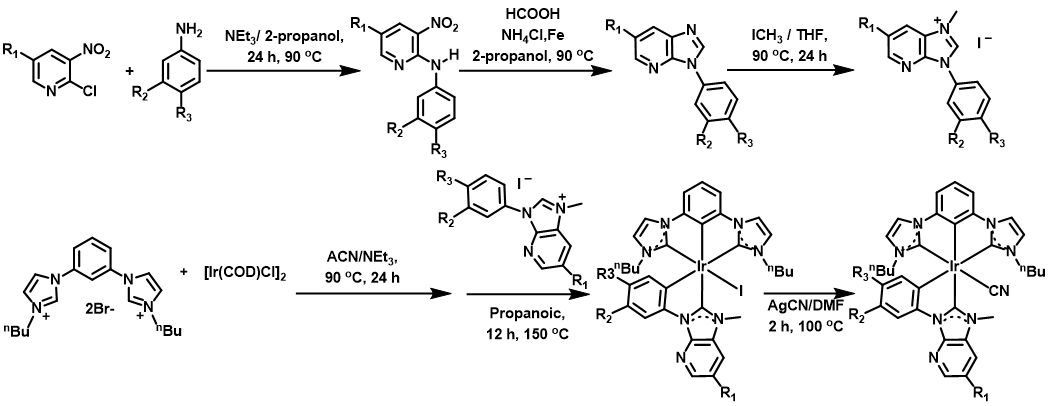


**Figure S1**. Synthetic route of the Iridium(III) complexes

**Supplementary Note 1. Synthesis of the ligands and iridium(III) complexes:**

**N-(3,4-dimethylphenyl)-3-nitro-5-(trifluoromethyl)pyridin-2-amine**:

2-chloro-3-nitro-5-(trifluoromethyl)pyridine (4.2 g, 18.9 mmol), 3,4-dimethylaniline (2.8 g, 22.7 mmol), and triethylamine (2.4 g, 23.6 mmol) were reﬂuxed with 50 mL of 2-propanol for 24 h. Afterward, the reaction mixture was concentrated and extracted with diethyl ether. The resulting product, N-(3,4-dimethylphenyl)-3-nitro-5-(trifluoromethyl)pyridin-2-amine, was isolated with a yield of 49% (3.9 g).

^1^H NMR (400 MHz, Chloroform-*d*) δ 10.15 (s, 1H), 8.74 (d, *J* = 2.3 Hz, 1H), 8.66 (d, *J* = 2.3 Hz, 1H), 7.34−7.31 (m, 2H), 7.18 (d, *J* = 7.9 Hz, 1H), 2.29 (d, *J* = 7.6 Hz, 6H).

**N-(3,4-dimethylphenyl)-5-fluoro-3-nitropyridin-2-amine:**

2-chloro-5-fluoro-3-nitropyridine (3.3 g, 18.9 mmol), 3,4-dimethylaniline (2.8 g, 22.7 mmol), and triethylamine (2.4 g, 23.6 mmol) were reﬂuxed with 50 mL of 2-propanol for 24 h. Afterward, the reaction mixture was concentrated and extracted with diethyl ether. The product, N-(3,4-dimethylphenyl)-5-fluoro-3-nitropyridin-2-amine, was obtained with a yield of 46% (4.0 g)*.*

^1^H NMR (400 MHz, Chloroform-*d*) δ 9.88 (s, 1H), 8.41 (d, *J* = 2.9 Hz, 1H), 8.27 (dd, *J* = 7.9, 2.9 Hz, 1H), 7.33 – 7.26 (m, 2H), 7.15 (d, *J* = 8.0 Hz, 1H), 2.28 (d, *J* = 9.4 Hz, 6H). ^19^F NMR (376 MHz, Chloroform-*d*) δ -139.29.

**3-nitro-N-phenyl-5-(trifluoromethyl)pyridin-2-amine:**

2-chloro-3-nitro-5-(trifluoromethyl)pyridine (4.2 g, 18.9 mmol), aniline (2.1 g, 22.7 mmol), and triethylamine (2.4 g, 23.6 mmol) were reﬂuxed with 50 mL of 2-propanol for 24 h. Afterward, the reaction mixture was concentrated and extracted with diethyl ether. 3-nitro-*N*-phenyl-5-(trifluoromethyl)pyridin-2-amine (4.7 g, 50%) was obtained: ^1^H NMR (400 MHz, Chloroform-*d*) δ 10.27 (s, 1H), 8.76 (d, *J* = 2.3 Hz, 1H), 8.69 (d, *J* = 2.3 Hz, 1H), 7.63 – 7.60 (m, 2H), 7.46 – 7.41 (m, 2H), 7.28 – 7.24 (m, 1H).

**5-fluoro-3-nitro-N-phenylpyridin-2-amine:**

2-chloro-5-fluoro-3-nitropyridine (3.3 g, 18.9 mmol), aniline (2.1 g, 22.7 mmol), and triethylamine (2.4 g, 23.6 mmol) were reﬂuxed with 50 mL of 2-propanol for 24 h. Afterward, the reaction mixture was concentrated and extracted with diethyl ether. 5-fluoro-3-nitro-*N*-phenylpyridin-2-amine (3.2 g, 48%) was obtained:

^1^H NMR (500 MHz, Chloroform-*d*) δ 10.00 (s, 1H), 8.43 (d, *J* = 3.0 Hz, 1H), 8.30 (dd, *J* = 7.9, 2.9 Hz, 1H), 7.60 (d, *J* = 7.6 Hz, 2H), 7.42 – 7.38 (m, 2H), 7.21 – 7.18 (m, 1H).; ^19^F NMR (376 MHz, Chloroform-*d*) δ-138.51.

**N-(4-fluorophenyl)-3-nitro-5-(trifluoromethyl)pyridin-2-amine:**

2-chloro-3-nitro-5-(trifluoromethyl)pyridine (4.2 g, 18.9 mmol), 4-fluoroaniline (2.5 g, 22.7 mmol), and triethylamine (2.4 g, 23.6 mmol) were reﬂuxed with 50 mL of 2-propanol for 24 h. Afterward, the reaction mixture was concentrated and extracted with diethyl ether. *N*-(4-fluorophenyl)-3- nitro-5-(trifluoromethyl)pyridin-2-amine (5.2 g, 51%) was obtained:

^1^H NMR (400 MHz, Chloroform-*d*) δ10.18 (s, 1H), 8.76 (d, *J* = 2.0 Hz, 1H), 8.66 (d, *J* = 1.9 Hz, 1H), 7.60 – 7.50 (m, 2H), 7.18 – 7.07 (m, 2H); ^19^F NMR (376 MHz, Chloroform-*d*) δ -61.45, -115.54.

**5-fluoro-N-(4-fluorophenyl)-3-nitropyridin-2-amine:**

2-chloro-5-fluoro-3-nitropyridine (3.3 g, 18.9 mmol), 4-fluoroaniline (2.5 g, 22.7 mmol), and triethylamine (2.4 g, 23.6 mmol) were reﬂuxed with 50 mL of 2-propanol for 24 h. Afterward, the reaction mixture was concentrated and extracted with diethyl ether. 5-fluoro-*N*-(4-fluorophenyl)-3-nitropyridin-2-amine (5.0 g, 50%) was obtained: ^1^H NMR (400 MHz, Chloroform-*d*) δ 9.90 (s, 1H), 8.41 (d, *J* = 2.9 Hz, 1H), 8.29 (dd, *J* = 7.8, 2.9 Hz, 1H), 7.56 – 7.51 (m, 2H), 7.12 – 7.06 (m, 2H).; ^19^F NMR (376 MHz, Chloroform-*d*) δ-116.97, -138.42.

**3-(3,4-dimethylphenyl)-6-(trifluoromethyl)-3H-imidazo[4,5-b]pyridine**:

*N*-(3,4-dimethylphenyl)-3-nitro-5-(trifluoromethyl)pyridin-2-amine (3.86 g, 12.4 mmol), formic acid (122 g, 2.66 mol), ammonium chloride (11.2 g, 0.21 mol), and iron powder (11.7 g, 0.21 mol) were suspended in 40 mL of 2-propanol and refluxed for 48 h. Afterward, the reaction mixture was concentrated to dryness, washed with 200 mL of a saturated aqueous NaHCO_3_ solution, and extracted with dichloromethane; organics were dried over MgSO_4_, and volatiles were removed. The product was obtained in 87% yield (3.99 g):

^1^H NMR (400 MHz, Chloroform-*d*) δ 8.70 (s, 1H), 8.38 (d, *J* = 16.5 Hz, 2H), 7.44 (s, 1H), 7.39 (d, *J* = 7.9 Hz, 1H), 7.31 (d, *J* = 6.9 Hz, 1H), 2.34 (d, *J* = 9.5 Hz, 6H).

**3-(3,4-dimethylphenyl)-6-fluoro-3H-imidazo[4,5-b]pyridine**:

*N*-(3,4-dimethylphenyl)-5-fluoro-3-nitropyridin-2-amine (3.13 g, 12.4 mmol), formic acid (122 g, 2.66 mol), ammonium chloride (11.2 g, 0.21 mol), and iron powder (100 mesh, 11.7 g, 0.21 mol) were suspended in 40 mL of 2-propanol and refluxed for 48 h. Afterward, the reaction mixture was concentrated to dryness, washed with 200 mL of a saturated aqueous NaHCO_3_ solution, and extracted with dichloromethane; organics were dried over MgSO_4_, and volatiles were removed. The product was obtained in 84% yield (2.78 g):

^1^H NMR (400 MHz, Chloroform-*d*) δ 8.34 (dd, *J* = 2.6, 1.6 Hz, 1H), 8.32 (s, 1H), 7.85 (dd, *J* = 8.6, 2.6 Hz, 1H), 7.46 (d, *J* = 2.3 Hz, 1H), 7.41 (dd, *J* = 8.0, 2.3 Hz, 1H), 7.32 (d, *J* = 8.0 Hz, 1H), 2.36 (d, *J* = 10.7 Hz, 6H); ^19^F NMR (376 MHz, Chloroform-*d*) δ -134.34.

**3-phenyl-6-(trifluoromethyl)-3H-imidazo[4,5-b]pyridine:**

3-nitro-*N*-phenyl-5-(trifluoromethyl)pyridin-2-amine (3.51 g, 12.4 mmol), formic acid (122 g, 2.66 mol), ammonium chloride (11.2 g, 0.21 mol), and iron powder (11.7 g, 0.21 mol) were suspended in 40 mL of 2-propanol and refluxed for 48 h. Afterward, the reaction mixture was concentrated to dryness, washed with 200 mL of a saturated aqueous NaHCO_3_ solution, and extracted with dichloromethane; organics were dried over MgSO_4_, and volatiles were removed. The product was obtained in 89% yield (3.24 g):

^1^H NMR (400 MHz, Chloroform-*d*) δ 8.73 (s, 1H), 8.45 (d, *J* = 1.0 Hz, 1H), 8.39 (s, 1H), 7.75 – 7.71 (m, 2H), 7.62 – 7.58 (m, 2H), 7.51 – 7.46 (m, 1H). ^19^F NMR (376 MHz, Chloroform-*d*) δ -60.29.

**6-fluoro-3-phenyl-3H-imidazo[4,5-b]pyridine:**

5-fluoro-3-nitro-*N*-phenylpyridin-2-amine (2.89 g, 12.4 mmol), formic acid (122 g, 2.66 mol), ammonium chloride (11.2 g, 0.21 mol), and iron powder (11.7 g, 0.21 mol) were suspended in 40 mL of 2-propanol and refluxed for 48 h. Afterward, the reaction mixture was concentrated to dryness, washed with 200 mL of a saturated aqueous NaHCO_3_ solution, and extracted with dichloromethane; organics were dried over MgSO_4_, and volatiles were removed. The product was obtained in 88% yield (2.65 g): ^1^H NMR (400 MHz, Chloroform-*d*) δ 8.39 (s, 1H), 8.36 (dd, *J* = 2.6, 1.5 Hz, 1H), 7.87 (dd, *J* = 8.6, 2.6 Hz, 1H), 7.75 – 7.72 (m, 2H), 7.61 – 7.56 (m, 2H), 7.49 – 7.45 (m, 1H). ^19^F NMR (376 MHz, Chloroform-*d*) δ -133.86.

**3-(4-fluorophenyl)-6-(trifluoromethyl)-3H-imidazo[4,5-b]pyridine**:

*N*-(4-fluorophenyl)-3-nitro-5-(trifluoromethyl)pyridin-2-amine (3.73 g, 12.4 mmol), formic acid (122 g, 2.66 mol), ammonium chloride (11.2 g, 0.21 mol), and iron powder (100 mesh, 17.3 g, 0.31 mol) were suspended in 80 mL of 2-propanol and reﬂuxed for 48 h. Afterward, the reaction mixture was concentrated to dryness, washed with a saturated aqueous NaHCO_3_ solution, and extracted with dichloromethane; organics were dried over MgSO_4_, and volatiles were removed. The product was obtained in 82% yield (3.43 g):

^1^H NMR (400 MHz, Chloroform-*d*) δ 8.73 (s, 1H), 8.42 (s, 2H), 7.74 – 7.69 (m, 2H), 7.34 – 7.28 (m, 2H).; ^19^F NMR (376 MHz, Chloroform-*d*) δ -60.31, -112.05.

**3-(3,4-dimethylphenyl)-1-methyl-6-(trifluoromethyl)-3H-imidazo[4,5-b]pyridin-1-ium iodide (CF_3_pmpMe_2_)**:

3-(3,4-dimethylphenyl)-6-(trifluoromethyl)-3*H*-imidazo[4,5-*b*]pyridine (5.01 g, 17.2 mmol) and methyl iodide (2.4 g, 17.2 mmol) were dissolved in 20 mL of THF and stirred at 60 °C for 72 h in Schlenk tube. Afterward, the precipitate was filtered, washed with diethyl ether, and dried in vacuo. The product was obtained in 38% yield (2.37 g): ^1^H NMR (400 MHz, DMSO-*d*_6_) δ 10.53 (s, 1H), 9.31 (d, *J* = 1.1 Hz, 1H), 9.24 (d, *J* = 1.1 Hz, 1H), 7.66 (d, *J* = 1.8 Hz, 1H), 7.61 (dd, *J* = 8.1, 2.4 Hz, 1H), 7.51 (d, *J* = 8.1 Hz, 1H), 4.24 (s, 3H), 2.36 (s, 6H); ^19^F NMR (376 MHz, DMSO-*d*_6_) δ -58.49, -59.12.

**3-(3,4-dimethylphenyl)-6-fluoro-1-methyl-3H-imidazo[4,5-b]pyridin-1-ium iodide (FpmpMe_2_)**:

3-(3,4-dimethylphenyl)-6-fluoro-3*H*-imidazo[4,5-b]pyridine (4.15 g, 17.2 mmol) and methyl iodide (2.4 g, 17.2 mmol) were dissolved in 20 mL of THF and stirred at 60 °C for 72 h in Schlenk tube. Afterward, the precipitate was filtered, washed with diethyl ether, and dried in vacuo. The product was obtained in 36% yield (2.12 g):

^1^H NMR (400 MHz, DMSO-*d*_6_) δ 10.37 (d, *J* = 2.1 Hz, 1H), 8.89 – 8.88 (m, 1H), 8.81 (dd, *J* = 8.5, 2.4 Hz, 1H), 7.65 (s, 1H), 7.60 (dd, *J* = 8.0, 2.0 Hz, 1H), 7.49 (d, *J* = 8.1 Hz, 1H), 4.15 (s, 3H), 2.36 (s, 6H).; ^19^F NMR (376 MHz, DMSO-*d*_6_) δ ^19^F NMR (376 MHz, DMSO-*d*_6_) δ -127.62 (d, *J* = 3.6 Hz).

**1-methyl-3-phenyl-6-(trifluoromethyl)-3H-imidazo[4,5-b]pyridin-1-ium iodide (CF_3_pmp):**

3-phenyl-6-(trifluoromethyl)-3*H*-imidazo[4,5-b]pyridine (4.52 g, 17.2 mmol) and methyl iodide (2.4 g, 17.2 mmol) were dissolved in 20 mL of THF and stirred at 60 °C for 72 h in Schlenk tube. Afterward, the precipitate was filtered, washed with diethyl ether, and dried in vacuo. The product was obtained in 39% yield (2.31 g):

^1^H NMR (400 MHz, DMSO-*d*_6_) δ 10.62 (s, 1H), 9.33 (d, *J* = 0.9 Hz,1H), 9.25 (d, *J* = 1.0 Hz, 1H), 7.91 (d, *J* = 7.9 Hz, 2H), 7.78 – 7.75 (m, 2H), 7.73 – 7.70 (m, 1H), 4.27 (s, 3H).; ^19^F NMR (376 MHz, DMSO-*d*_6_) δ -59.10.

**6-fluoro-1-methyl-3-phenyl-3H-imidazo[4,5-b]pyridin-1-ium iodide(Fpmp):**

6-fluoro-3-phenyl-3*H*-imidazo[4,5-b]pyridine (3.66 g, 17.2 mmol) and methyl iodide (2.4 g, 17.2 mmol) were dissolved in 20 mL of THF and stirred at 60 °C for 72 h in Schlenk tube. Afterward, the precipitate was filtered, washed with diethyl ether, and dried in vacuo. The product was obtained in 40% yield (2.19 g):

^1^H NMR (400 MHz, DMSO-*d*_6_) δ 11.50 – 11.45 (m, 1H), 9.90 – 9.84 (m, 2H), 8.91 (t, *J* = 8.7 Hz, 2H), 8.75 (t, *J* = 7.6 Hz, 2H), 8.70 – 8.67 (m, 1H), 5.18 (s, 3H); ^19^F NMR (376 MHz, DMSO-*d*_6_) δ -127.59.

**3-(4-fluorophenyl)-1-methyl-6-(trifluoromethyl)-3H-imidazo[4,5-b]pyridin-1-ium iodide (CF_3_pmpF)**:

3-(4-fluorophenyl)-6-(trifluoromethyl)-3*H*-imidazo[4,5-b]pyridine (4.83 g, 17.2 mmol) and methyl iodide (2.4 g, 17.2 mmol) were dissolved in 20 mL of THF and stirred at 60 °C for 72 h in Schlenk tube. Afterward, the precipitate was filtered, washed with diethyl ether, and dried in vacuo. The product was obtained in 39% yield (3.11 g):

^1^H NMR (400 MHz, DMSO-*d*_6_) δ 10.60 – 10.58 (m, 1H), 9.33 (s, 1H), 9.25 (d, *J* = 1.9 Hz, 1H), 7.98 – 7.93 (m, 2H), 7.68 – 7.61 (m, 2H), 4.27 (s, 3H).; ^19^F NMR (376 MHz, DMSO-*d*_6_) δ -59.10, -109.97 – -109.99 (m).

**[(pbib)Ir(CF_3_pmpMe_2_)I]:**

Under the protection of N_2_, **pbib** (0.90 mmol, 435.9 mg) and [Ir(COD)Cl]_2_ (0.45 mmol, 300.0 mg) were added to a mixture of triethylamine (1 mL) and acetonitrile (10 mL) in Schlenk tube. The suspension was heated at 90 °C for 12 h. After cooling, the solvent was distilled by rotary evaporation to give yellow solid. Then the yellow solid was dissolved in 2-ethoxyethanol (10 mL), and **CF_3_pmpMe_2_** (0.90 mmol, 389.7 mg) and triethylamine (1 ml) was added to the solution and heated at 150 ℃ for 24 h under N_2_. After cooling to room temperature, 2-ethoxyethanol was distilled under the low pressure. The crude product was purified by silica gel column (eluent = dichloromethane/petroleum ether 3:1 v/v) to afford the expected product (372.0 mg, 48%).

^1^H NMR (400 MHz, Chloroform-*d*) δ 8.75 (s, 1H), 8.48 (s, 1H), 7.98 (d, *J* = 1.7 Hz, 1H), 7.44 (d, *J* = 2.1 Hz, 2H), 7.24 – 7.20 (m, 3H), 6.69 (d, *J* = 2.1 Hz, 2H), 5.59 (s, 1H), 4.85 (s, 3H), 3.51 – 3.33 (m, 4H), 2.14 (s, 3H), 1.83 (s, 3H), 1.43 – 1.33 (m, 2H), 1.12 – 1.00 (m, 2H), 0.87 – 0.76 (m, 2H), 0.60 – 0.48 (m, 8H); ^19^F NMR (376 MHz, Chloroform-*d*) δ -60.22.

**[(pbib)Ir(CF_3_pmpMe_2_)CN] (CF_3_-1)**:

Under the protection of N_2_, [(pbib)Ir(CF_3_pmpMe_2_)I] (0.34 mmol, 302.9 mg) and the double molar quantity of AgCN (0.68 mmol, 91.6 mg) were dissolved in a round-bottom flask containing 20 mL of *N*,*N*-dimethylformamide (DMF). The suspension was heated at 100 °C under N_2_ for 2 h. After cooling, the solvent was filtered and removed off by rotary evaporation to afford the crude product. The crude product was purified by silica gel column (eluent = dichloromethane/ethyl acetate 3:1 v/v) to afford the expected product. Then, the expected product was recrystallized in CH_2_Cl_2_/diethyl ether (276.9 mg, 97%).

^1^H NMR (400 MHz, Chloroform-*d*) δ ^1^H NMR (400 MHz, Chloroform-*d*) δ 8.77 (dd, *J* = 2.0, 1.0 Hz, 1H), 8.50 (s, 1H), 8.02 (d, *J* = 2.0 Hz, 1H), 7.46 (d, *J* = 2.1 Hz, 2H), 7.31 (dd, *J* = 8.5, 6.9 Hz, 1H), 7.23 – 7.19 (m, 2H), 6.71 (d, *J* = 2.1 Hz, 2H), 5.65 (s, 1H), 4.70 (s, 3H), 3.38 – 3.26 (m, 4H), 2.15 (s, 3H), 1.81 (s, 3H), 1.43 – 1.32 (m, 2H), 1.11 – 1.00 (m, 2H), 0.88 – 0.74 (m, 2H), 0.62 – 0.50 (m, 8H); ^19^F NMR (376 MHz, Chloroform-*d*) δ -60.24; ^13^C NMR (125 MHz, Chloroform-*d*) δ 191.62, 163.72, 146.85, 145.64, 145.25 (2C), 143.34, 136.16, 136.06, 132.41, 129.60, 129.01 (2C), 128.81, 128.74, 128.26, 122.89, 119.06 (2C), 117.70, 117.48, 116.20, 115.85 (2C), 107.65 (2C), 52.56, 50.86 (2C), 33.03, 19.81 (2C), 19.79, 19.76, 13.44 (2C). ESI-MS calcd for C_37_H_38_F_3_IrN_8_ 845.2874; Found: 845.2872.
**[(pbib)Ir(CF_3_pmp)I]**:

Under the protection of N_2_, **pbib** (0.90 mmol, 435.9 mg) and [Ir(COD)Cl]_2_ (0.45 mmol, 300.0 mg) were added to a mixture of triethylamine (1 mL) and acetonitrile (10 mL) in Schlenk tube. The suspension was heated at 90 °C for 12 h. After cooling, the solvent was distilled by rotary evaporation to give yellow solid. Then the yellow solid was dissolved in 2-ethoxyethanol (10 mL), and **CF_3_pmp** (0.90 mmol, 364.5 mg) and triethylamine (1 ml) was added to the solution and heated at 150℃ for 24 h under N_2_. After cooling to room temperature, 2-ethoxyethanol was distilled under the low pressure. The crude product was purified by silica gel column (eluent = dichloromethane/petroleum ether 3:1 v/v) to afford the expected product (342.8 mg, 47%).^1^H NMR (400 MHz, Chloroform-*d*) δ 8.75 (s, 1H), 8.70 (dd, *J* = 7.9, 1.4 Hz, 1H), 8.00 (d, *J* = 1.9 Hz, 1H), 7.45 (d, *J* = 2.1 Hz, 2H), 7.24– 7.23 (m, 3H), 6.88 – 6.84 (m, 1H), 6.70 (d, *J* = 2.0 Hz, 2H), 6.49 (td, *J* = 7.5, 1.3 Hz, 1H), 5.92 (dd, *J* = 7.7, 1.2 Hz, 1H), 4.87 (s, 3H), 3.49 – 3.36 (m, 4H), 1.39 – 1.29 (m, 2H), 1.06 – 0.96 (m, 2H), 0.83 – 0.73 (m, 2H), 0.53 – 0.45 (m, 8H).; ^19^F NMR (376 MHz, Chloroform-*d*) δ -60.24.

**[(pbib)Ir(CF_3_pmp)CN](CF_3_-2)**:

Under the protection of N_2_, [(pbib)Ir(CF_3_pmp)I] (0.34 mmol, 293.4 mg) and the double molar quantity of AgCN (0.68 mmol, 91.6 mg) were dissolved in a round-bottom flask containing 20 mL of *N*,*N*-dimethylformamide (DMF). The suspension was heated at 100 °C under N_2_ for 2 h. After cooling, the solvent was filtered and removed off by rotary evaporation to afford the crude product. The crude product was purified by silica gel column (eluent = dichloromethane/ethyl acetate 3:1 v/v) to afford the expected product. Then, the expected product was recrystallized in CH_2_Cl_2_/diethyl ether (236.7 mg, 95%).

^1^H NMR (400 MHz, Chloroform-*d*) δ 8.76 (s, 1H), 8.72 (d, *J* = 7.9 Hz, 1H), 8.03 (d, *J* = 1.9 Hz, 1H), 7.46 (d, *J* = 2.2 Hz, 2H), 7.31 (dd, *J* = 8.5, 6.9 Hz, 1H), 7.22 – 7.20 (m, 2H), 6.92 – 6.88 (m, 1H), 6.71 (d, *J* = 2.1 Hz, 2H), 6.52 (td, *J* = 7.4, 1.0 Hz, 1H), 5.99 (dd, *J* = 7.3, 1.0 Hz, 1H), 4.75 (s, 3H), 3.39 – 3.28 (m, 4H), 1.39 – 1.28 (m, 2H), 1.06 – 0.95 (m, 2H), 0.83 – 0.73 (m, 2H), 0.55 – 0.47 (m, 8H).; ^19^F NMR (376 MHz, Chloroform-*d*) δ -60.28; ^13^C NMR (125 MHz, Chloroform-*d*) δ 191.62, 163.72, 146.85, 145.64, 145.25 (2C), 143.34, 136.16, 136.06, 132.41, 129.60, 129.01 (2C), 128.81, 128.74, 128.26, 122.89, 119.06 (2C), 117.70, 117.48, 116.20, 115.85 (2C), 107.65 (2C), 52.56, 50.86 (2C), 33.03, 19.81 (2C), 19.79, 19.76, 13.44 (2C). ESI-MS calcd for C_35_H_34_F_3_IrN_8_ 817.2561; Found: 817.2555.
**[(pbib)Ir(CF_3_pmpF)I]**:

Under the protection of N_2_, **pbib** (0.90 mmol, 435.9 mg) and [Ir(COD)Cl]_2_ (0.45 mmol, 300.0 mg) were added to a mixture of triethylamine (1 mL) and acetonitrile (10 mL) in Schlenk tube. The suspension was heated at 90 °C for 12 h. After cooling, the solvent was distilled by rotary evaporation to give yellow solid. Then the yellow solid was dissolved in 2-ethoxyethanol (10 mL), and **CF_3_pmpF** (0.90 mmol, 380.7 mg) and triethylamine (1 ml) was added to the solution and heated at 150 ℃ for 24 h under N_2_. After cooling to room temperature, 2-ethoxyethanol was distilled under the low pressure. The crude product was purified by silica gel column (eluent = dichloromethane/petroleum ether 3:1 v/v) to afford the expected product (376.2 mg, 47%).^1^H NMR (400 MHz, Chloroform-*d*) δ 8.75 (s, 1H), 8.68 (dd, *J* = 8.8, 5.4 Hz, 1H), 8.01 (d, *J* = 1.8 Hz, 1H), 7.47 (d, *J* = 2.1 Hz, 2H), 7.25 – 7.22 (m, 3H), 6.72 (d, *J* = 2.1 Hz, 2H), 6.55 (td, *J* = 8.6, 2.9 Hz, 1H), 5.59 (dd, *J* = 10.1, 2.8 Hz, 1H), 4.86 (s, 3H), 3.51 – 3.36 (m, 4H), 1.36 – 1.24 (m, 2H), 1.02 – 0.91 (m, 2H), 0.83 – 0.73 (m, 2H), 0.49 – 0.44 (m, 8H).; ^19^F NMR (376 MHz, Chloroform-*d*) δ -60.25, -117.38; ESI-MS calcd for C_35_H_40_IIrN_7_ 878.2019, found 878.2008.

**[(pbib)Ir(CF_3_pmpF)CN] (CF_3_-3)**:

Under the protection of N_2_, [(pbib)Ir(CF_3_pmpF)I] (0.34 mmol, 299.5 mg) and the double molar quantity of AgCN (0.68 mmol, 91.6 mg) were dissolved in a round-bottom flask containing 20 mL of *N*,*N*-dimethylformamide (DMF). The suspension was heated at 100 °C under N_2_ for 2 h. After cooling, the solvent was filtered and removed off by rotary evaporation to afford the crude product. The crude product was purified by silica gel column (eluent = dichloromethane/ethyl acetate 3:1 v/v) to afford the expected product. Then, the expected product was recrystallized in CH_2_Cl_2_/diethyl ether (273.2 mg, 95%).^1^H NMR (400 MHz, Chloroform-*d*) δ 8.75 (s, 1H), 8.68 (dd, *J* = 8.7, 5.0 Hz, 1H), 8.04 (d, *J* = 1.9 Hz, 1H), 7.48 (d, *J* = 2.1 Hz, 2H), 7.32 (dd, *J* = 8.5, 7.0 Hz, 1H), 7.23 – 7.21 (m, 2H), 6.74 (d, *J* = 2.1 Hz, 2H), 6.57 (td, *J* = 8.7, 2.9 Hz, 1H), 5.65 (dd, *J* = 9.5, 2.9 Hz, 1H), 4.73 (s, 3H), 3.37 – 3.30 (m, 4H), 1.36 – 1.25 (m, 2H), 1.02 – 0.91 (m, 2H), 0.83 – 0.72 (m, 2H), 0.51 – 0.45 (m, 8H).; ^19^F NMR (376 MHz, Chloroform-*d*) δ -60.26, -117.98; ^13^C NMR (125 MHz, Chloroform-*d*) δ 191.62, 163.72, 146.85, 145.64, 145.25 (2C), 143.34, 136.16, 136.06, 132.41, 129.60, 129.01 (2C), 128.81, 128.74, 128.26, 122.89, 119.06 (2C), 117.70, 117.48, 116.20, 115.85 (2C), 107.65 (2C), 52.56, 50.86 (2C), 33.03, 19.81 (2C), 19.79, 19.76, 13.44 (2C). ESI-MS calcd for C_35_H_33_F_4_IrN_8_ 835.2467; Found: 835.2466.

**[(pbib)Ir(FpmpMe_2_)I]**:

Under the protection of N_2_, **pbib** (0.90 mmol, 435.9 mg) and [Ir(COD)Cl]_2_ (0.45 mmol, 300.0 mg) were added to a mixture of triethylamine (1 mL) and acetonitrile (10 mL) in Schlenk tube. The suspension was heated at 90 °C for 12 h. After cooling, the solvent was distilled by rotary evaporation to give yellow solid. Then the yellow solid was dissolved in 2-ethoxyethanol (10 mL), and **FpmpMe_2_** (0.90 mmol, 344.7 mg) and triethylamine (1 ml) was added to the solution and heated at 150℃ for 24 h under N_2_. After cooling to room temperature, 2-ethoxyethanol was distilled under the low pressure. The crude product was purified by silica gel column (eluent = dichloromethane/petroleum ether 3:1 v/v) to afford the expected product (322.8 mg, 49%).^1^H NMR (400 MHz, Chloroform-*d*) δ 8.42 (s, 1H), 8.35 (dd, *J* = 2.4, 1.6 Hz, 1H), 7.53 (dd, *J* = 8.1, 2.7 Hz, 1H), 7.43 (d, *J* = 2.0 Hz, 2H), 7.23 – 7.19 (m, 3H), 6.68 (d, *J* = 2.0 Hz, 2H), 5.58 (s, 1H), 4.78 (s, 3H), 3.50 – 3.34 (m, 4H), 2.13 (s, 3H), 1.82 (s, 3H), 1.43 – 1.34 (m, 2H), 1.11 – 1.00 (m, 2H), 0.88 – 0.75 (m, 2H), 0.60 – 0.52 (m, 8H).; ^19^F NMR (376 MHz, Chloroform-*d*) δ -135.06

**[(pbib)Ir(FpmpMe_2_)CN] (F-1)**:

Under the protection of N_2_, [(pbib)Ir(FpmpMe_2_)I] (0.34 mmol, 285.9 mg) and the double molar quantity of AgCN (0.68 mmol, 91.6 mg) were dissolved in a round-bottom flask containing 20 mL of *N*,*N*-dimethylformamide (DMF). The suspension was heated at 100 °C under N_2_ for 2 h. After cooling, the solvent was filtered and removed off by rotary evaporation to afford the crude product. The crude product was purified by silica gel column (eluent = dichloromethane/ethyl acetate 3:1 v/v) to afford the expected product. Then, the expected product was recrystallized in CH_2_Cl_2_/diethyl ether (263.7 mg, 95%).

^1^H NMR (400 MHz, Chloroform-*d*) δ 8.44 (s, 1H), 8.37 (dd, *J* = 2.5, 1.5 Hz, 1H), 7.57 (dd, *J* = 7.8, 2.5 Hz, 1H), 7.44 (d, *J* = 2.1 Hz, 2H), 7.29 (dd, *J* = 8.5, 6.9 Hz, 1H), 7.21 – 7.19 (m, 2H), 6.70 (d, *J* = 2.1 Hz, 2H), 5.63 (s, 1H), 4.63 (s, 3H), 3.38 – 3.25 (m, 4H), 2.13 (s, 3H), 1.80 (s, 3H), 1.45 – 1.34 (m, 2H), 1.12 – 1.01 (m, 2H), 0.89 – 0.76 (m, 2H), 0.63 – 0.53 (m, 8H).; ^19^F NMR (376 MHz, Chloroform-*d*) δ -134.68; ^13^C NMR (125 MHz, Chloroform-*d*) δ 191.62, 163.72, 146.85, 145.64, 145.25 (2C), 143.34, 136.16, 136.06, 132.41, 129.60, 129.01 (2C), 128.81, 128.74, 128.26, 122.89, 119.06 (2C), 117.70, 117.48, 116.20, 115.85 (2C), 107.65 (2C), 52.56, 50.86 (2C), 33.03, 19.81 (2C), 19.79, 19.76, 13.44 (2C). ESI-MS calcd for C_36_H_38_F_1_IrN_8_ 795.2906; Found: 795.2906.

**[(pbib)Ir(Fpmp)I]**:

Under the protection of N_2_, **pbib** (0.90 mmol, 435.9 mg) and [Ir(COD)Cl]_2_ (0.45 mmol, 300.0 mg) were added to a mixture of triethylamine (1 mL) and acetonitrile (10 mL) in Schlenk tube. The suspension was heated at 90 °C for 12 h. After cooling, the solvent was distilled by rotary evaporation to give yellow solid. Then the yellow solid was dissolved in 2-ethoxyethanol (10 mL), and **Fpmp** (0.90 mmol, 319.5 mg) and triethylamine (1 ml) was added to the solution and heated at 150 ℃ for 24 h under N_2_. After cooling to room temperature, 2-ethoxyethanol was distilled under the low pressure. The crude product was purified by silica gel column (eluent = dichloromethane/petroleum ether 3:1 v/v) to afford the expected product (298.4 mg, 47%).^1^H NMR (400 MHz, Chloroform-*d*) δ 8.64 (dd, *J* = 7.9, 1.1 Hz, 1H), 8.36 – 8.35 (m, 1H), 7.56 (dd, *J* = 7.8, 2.5 Hz, 1H), 7.43 (d, *J* = 2.1 Hz, 2H), 7.24 – 7.20 (m, 3H), 6.86 – 6.81 (m, 1H), 6.68 (d, *J* = 2.1 Hz, 2H), 6.46 (td, *J* = 7.5, 1.5 Hz, 1H), 5.90 (dd, *J* = 7.7, 1.4 Hz, 1H), 4.79 (s, 3H), 3.50 – 3.35 (m, 4H), 1.39 – 1.29 (m, 2H), 1.05 – 0.94 (m, 2H), 0.84 – 0.72 (m, 2H), 0.53 – 0.45 (m, 8H).; ^19^F NMR (376 MHz, Chloroform-*d*) δ -134.56.

**[(pbib)Ir(Fpmp)CN](F-2)**:

Under the protection of N_2_, [(pbib)Ir(Fpmp)I] (0.34 mmol, 276.4 mg) and the double molar quantity of AgCN (0.68 mmol, 91.6 mg) were dissolved in a round-bottom flask containing 20 mL of *N*,*N*-dimethylformamide (DMF). The suspension was heated at 100 °C under N_2_ for 2 h. After cooling, the solvent was filtered and removed off by rotary evaporation to afford the crude product. The crude product was purified by silica gel column (eluent = dichloromethane/ethyl acetate 3:1 v/v) to afford the expected product. Then, the expected product was recrystallized in CH_2_Cl_2_/diethyl ether (264.8 mg, 95%).^1^H NMR (400 MHz, Chloroform-*d*) δ 8.66 (dd, *J* = 8.0, 1.2 Hz, 1H), 8.37 (dd, *J* = 2.5, 1.4 Hz, 1H), 7.61 (dd, *J* = 7.8, 2.5 Hz, 1H), 7.46 (d, *J* = 2.1 Hz, 2H), 7.32 (dd, *J* = 8.5, 7.0 Hz, 1H), 7.23 – 7.21 (m, 2H), 6.89 (td, *J* = 8.0, 1.5 Hz, 1H), 6.72 (d, *J* = 2.1 Hz, 2H), 6.50 (td, *J* = 7.3, 1.3 Hz, 1H), 5.94 (dd, *J* = 7.4, 1.5 Hz, 1H), 4.60 (s, 3H), 3.38 – 3.28 (m, 4H), 1.38 – 1.24 (m, 2H), 1.05 – 0.94 (m, 2H), 0.85 – 0.74 (m, 2H), 0.55 – 0.44 (m, 8H).; ^19^F NMR (376 MHz, Chloroform-*d*) δ -133.97; ^13^C NMR (125 MHz, Chloroform-*d*) δ 191.62, 163.72, 146.85, 145.64, 145.25 (2C), 143.34, 136.16, 136.06, 132.41, 129.60, 129.01 (2C), 128.81, 128.74, 128.26, 122.89, 119.06 (2C), 117.70, 117.48, 116.20, 115.85 (2C), 107.65 (2C), 52.56, 50.86 (2C), 33.03, 19.81 (2C), 19.79, 19.76, 13.44 (2C). ESI-MS calcd for C_34_H_34_FIrN_8_ 767.2593; Found: 767.2597.

NMR Spectrum


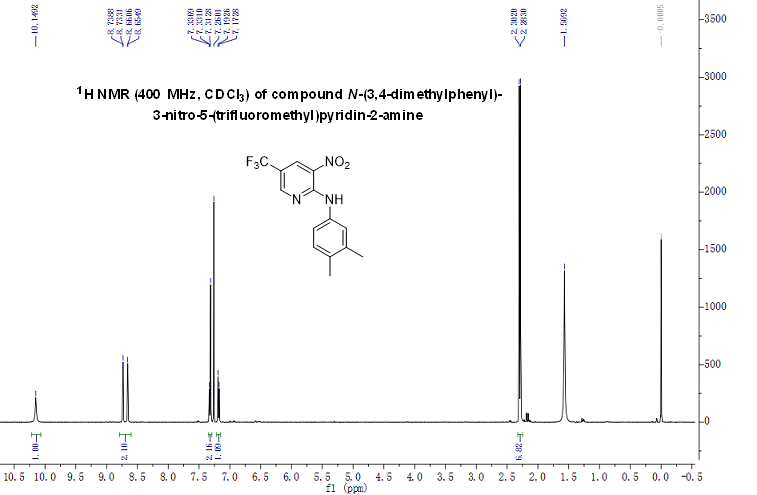


**Figure S2.** ^1^H NMR (400 MHz, CDCl_3_) of compound N-(3,4-dimethylphenyl)-3- nitro-5-(trifluoromethyl)pyridin-2-amine


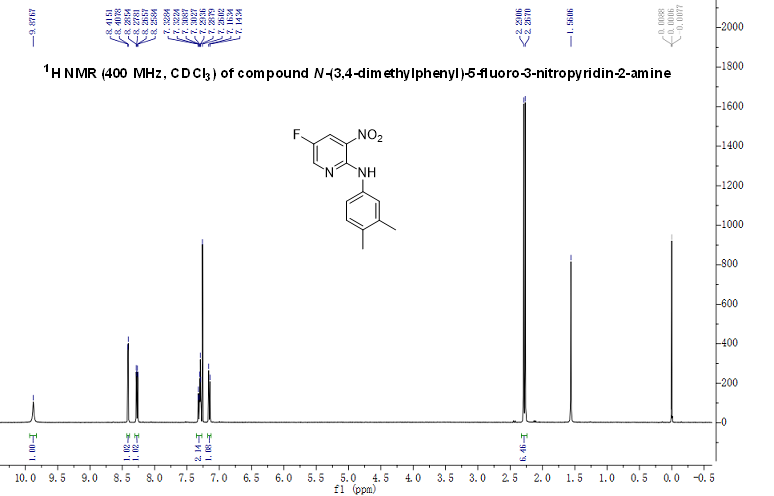


**Figure S3.** ^1^H NMR (400 MHz, CDCl_3_) of compound N-(3,4-dimethylphenyl)-5- fluoro-3-nitropyridin-2-amine


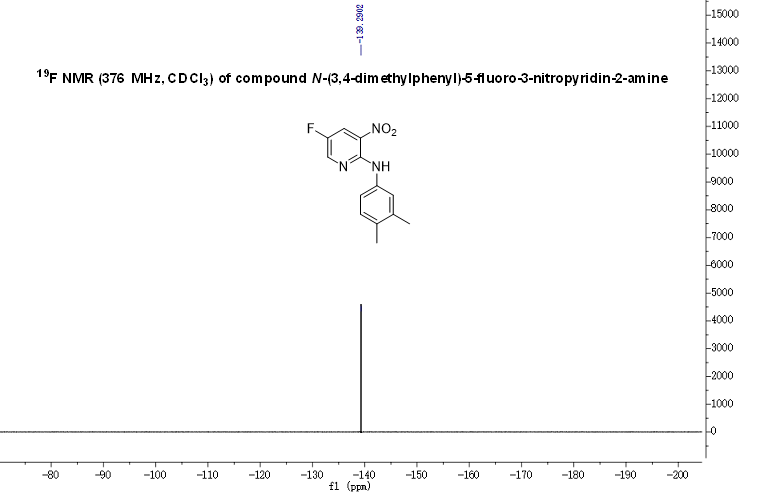


**Figure S4.** ^13^F NMR (376 MHz, CDCl_3_) of compound N-(3,4-dimethylphenyl)-5- fluoro-3-nitropyridin-2-amine


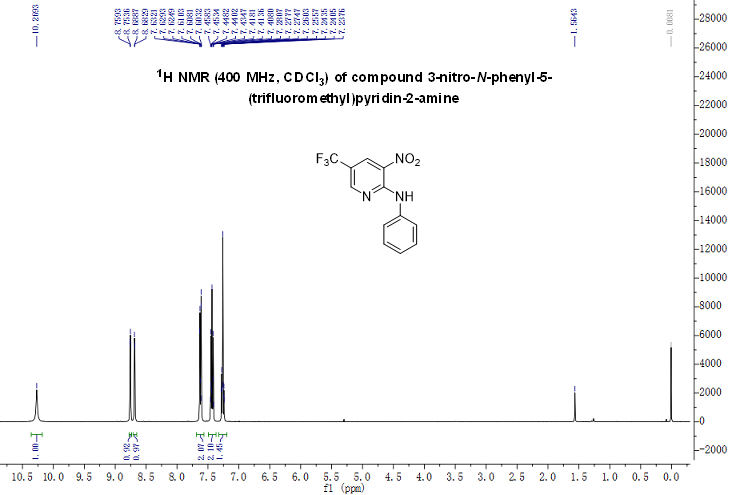


**Figure S5.** ^1^H NMR (400 MHz, CDCl_3_) of compound 3-nitro-N-phenyl-5- (trifluoromethyl)pyridin-2-amine


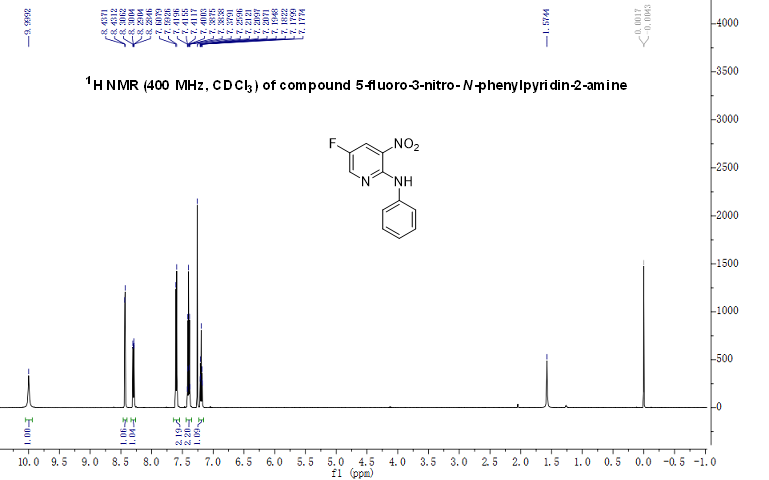


**Figure S6.** ^1^H NMR (400 MHz, CDCl_3_) of compound 5-fluoro-3-nitro- N-phenylpyridin-2-amine


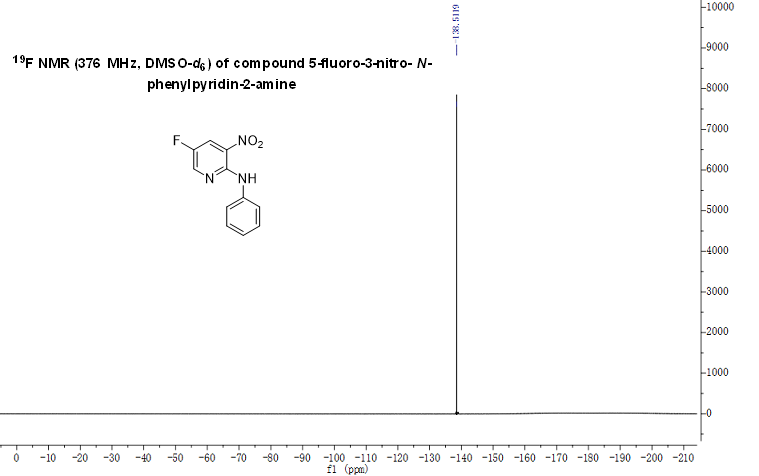


**Figure S7.** ^13^F NMR (376 MHz, CDCl_3_) of compound 5-fluoro-3-nitro- N-phenylpyridin-2-amine


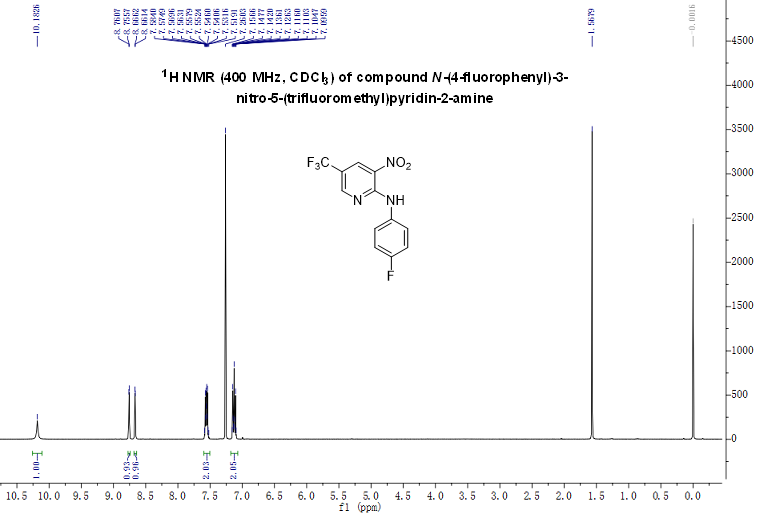


**Figure S8.** ^1^H NMR (400 MHz, CDCl_3_) of compound N-(4-fluorophenyl)-3-nitro-5- (trifluoromethyl)pyridin-2-amine


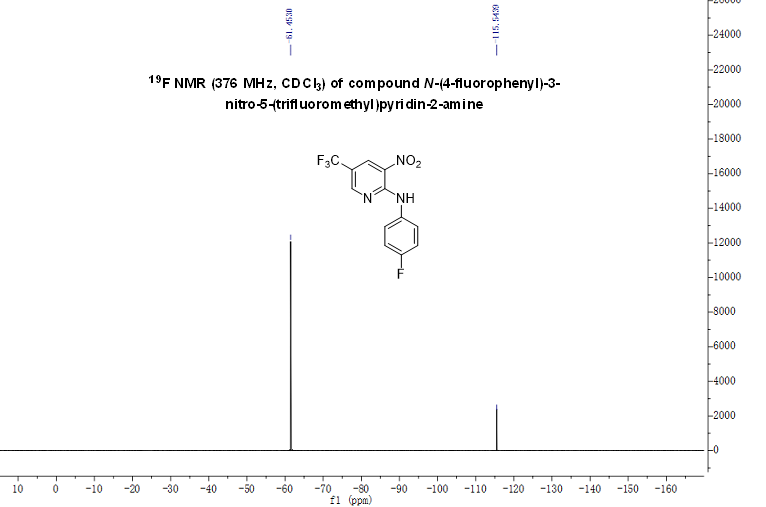


**Figure S9.** ^13^F NMR (376 MHz, CDCl_3_) of compound N-(4-fluorophenyl)-3-nitro-5- (trifluoromethyl)pyridin-2-amine


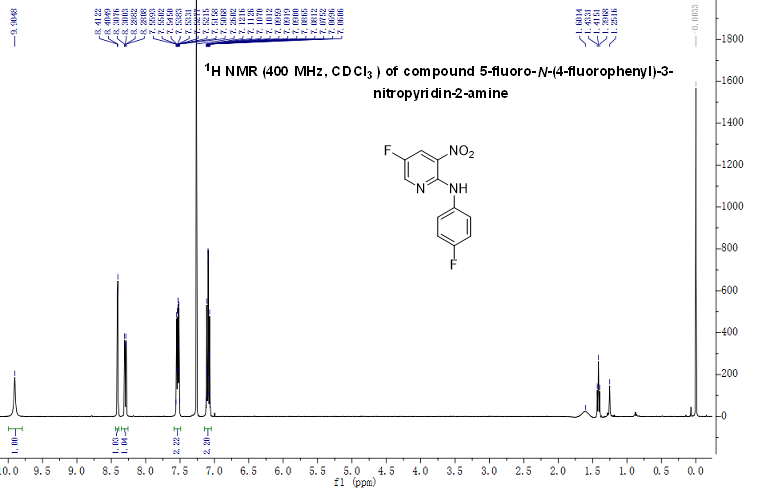


**Figure S10.** ^1^H NMR (400 MHz, CDCl_3_) of compound 5-fluoro-N-(4-fluorophenyl) -3-nitropyridin-2-amine


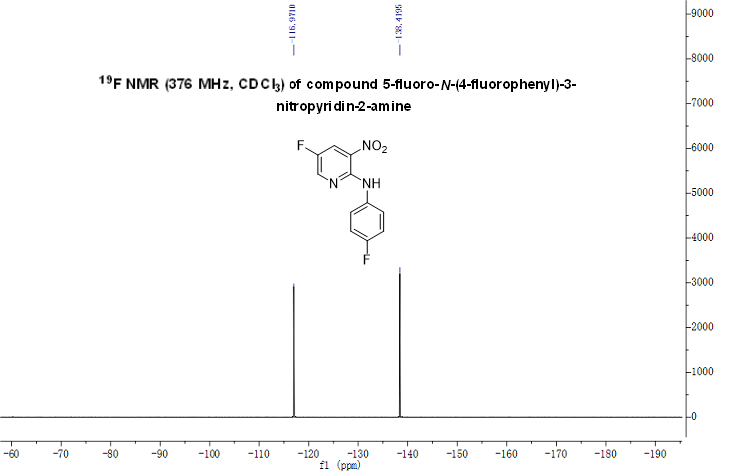


**Figure S11.** ^13^F NMR (376 MHz, CDCl_3_) of compound 5-fluoro-N-(4-fluorophenyl) -3-nitropyridin-2-amine


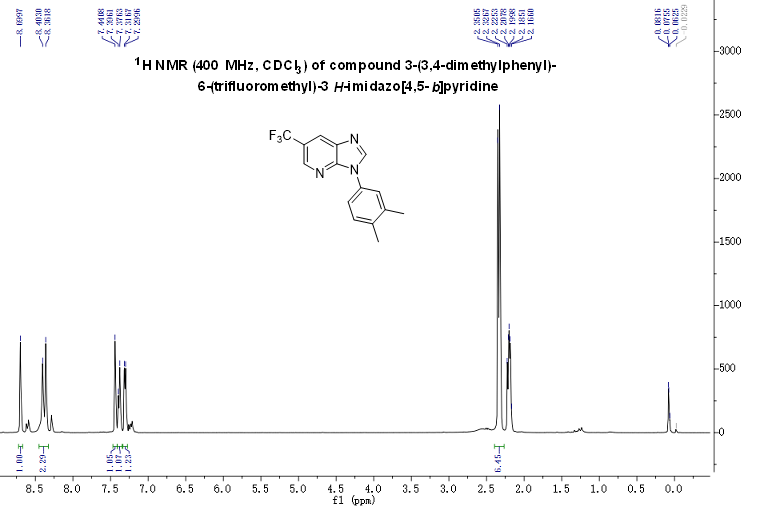


**Figure S12.** ^1^H NMR (400 MHz, CDCl_3_) of compound 3-(3,4-dimethylphenyl)-6- (trifluoromethyl)-3H-imidazo[4,5-b]pyridine


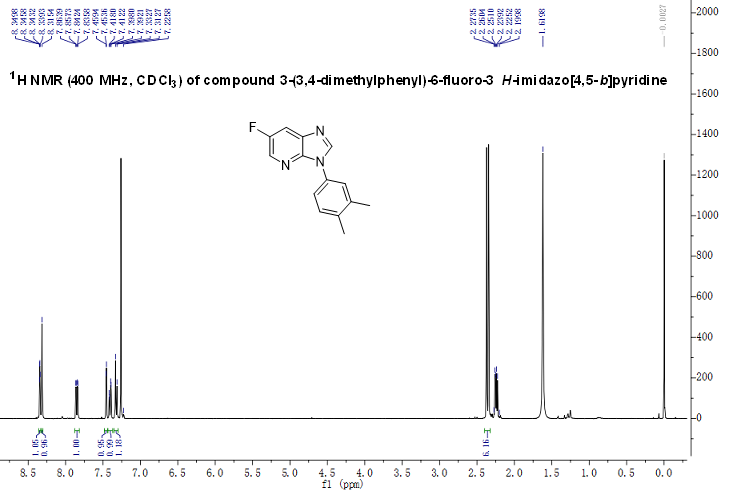


**Figure S13.** ^1^H NMR (400 MHz, CDCl_3_) of compound 3-(3,4-dimethylphenyl)-6- fluoro-3H-imidazo[4,5-b]pyridine


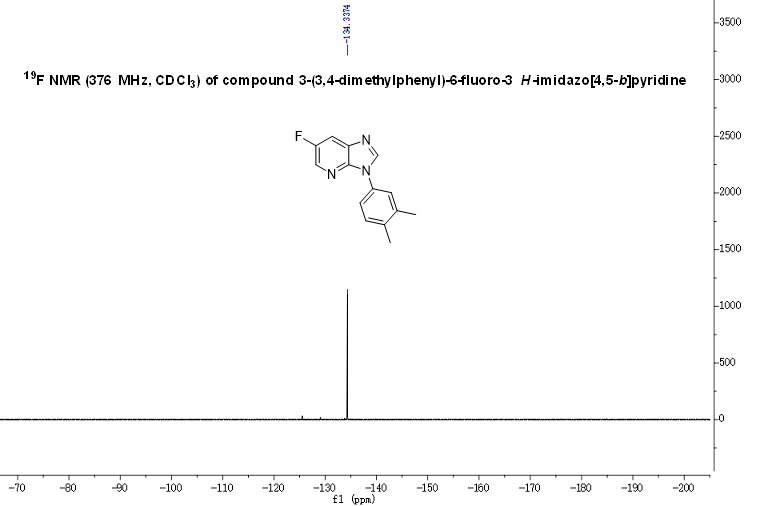


**Figure S14.** ^13^F NMR (376 MHz, CDCl_3_) of compound 3-(3,4-dimethylphenyl)-6- fluoro-3H-imidazo[4,5-b]pyridine


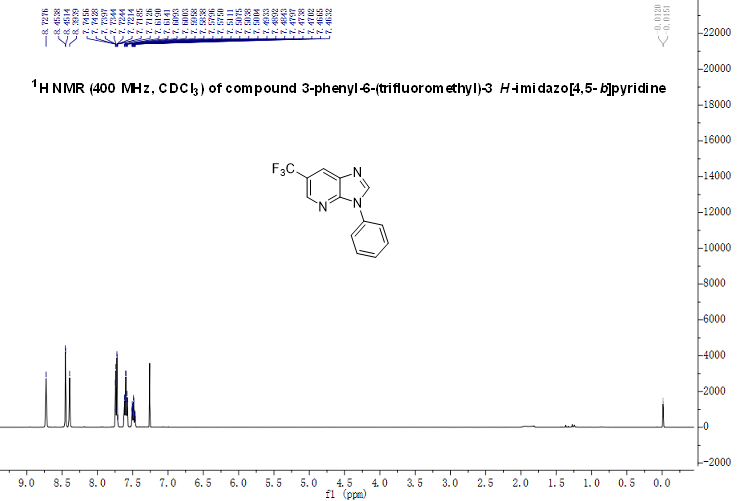


**Figure S15.** ^1^H NMR (400 MHz, CDCl_3_) of compound 3-phenyl-6-(trifluoromethyl)- 3H-imidazo[4,5-b]pyridine


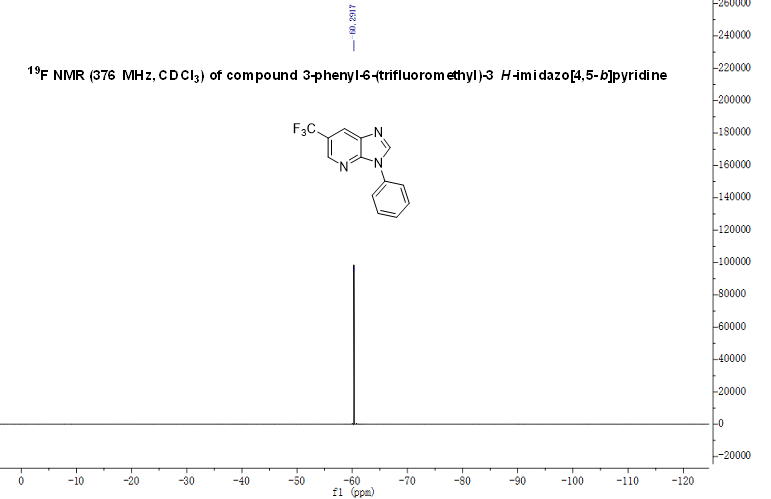


**Figure S16.** ^13^F NMR (376 MHz, CDCl_3_) of compound 3-phenyl-6-(trifluoromethyl)- 3H-imidazo[4,5-b]pyridine


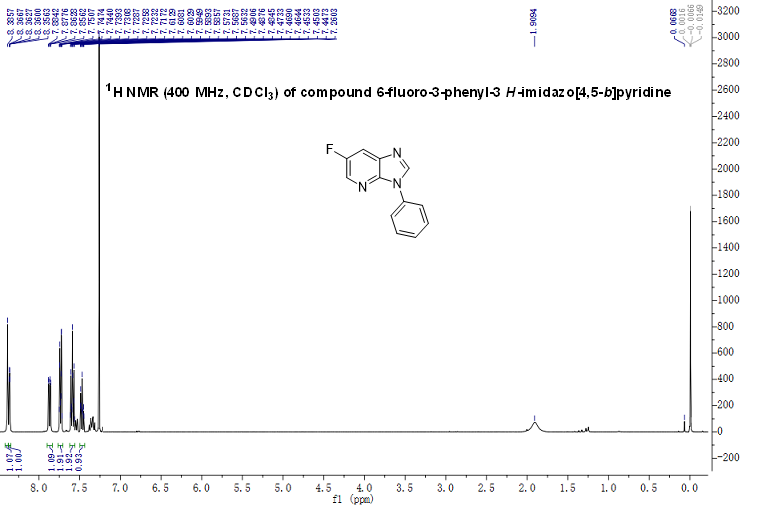


**Figure S17.** ^1^H NMR (400 MHz, CDCl_3_) of compound 6-fluoro-3-phenyl- 3H-imidazo[4,5-b]pyridine


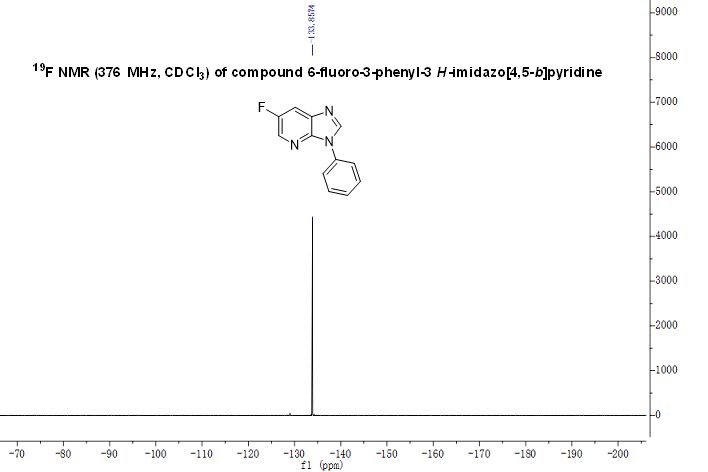


**Figure S18.** ^13^F NMR (376 MHz, CDCl_3_) of compound 6-fluoro-3-phenyl- 3H-imidazo[4,5-b]pyridine


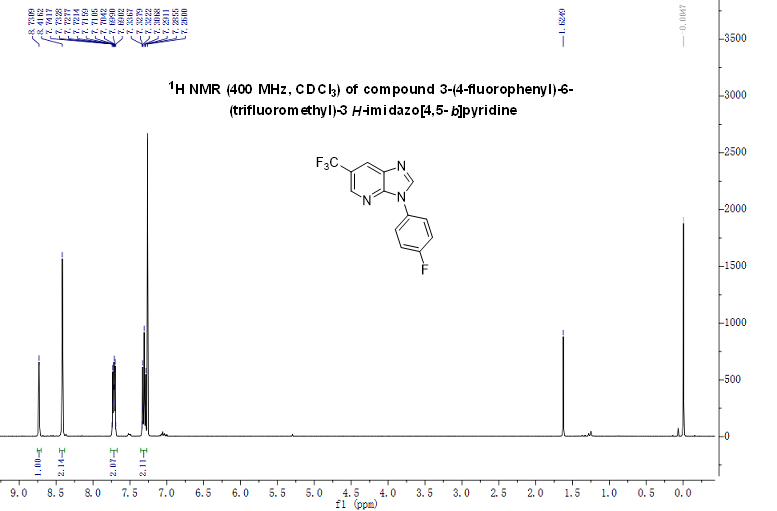


**Figure S19.** ^1^H NMR (400 MHz, CDCl_3_) of compound 3-(4-fluorophenyl)-6- (trifluoromethyl)-3H-imidazo[4,5-b]pyridine


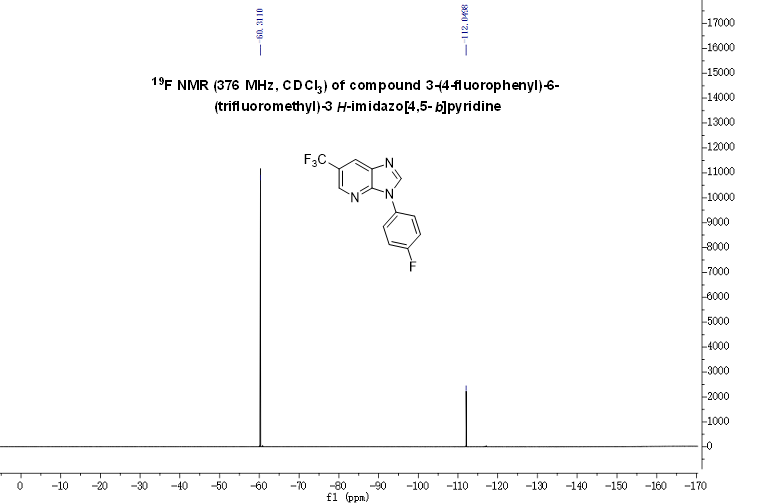


**Figure S20.** ^13^F NMR (376 MHz, CDCl_3_) of compound 3-(4-fluorophenyl)-6- (trifluoromethyl)-3H-imidazo[4,5-b]pyridine


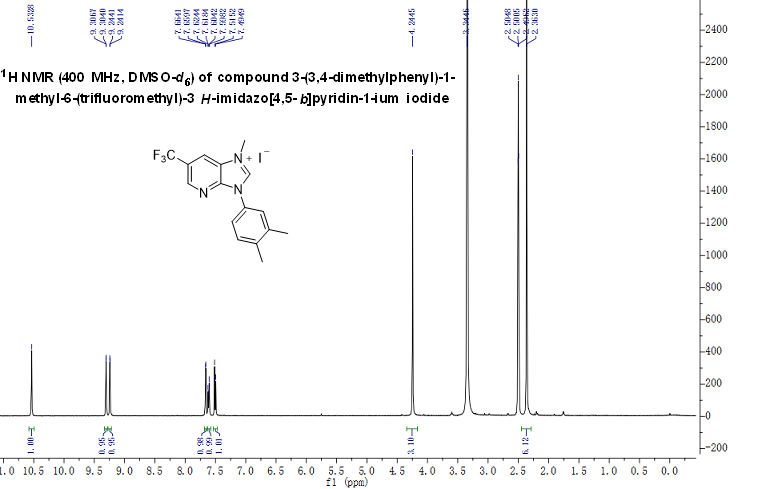


**Figure S21.** ^1^H NMR (400 MHz, CDCl_3_) of compound 3-(3,4-dimethylphenyl)-1-methyl-6-(trifluoromethyl)-3H-imidazo[4,5-b]pyridin-1-ium iodide (CF_3_pmpMe_2_)


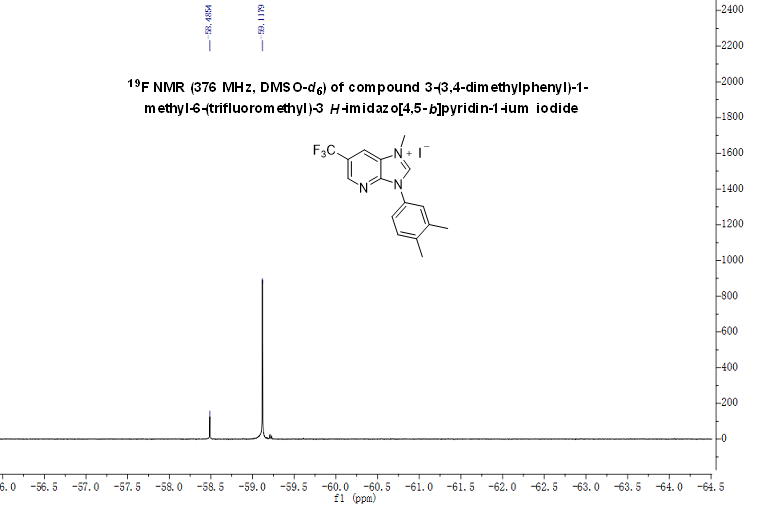


**Figure S22.** ^13^F NMR (376 MHz, CDCl_3_) of compound 3-(3,4-dimethylphenyl)-1-methyl-6-(trifluoromethyl)-3H-imidazo[4,5-b]pyridin-1-iumiodide (CF_3_pmpMe_2_)


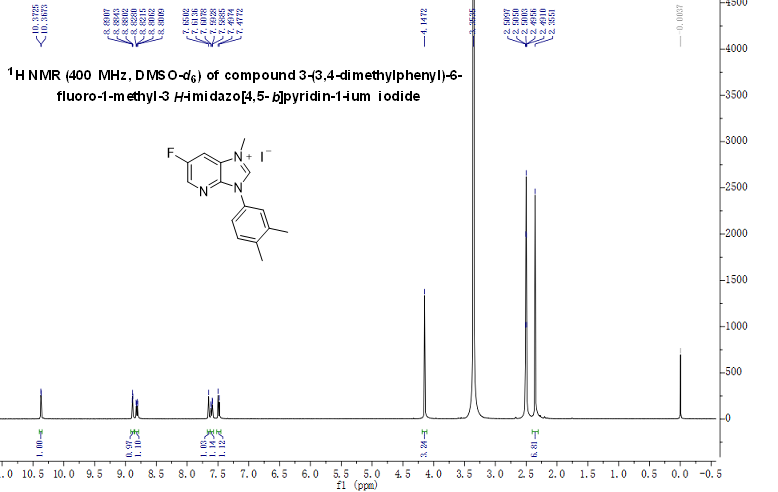


**Figure S23.** ^1^H NMR (400 MHz, CDCl_3_) of compound 3-(3,4-dimethylphenyl)-6-fluoro-1-methyl-3H-imidazo[4,5-b]pyridin-1-iumiodide (FpmpMe_2_)


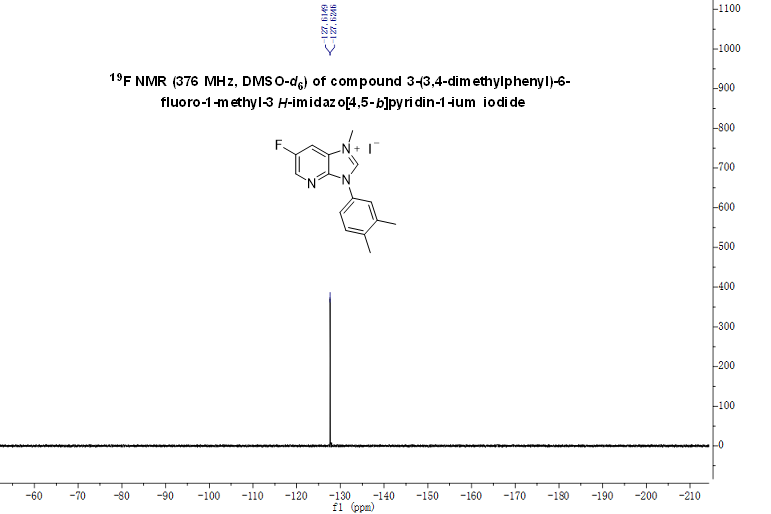


**Figure S24.** ^13^F NMR (376 MHz, CDCl_3_) of compound 3-(3,4-dimethylphenyl)-6-fluoro-1-methyl-3H-imidazo[4,5-b]pyridin-1-iumiodide (FpmpMe_2_)


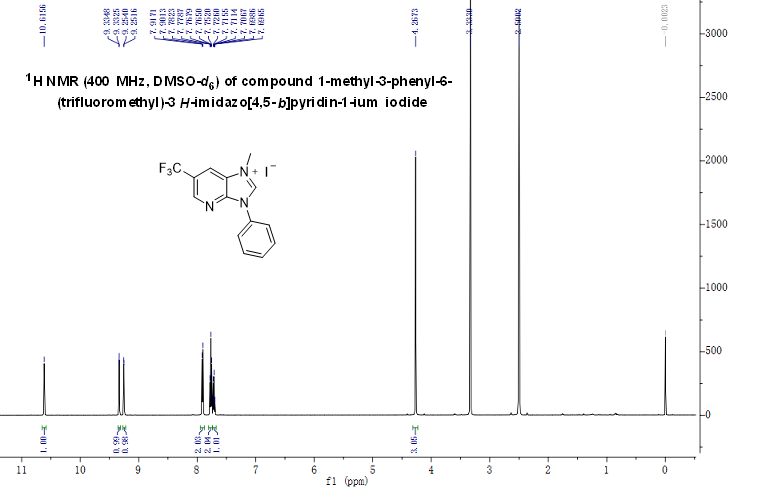


**Figure S25.** ^1^H NMR (400 MHz, CDCl_3_) of compound 1-methyl-3-phenyl-6- (trifluoromethyl)-3H-imidazo[4,5-b]pyridin-1-ium iodide (CF_3_pmp)


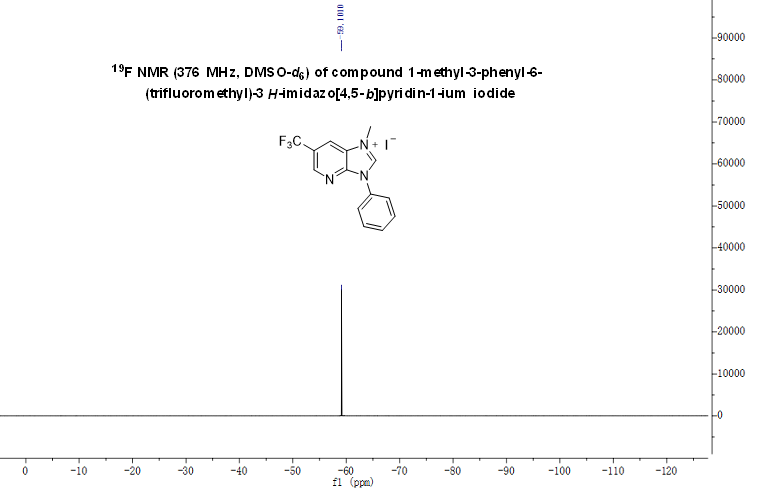


**Figure S26.** ^13^F NMR (376 MHz, CDCl_3_) of compound 1-methyl-3-phenyl-6- (trifluoromethyl)-3H-imidazo[4,5-b]pyridin-1-ium iodide (CF_3_pmp)


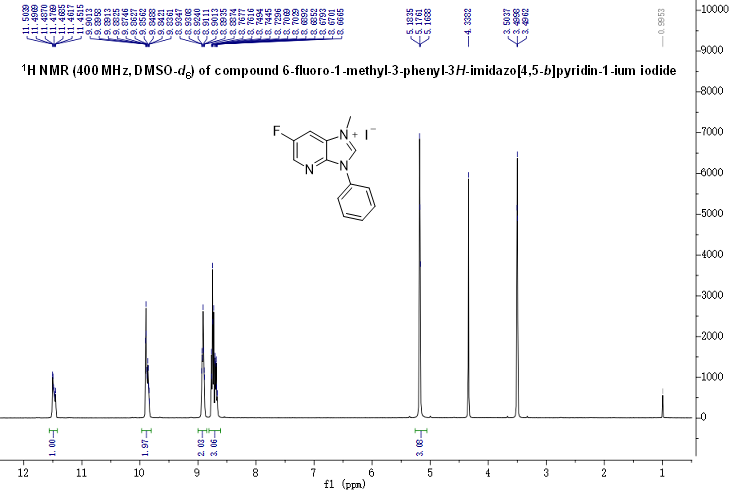


**Figure S27.** ^1^H NMR (400 MHz, CDCl_3_) of compound 6-fluoro-1-methyl-3-phenyl- 3H-imidazo[4,5-b]pyridin-1-ium iodide (Fpmp)


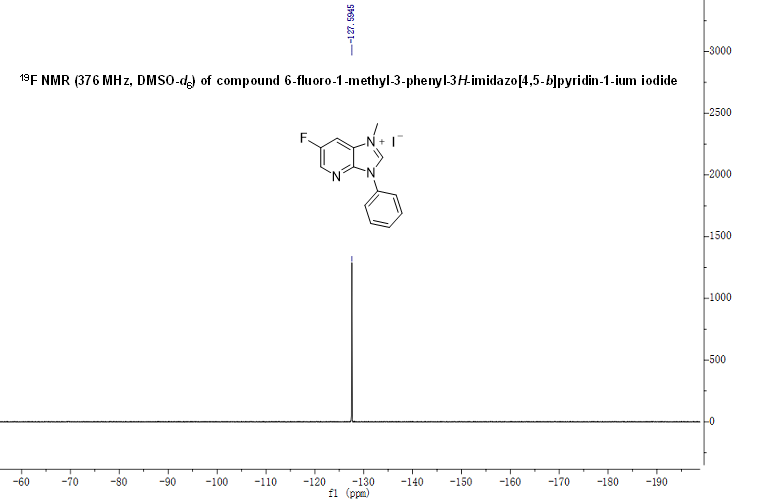


**Figure S28.** ^13^F NMR (376 MHz, CDCl_3_) of compound 6-fluoro-1-methyl-3-phenyl- 3H-imidazo[4,5-b]pyridin-1-ium iodide (Fpmp)


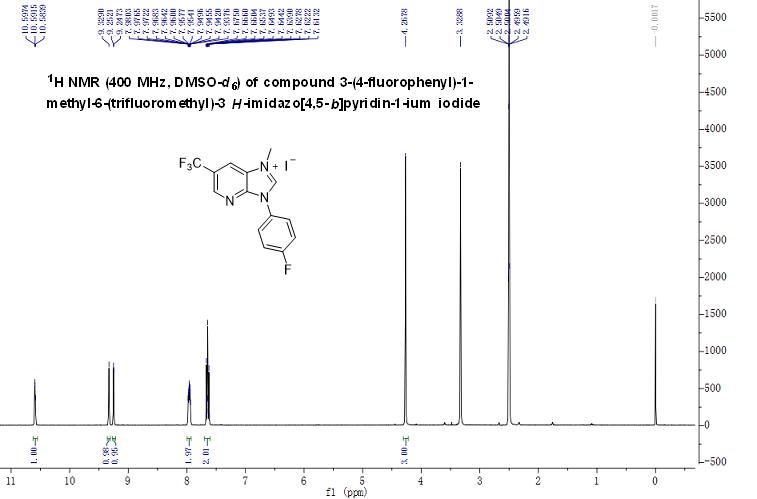


**Figure S29.** ^1^H NMR (400 MHz, CDCl_3_) of compound 3-(4-fluorophenyl)-1-methyl- 6-(trifluoromethyl)-3H-imidazo[4,5-b]pyridin-1-ium iodide (CF_3_pmpF)


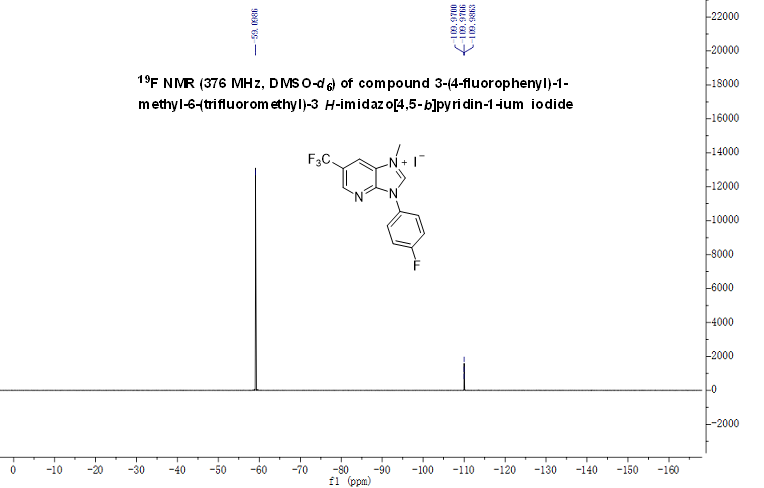


**Figure S30.** ^13^F NMR (376 MHz, CDCl_3_) of compound 3-(4-fluorophenyl)-1-methyl- 6-(trifluoromethyl)-3H-imidazo[4,5-b]pyridin-1-ium iodide (CF_3_pmpF)


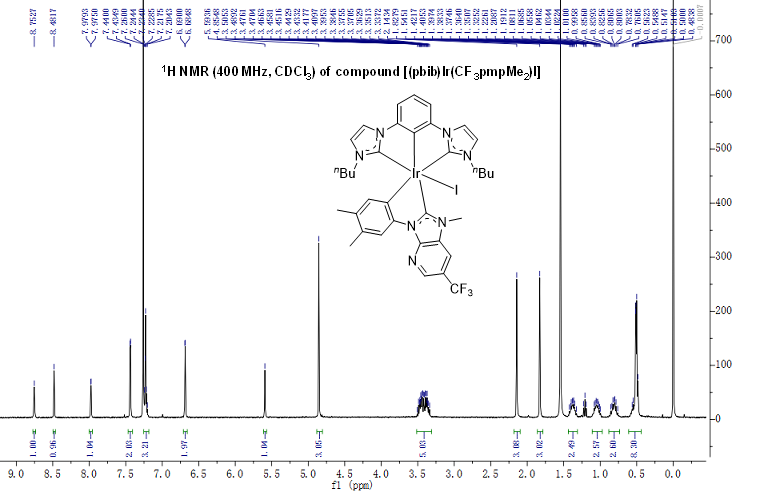


**Figure S31.** ^1^H NMR (400 MHz, CDCl_3_) of compound [(pbib)Ir(CF_3_pmpMe_2_)I]


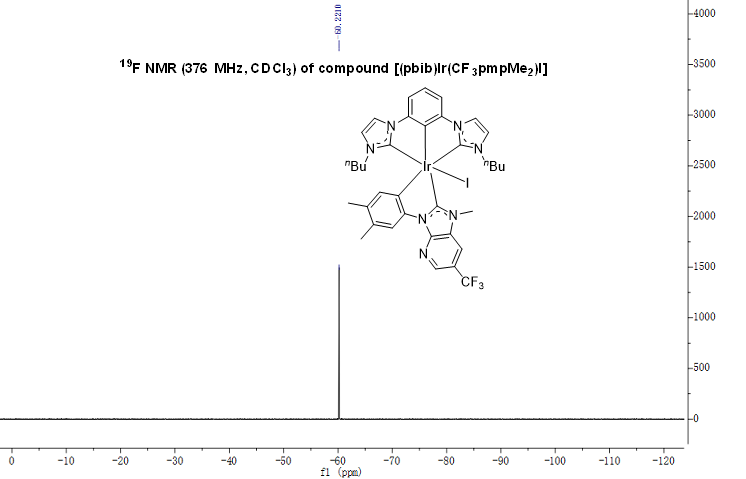


**Figure S32.** ^13^F NMR (376 MHz, CDCl_3_) of compound [(pbib)Ir(CF_3_pmpMe_2_)I]


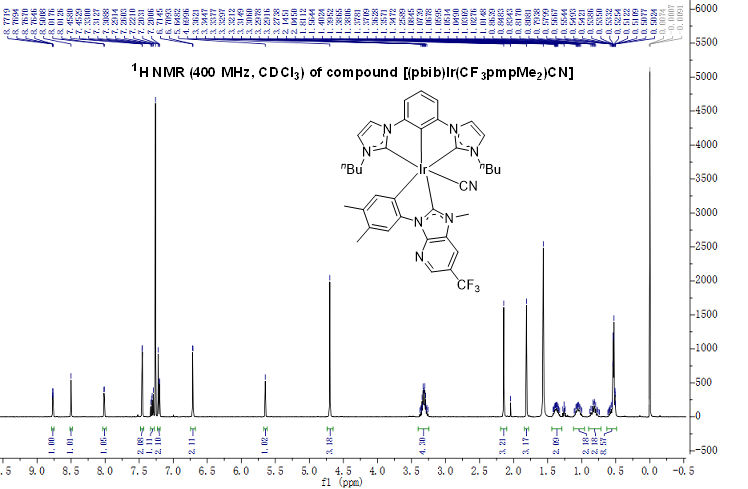


**Figure S33.** ^1^H NMR (400 MHz, CDCl_3_) of compound [(pbib)Ir(CF_3_pmpMe_2_)CN] (**CF_3_-1**)


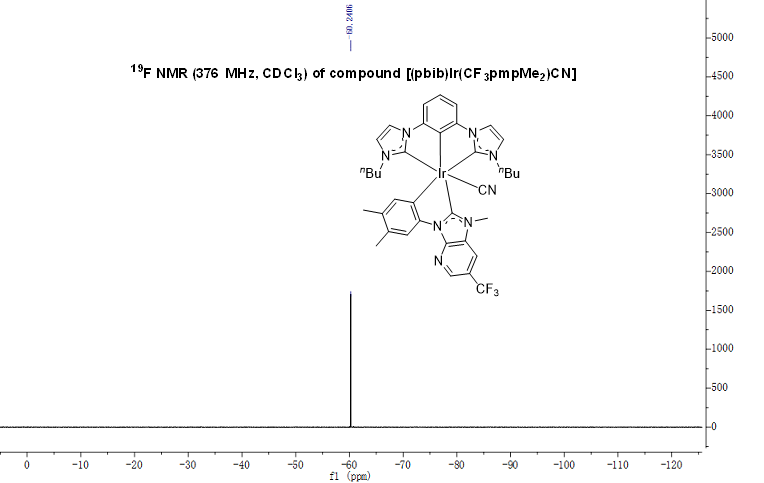


**Figure S34.** ^13^F NMR (376 MHz, CDCl_3_) of compound [(pbib)Ir(CF_3_pmpMe_2_)CN](**CF_3_-1**)


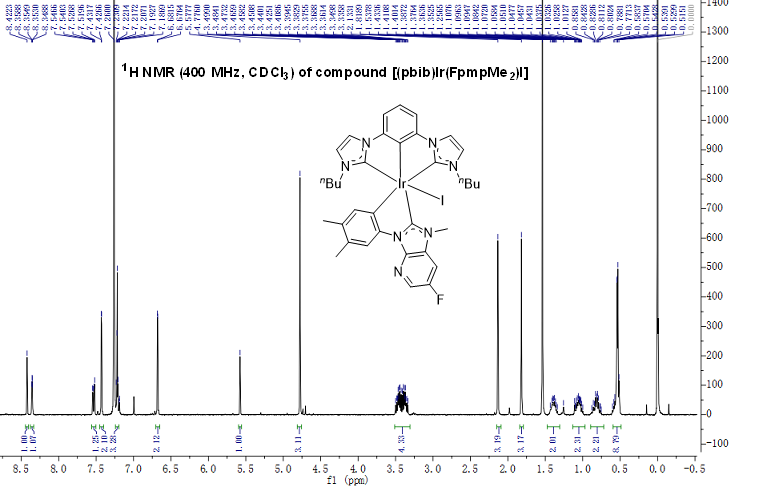


**Figure S35.** ^1^H NMR (400 MHz, CDCl_3_) of compound [(pbib)Ir(FpmpMe_2_)I]


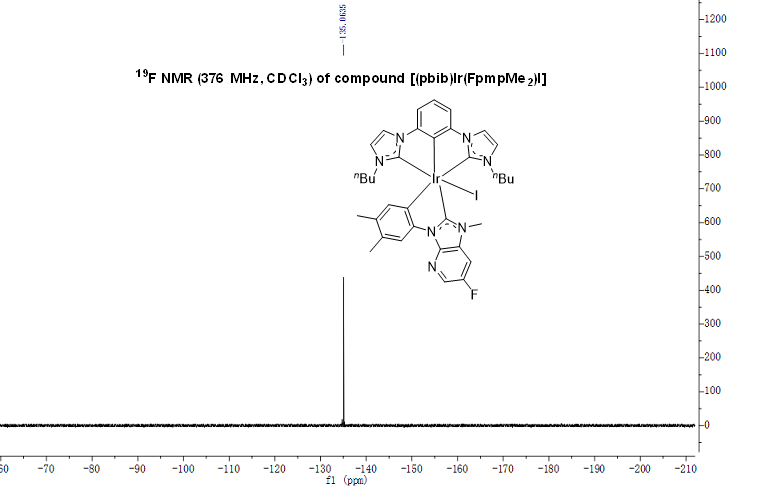


**Figure S36.** ^13^F NMR (376 MHz, CDCl_3_) of compound [(pbib)Ir(FpmpMe_2_)I]


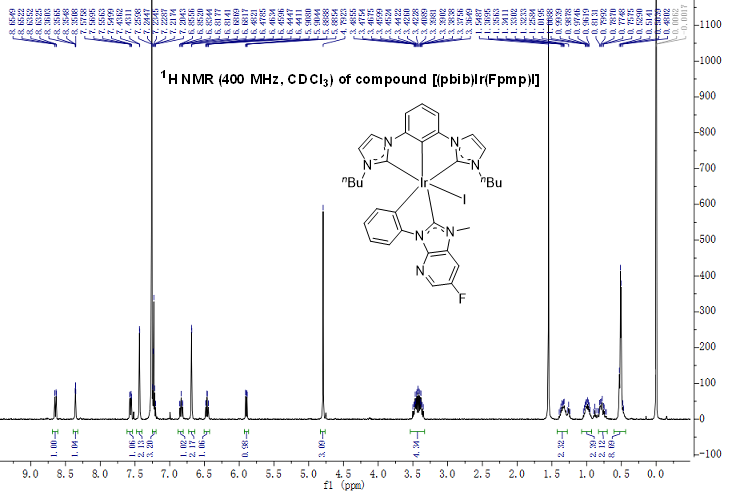


**Figure S37.** ^1^H NMR (400 MHz, CDCl_3_) of compound [(pbib)Ir(Fpmp)I]


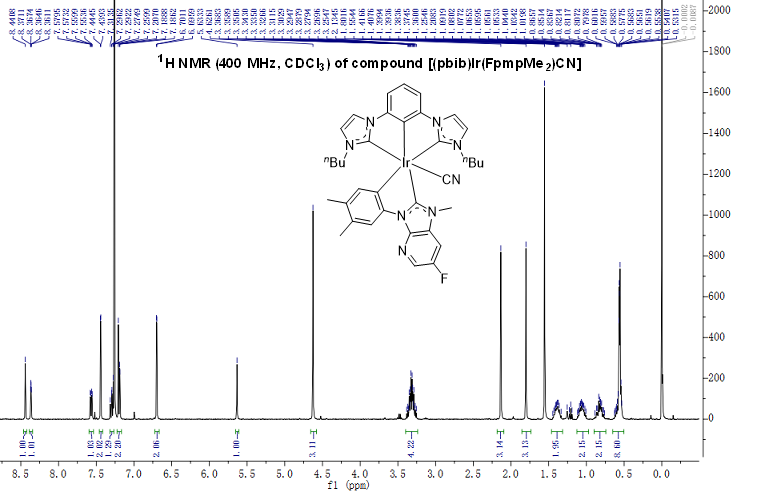


**Figure S38.** ^1^H NMR (400 MHz, CDCl_3_) of compound [(pbib)Ir(FpmpMe_2_)CN] (**F-1**)


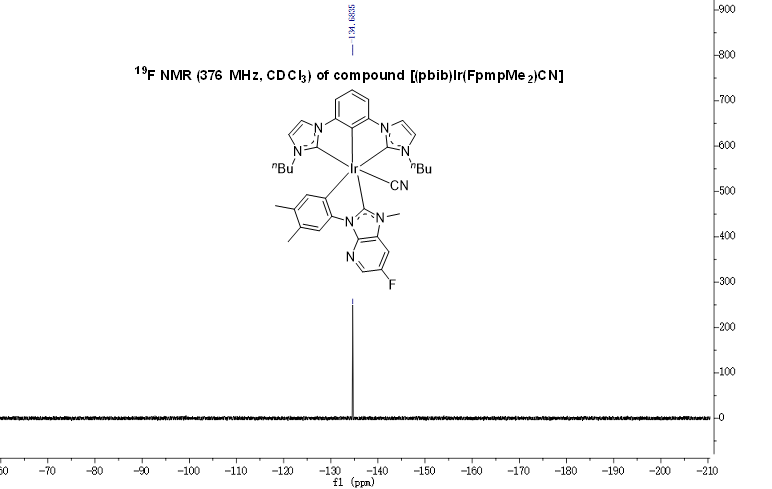


**Figure S39.** ^13^F NMR (376 MHz, CDCl_3_) of compound [(pbib)Ir(FpmpMe_2_)CN] (**F-1**)


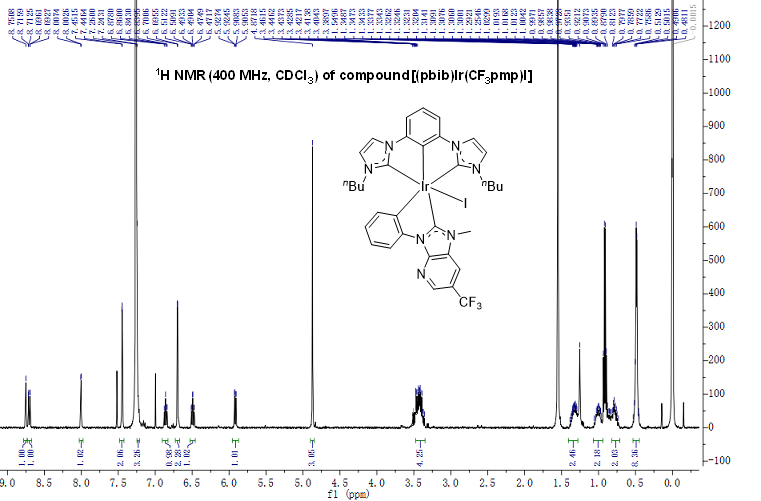


**Figure S40.** ^1^H NMR (400 MHz, CDCl_3_) of compound [(pbib)Ir(CF_3_pmp)I]


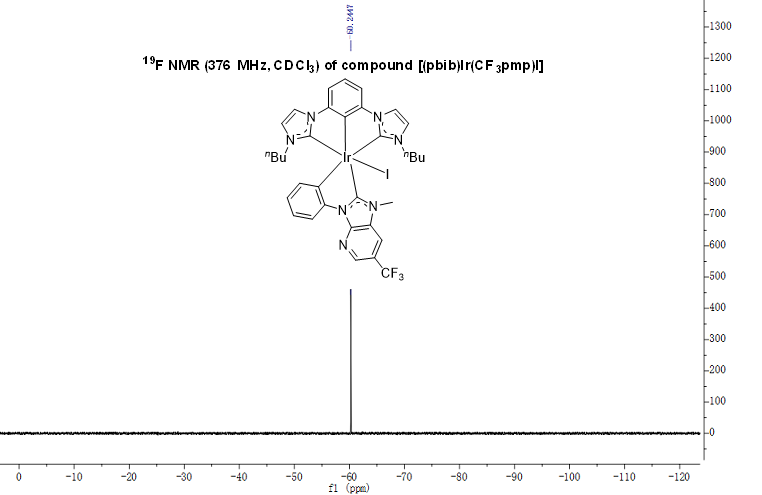


**Figure S41.** ^13^F NMR (376 MHz, CDCl_3_) of compound [(pbib)Ir(CF_3_pmp)I]


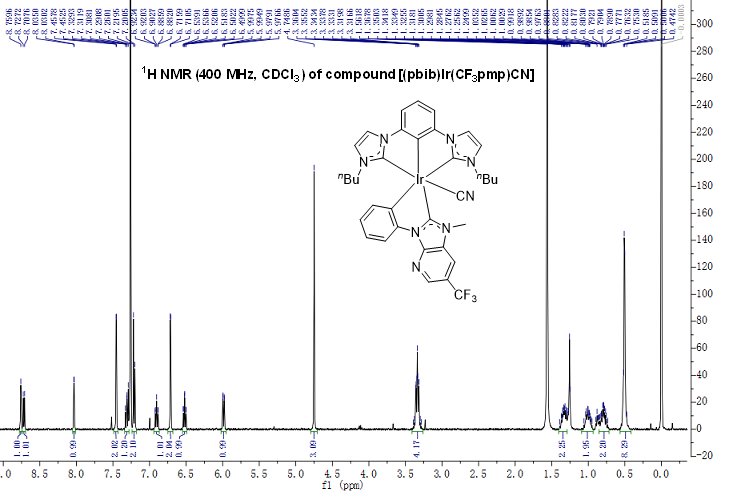


**Figure S42.** ^1^H NMR (400 MHz, CDCl_3_) of compound [(pbib)Ir(CF_3_pmp)CN] (**CF_3_-2**)


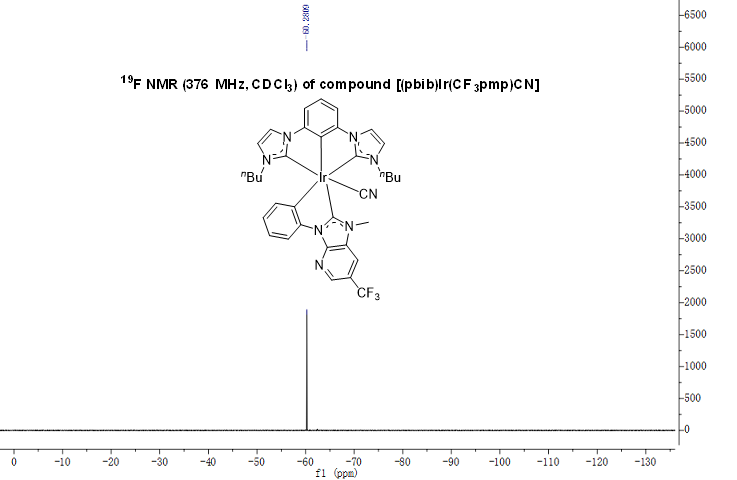


**Figure S43.** ^13^F NMR (376 MHz, CDCl_3_) of compound [(pbib)Ir(CF_3_pmp)CN] (**CF_3_-2**)


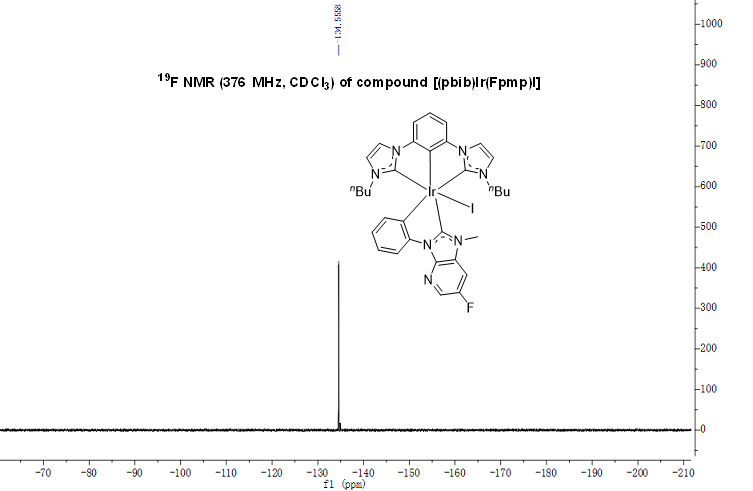


**Figure S44.** ^13^F NMR (376 MHz, CDCl_3_) of compound [(pbib)Ir(Fpmp)I]


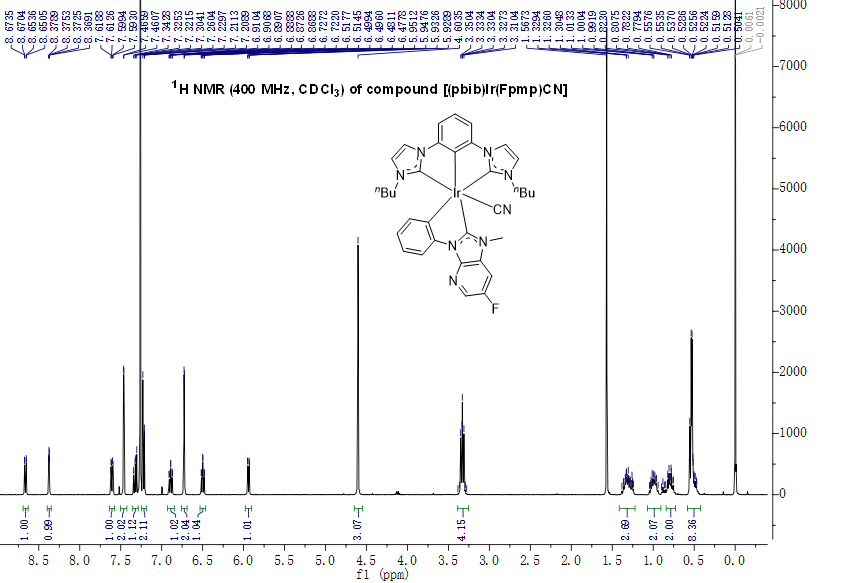


**Figure S45.** ^1^H NMR (400 MHz, CDCl_3_) of compound [(pbib)Ir(Fpmp)CN] (**F-2**)


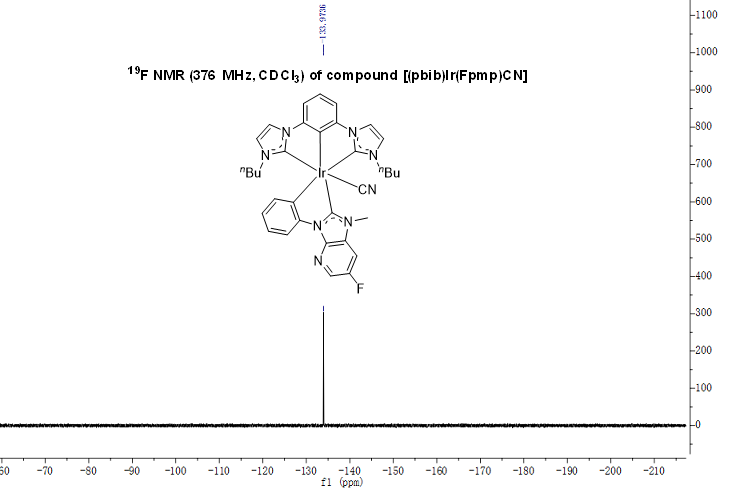


**Figure S46.** ^13^F NMR (376 MHz, CDCl_3_) of compound [(pbib)Ir(Fpmp)CN] (**F-2**)


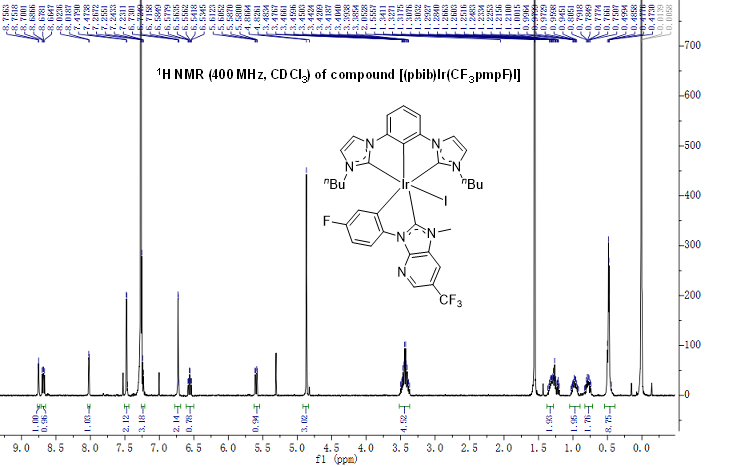


**Figure S47.** ^1^H NMR (400 MHz, CDCl_3_) of compound [(pbib)Ir(CF_3_pmpF)I]


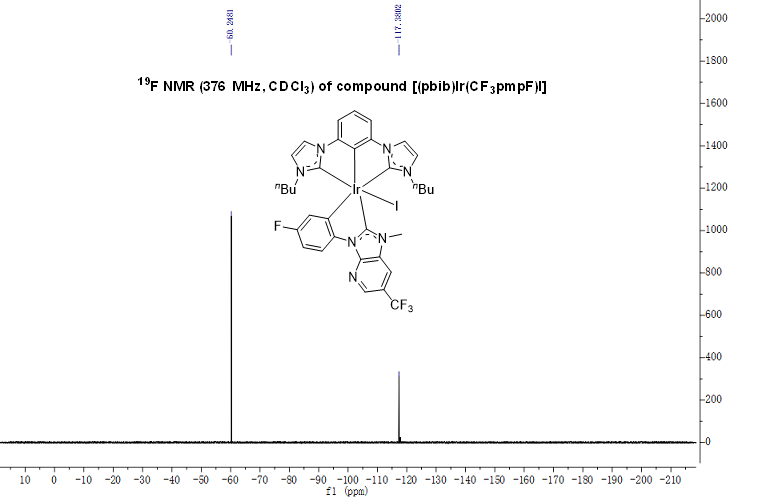


**Figure S48.** ^13^F NMR (376 MHz, CDCl_3_) of compound [(pbib)Ir(CF_3_pmpF)I]


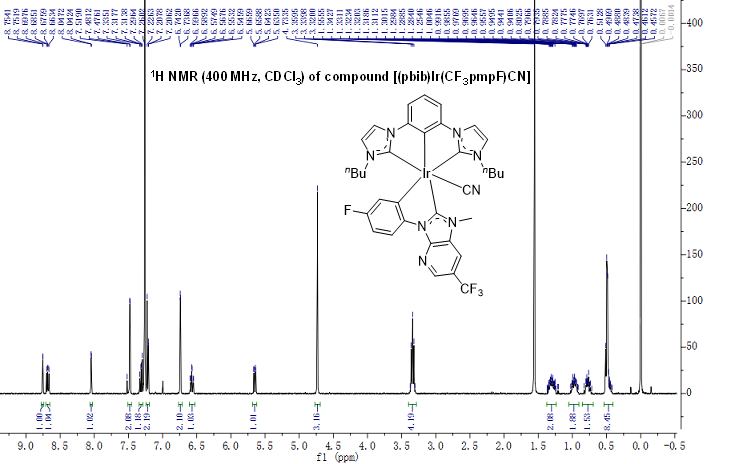


**Figure S49.** ^1^H NMR (400 MHz, CDCl_3_) of compound [(pbib)Ir(CF_3_pmpF)CN] (**CF_3_-3**)


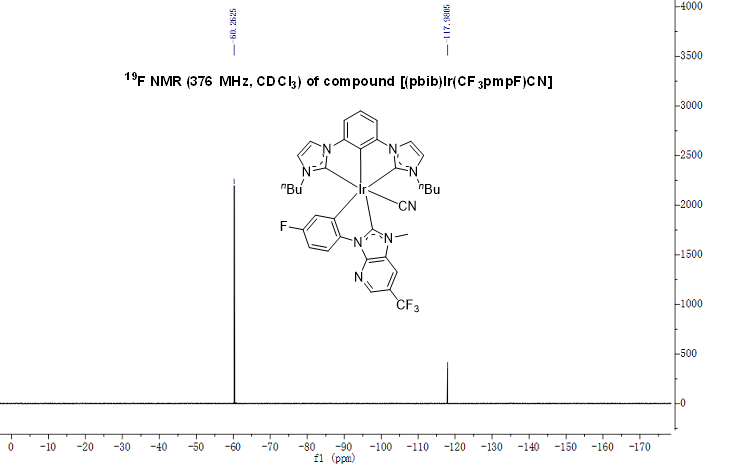


**Figure S50.** ^13^F NMR (376 MHz, CDCl_3_) of compound [(pbib)Ir(CF_3_pmpF)CN] (**CF_3_-3**)

ESI-MS


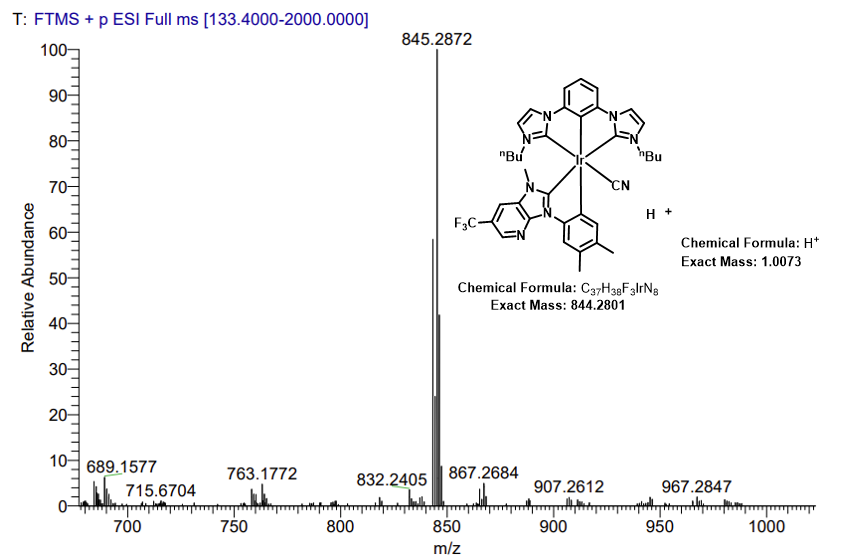


**Figure S51.** ESI-MS of **CF_3_-1**.


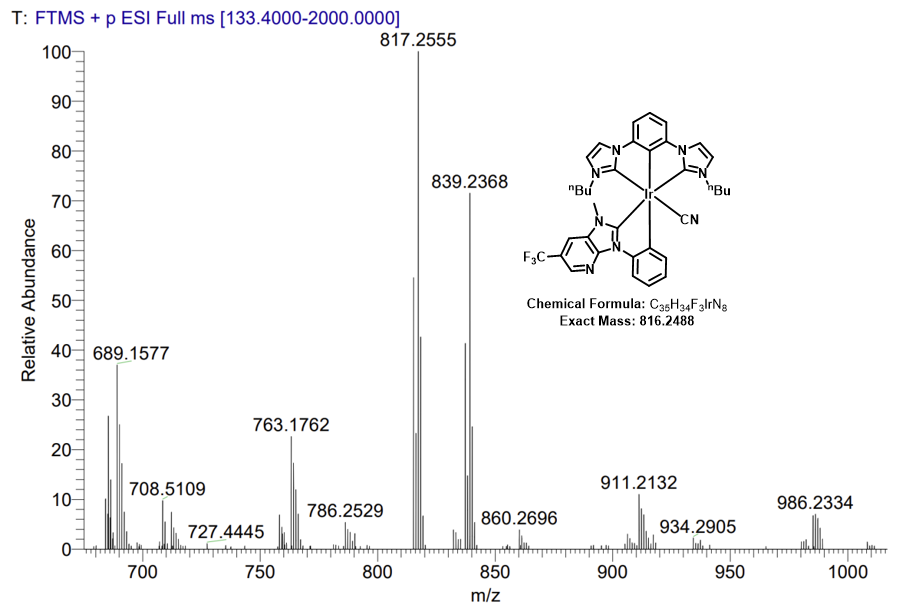


**Figure S52.** ESI-MS of **CF_3_-2**.


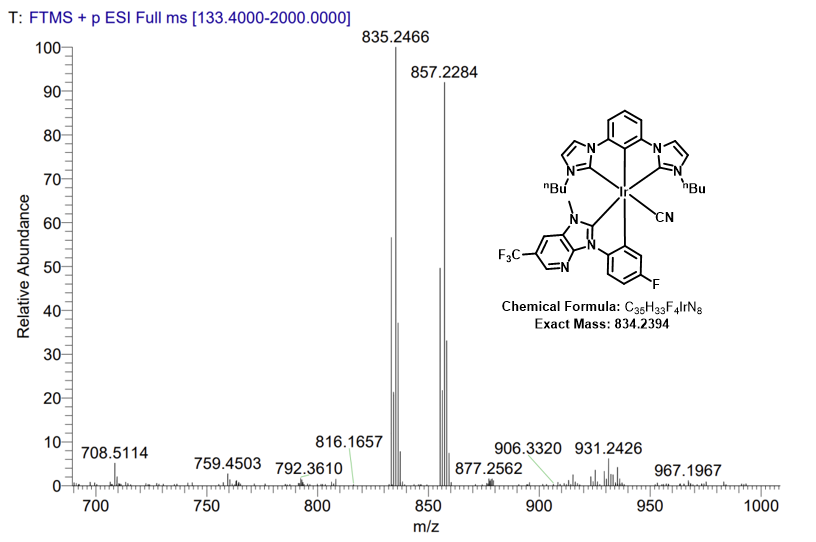


**Figure S53.** ESI-MS of **CF_3_-3**.


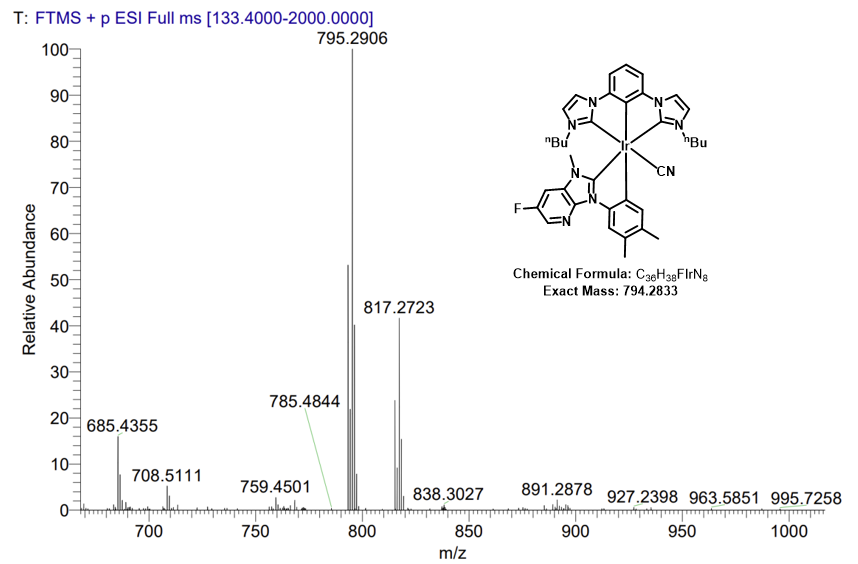


**Figure S54.** ESI-MS of **F-1**.


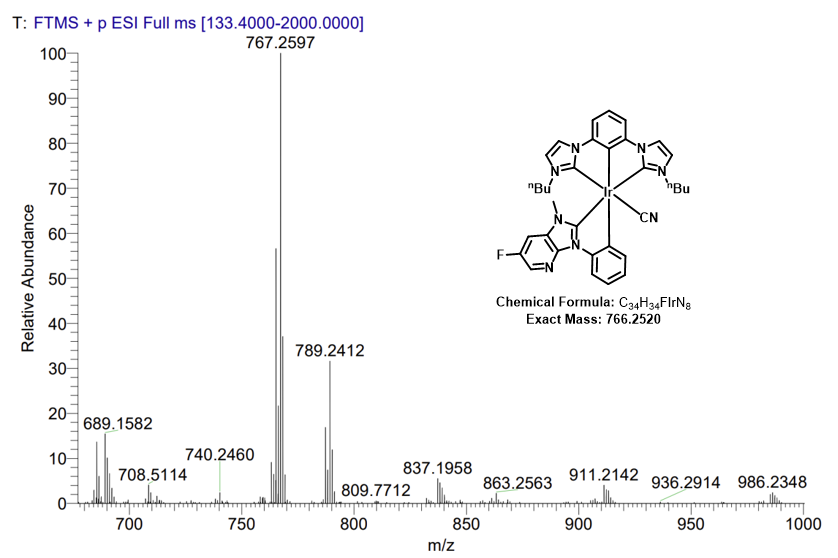


**Figure S55.** ESI-MS of **F-2**.


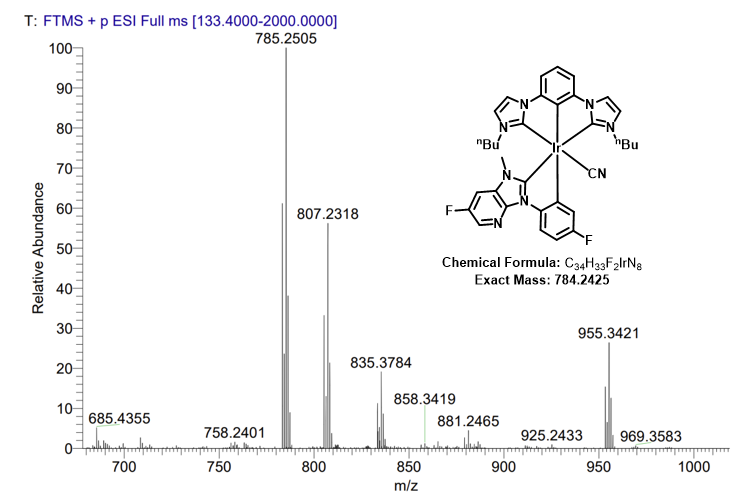


**Figure S56.** ESI-MS of **F-3**.

Thermal stability


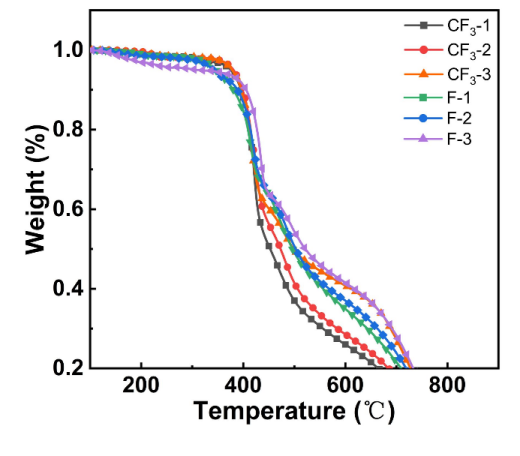


**Figure S57.** The thermogravimetric analysis (TGA) curves of studied Ir(III) complexes with decomposition temperature (T_d_) at a weight loss of 5 %.

**Table S1.** TGA data of studied Ir(III) complexes with decomposition temperature (T_d_) at a weight loss of 5 %.

| **Complex** | **T_d_ / ℃** |
| --- | --- |
| CF_3_-1 | 375 |
| CF_3_-2 | 380 |
| CF_3_-3 | 378 |
| F-1 | 345 |
| F-2 | 341 |
| F-3 | 314 |

Crystallography

**
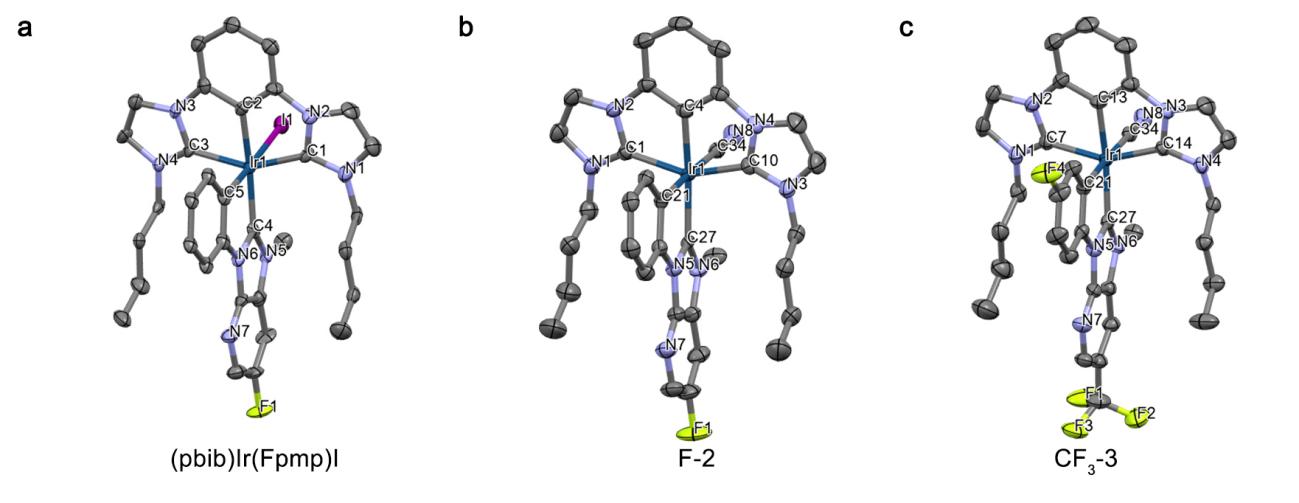
**

**Figure S58.** Molecular structure of [(pbib)Ir(Fpmp)I], F-2 and CF_3_-3 with thermal ellipsoids shown at 50% probability level. All hydrogen atoms were omitted for clarity.

**Table S2.** Selected bond lengths and angles of [(pbib)Ir(Fpmp)I], [(pbib)Ir(Fpmp)CN] (**F-2**) and [(pbib)Ir(CF_3_pmpF)CN] (**CF_3_-3**).

| **[(pbib)Ir(Fpmp)I]** | | | | | **F-2** | | | | | **CF_3_-3** | | | |
| --- | --- | --- | --- | --- | --- | --- | --- | --- | --- | --- | --- | --- | --- |
| **Atom** | | | | **Angle/ °** | **Atom** | | | **Angle/ °** | **Atom** | | | | **Angle/ °** |
| I1 | | Ir1 | C10 | 88.0 | C34 | Ir1 | C10 | 88.9 | C34 | | Ir1 | C10 | 88.5 |
|  |  | Ir1 | C4 | 84.9 |  | Ir1 | C4 | 89.7 |  |  | Ir1 | C4 | 91.2 |
|  |  | Ir1 | C1 | 86.9 |  | Ir1 | C1 | 90.0 |  |  | Ir1 | C1 | 88.9 |
|  |  | Ir1 | C27 | 103.0 |  | Ir1 | C27 | 100.1 |  |  | Ir1 | C27 | 99.9 |
|  |  | Ir1 | C21 | 178.13 |  | Ir1 | C21 | 177.2 |  |  | Ir1 | C21 | 177.2 |
| C10 | | Ir1 | C4 | 76.4 | C10 | Ir1 | C4 | 76.9 | C10 | | Ir1 | C4 | 76.9 |
|  |  | Ir1 | C1 | 153.6 |  | Ir1 | C1 | 153.5 |  |  | Ir1 | C1 | 153.5 |
|  |  | Ir1 | C27 | 103.8 |  | Ir1 | C27 | 102.4 |  |  | Ir1 | C27 | 104.2 |
|  |  | Ir1 | C21 | 92.8 |  | Ir1 | C21 | 89.6 |  |  | Ir1 | C21 | 90.2 |
| C21 | | Ir1 | C4 | 93.6 | C21 | Ir1 | C4 | 92.2 | C21 | | Ir1 | C4 | 91.0 |
|  |  | Ir1 | C1 | 91.7 |  | Ir1 | C1 | 92.5 |  |  | Ir1 | C1 | 93.4 |
|  |  | Ir1 | C27 | 78.4 |  | Ir1 | C27 | 77.9 |  |  | Ir1 | C27 | 77.9 |
| **Atom** | | | **Length/** **Å** | | **Atom** | | **Length/ Å** | | **Atom** | | | **Length/ Å** | |
| Ir1 | C34 | | 2.776 | | Ir1 | C34 | 2.044 | | Ir1 | | C34 | 2.043 | |
|  | C10 | | 2.065 | |  | C10 | 2.065 | |  |  | C10 | 2.055 | |
|  | C4 | | 2.002 | |  | C4 | 2.014 | |  |  | C4 | 2.012 | |
|  | C1 | | 2.063 | |  | C1 | 2.063 | |  |  | C1 | 2.061 | |
|  | C27 | | 2.060 | |  | C27 | 2.065 | |  |  | C27 | 2.051 | |
|  | C21 | | 2.072 | |  | C21 | 2.083 | |  |  | C21 | 2.091 | |

**
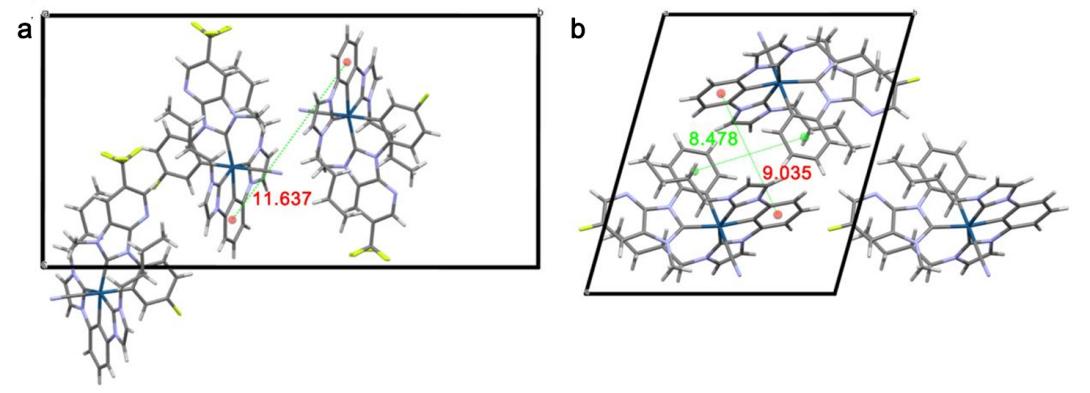
**

**Figure S59.** Intermolecular interactions of **CF_3_-3** (left) and **F-2** (right). The unit of length is the angstrom (Å).

Photophysical properties


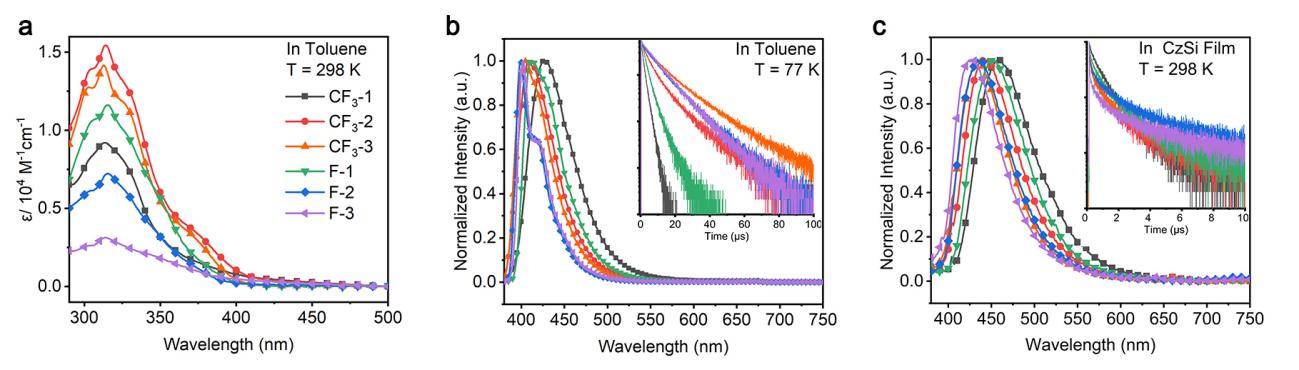


**Figure S60.** A) Absorption spectra of Ir(III) complexes in toluene solution at 298 K; B) Emission spectra and lifetime decay profiles of all complexes in toluene solution at 77 K; C) Emission spectra and lifetime decay profiles of all complexes in 12.5% CzSi film.

**Table S3.** Photophysical properties of studied Ir(III) complexes in different solution at 298 K.

| **Complex** | **Medium (T [K])** | **Λ_max_^a^ (nm)** | **Τ^a^ (μs)** |
| --- | --- | --- | --- |
| CF_3_-1 | DCM (298 K) | 482 | 0.63 |
|  | THF (298 K) | 476 | 0.89 |
|  | DMF (298 K) | 489 | 0.75 |
| CF_3_-2 | DCM (298 K) | 457 | 0.59 |
|  | THF (298 K) | 453 | 0.82 |
|  | DMF (298 K) | 471 | 0.72 |
| CF_3_-3 | DCM (298 K) | 437 | 0.67 |
|  | THF (298 K) | 445 | 0.89 |
|  | DMF (298 K) | 461 | 0.86 |
| F-1 | DCM (298 K) | 466 | 0.74 |
|  | THF (298 K) | 452 | 1.19 |
|  | DMF (298 K) | 465 | 0.90 |
| F-2 | DCM (298 K) | 433 | 1.04 |
|  | THF (298 K) | 430 | 1.16 |
|  | DMF (298 K) | 440 | 1.35 |
| F-3 | DCM (298 K) | 426 | 1.05 |
|  | THF (298 K) | 424 | 1.19 |
|  | DMF (298 K) | 430 | 1.37 |


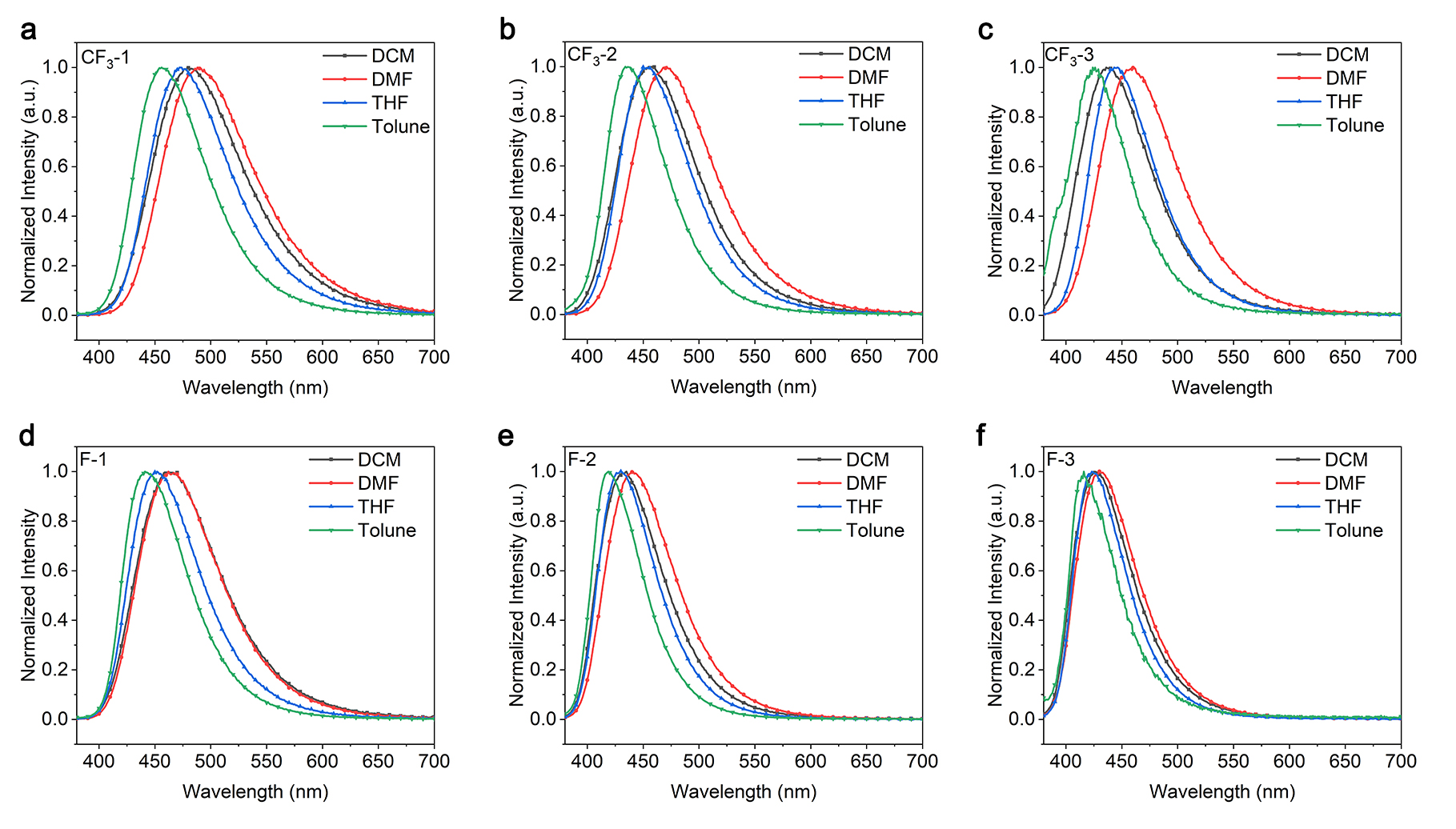


**Figure S61.** Emission spectra of studied Ir(III) complexes in different solution at 298 K.


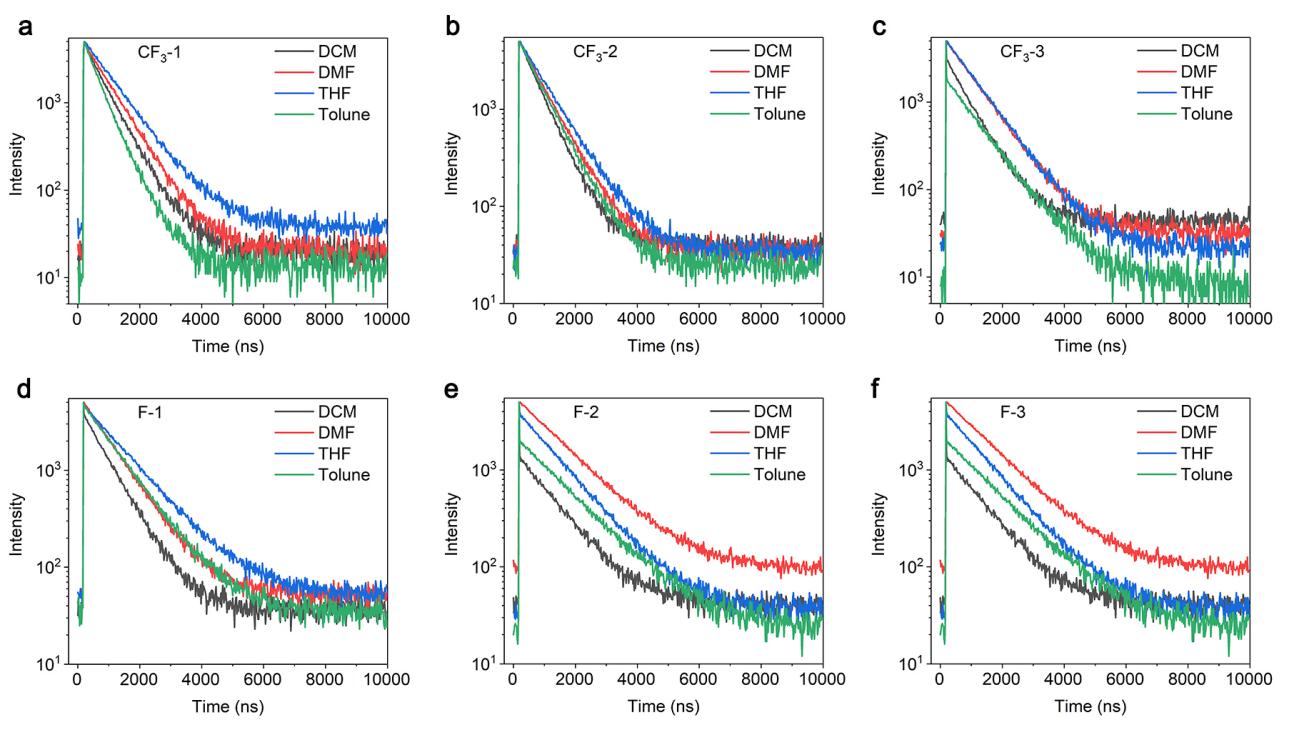


**Figure S62.** Lifetime decay profiles of studied Ir(III) complexes in different solution at 298 K.

Electrochemical properties


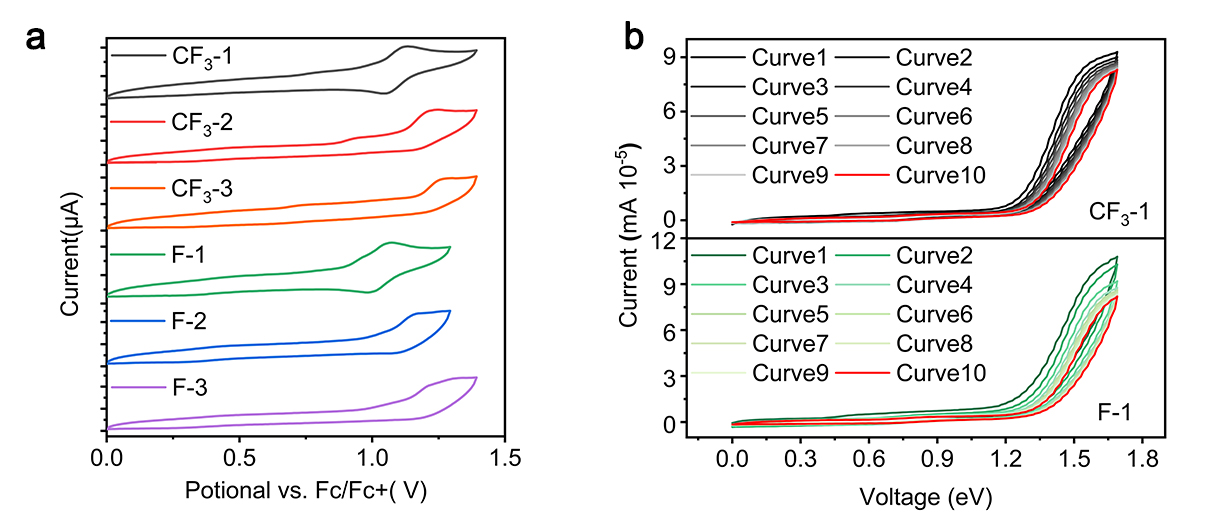


**Figure S63.** A) Cyclic voltammograms and B) Multiscan cyclic voltammograms of studied Ir(III) complexes in tetrabutylammonium hexafluorophosphate (0.1 M) solution in dichloromethane

**DFT / TD-DFT Calculation**


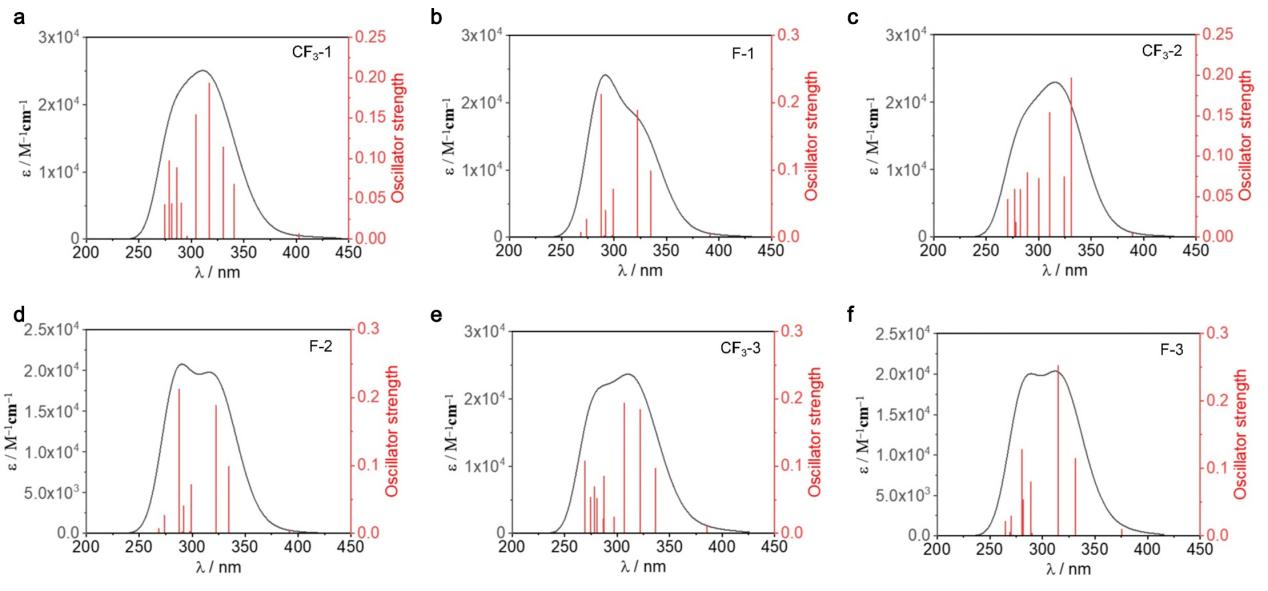


**Figure S64.** Simulated absorption spectrum of the six iridium complexes. The red vertical lines refer to the unbroadened oscillator strengths of the singlet–singlet transitions, and the black line is the fitting line of the UV-vis absorption.


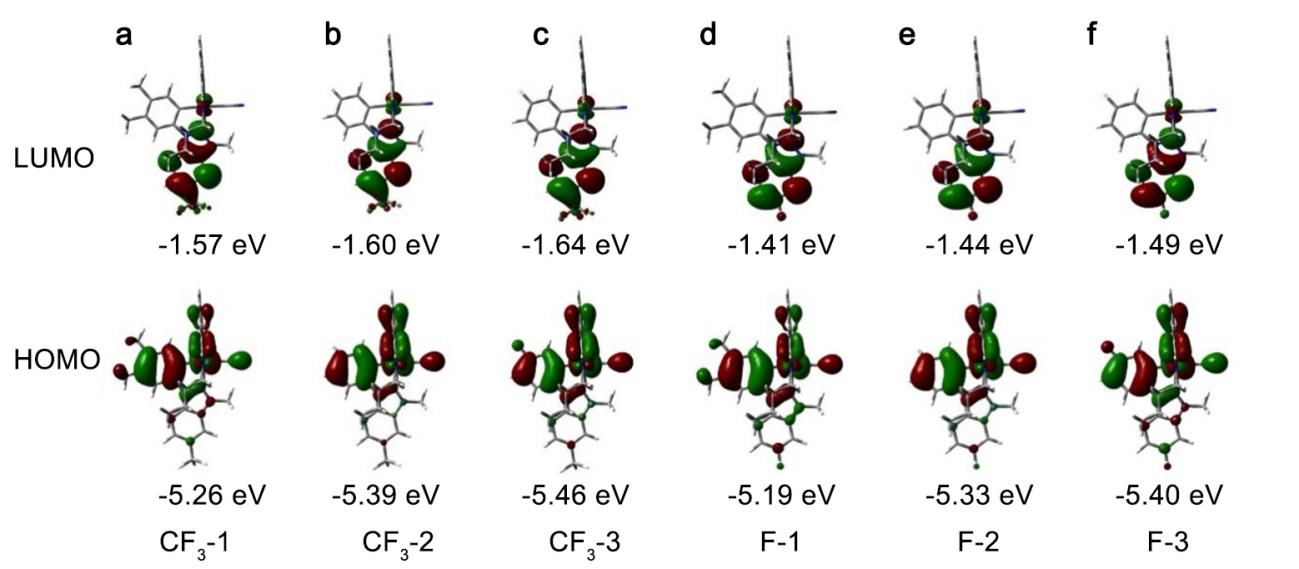


**Figure S65.** Frontier molecular orbitals (HOMO and LUMO) of the studied Ir(III) complexes at their geometries optimized for the ground state.


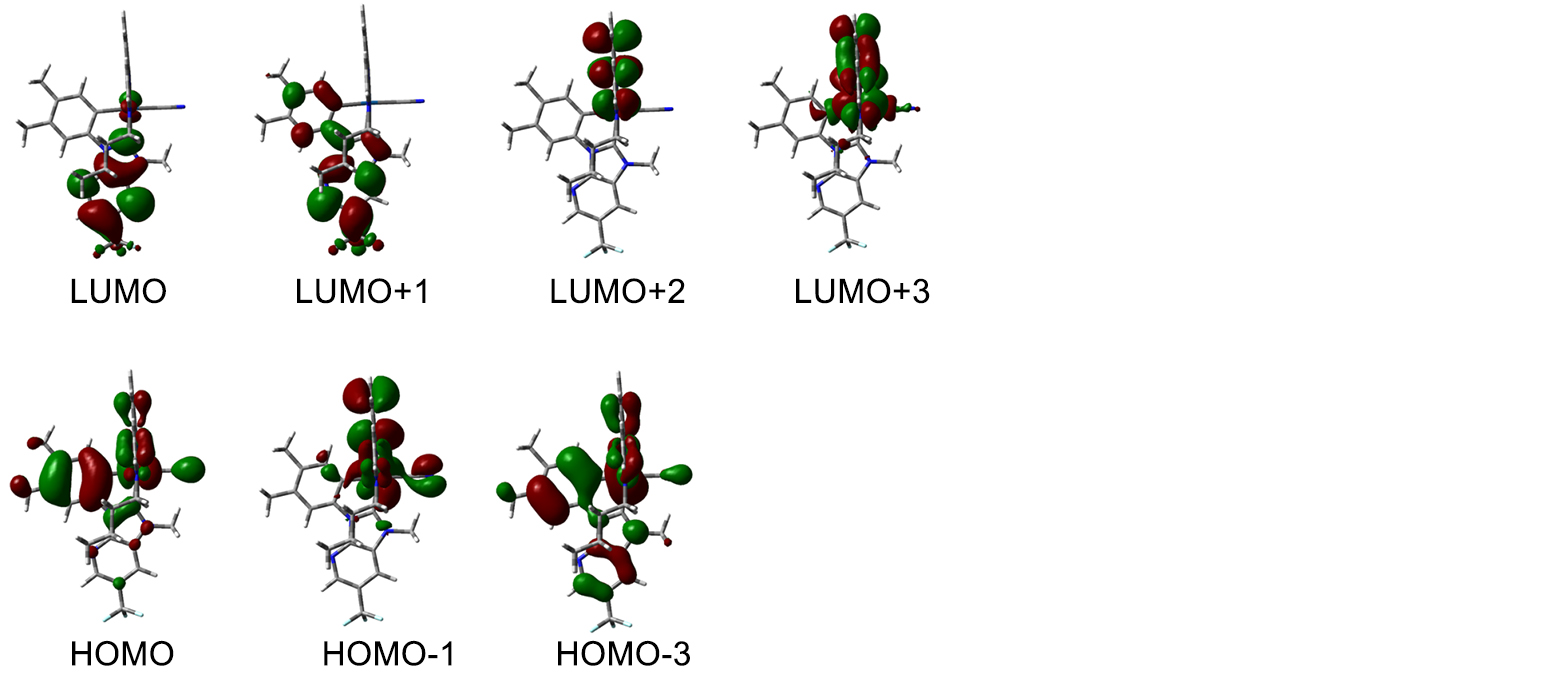


**Figure S66.** Spatial plots (isovalue = 0.03) of selected molecular orbitals of **CF_3_-1** at the optimized B3LYP ground-state geometry.


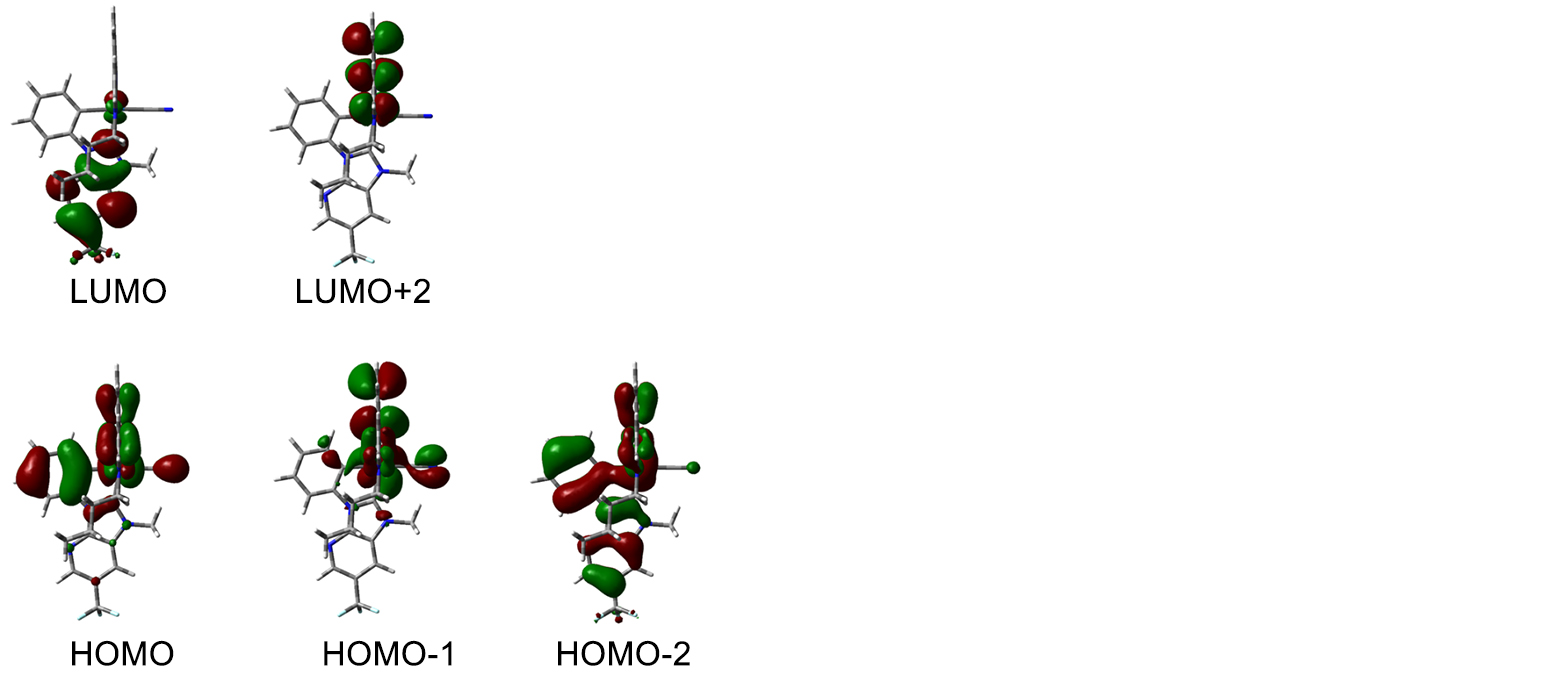


**Figure S67.** Spatial plots (isovalue = 0.03) of selected molecular orbitals of **CF_3_-2** at the optimized B3LYP ground-state geometry.


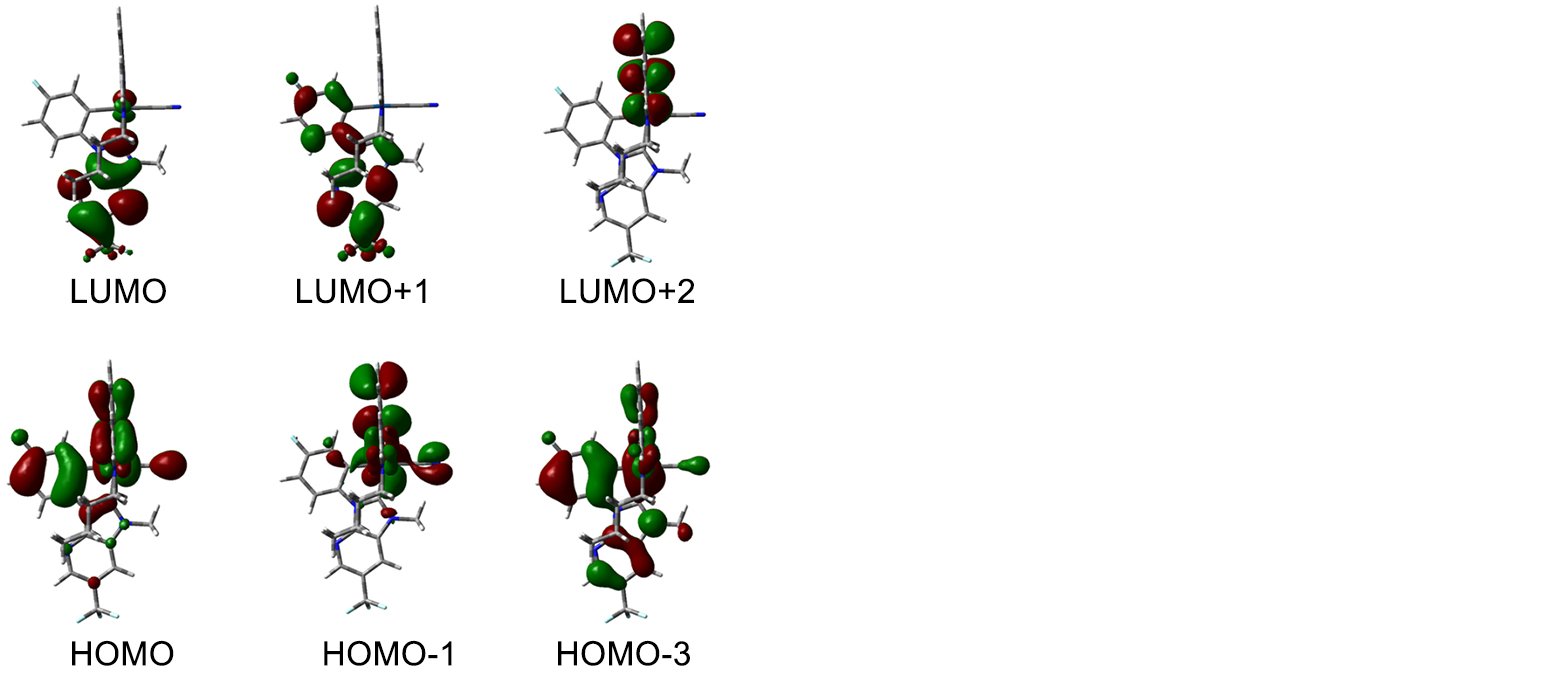


**Figure S68.** Spatial plots (isovalue = 0.03) of selected molecular orbitals of **CF_3_-3** at the optimized B3LYP ground-state geometry.


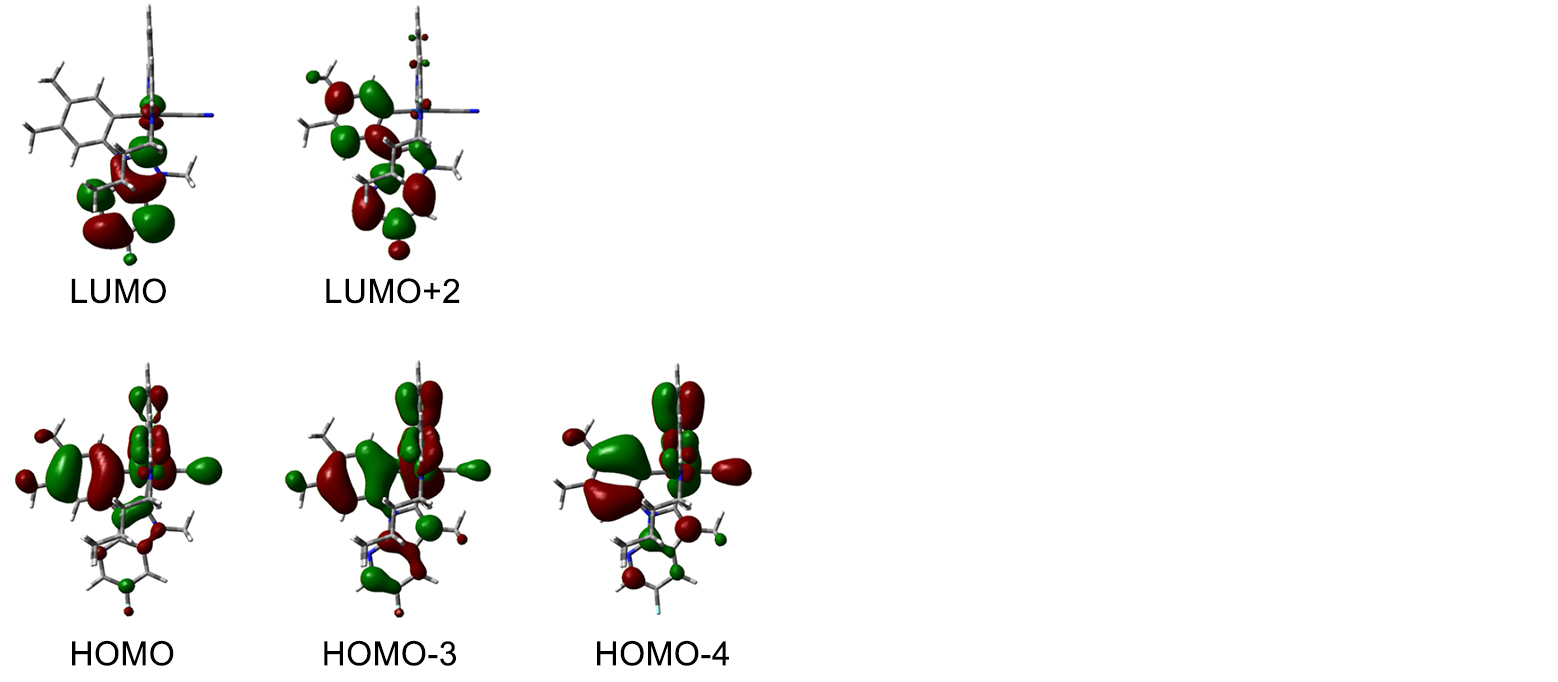


**Figure S69.** Spatial plots (isovalue = 0.03) of selected molecular orbitals of **F-1** at the optimized B3LYP ground-state geometry.

**
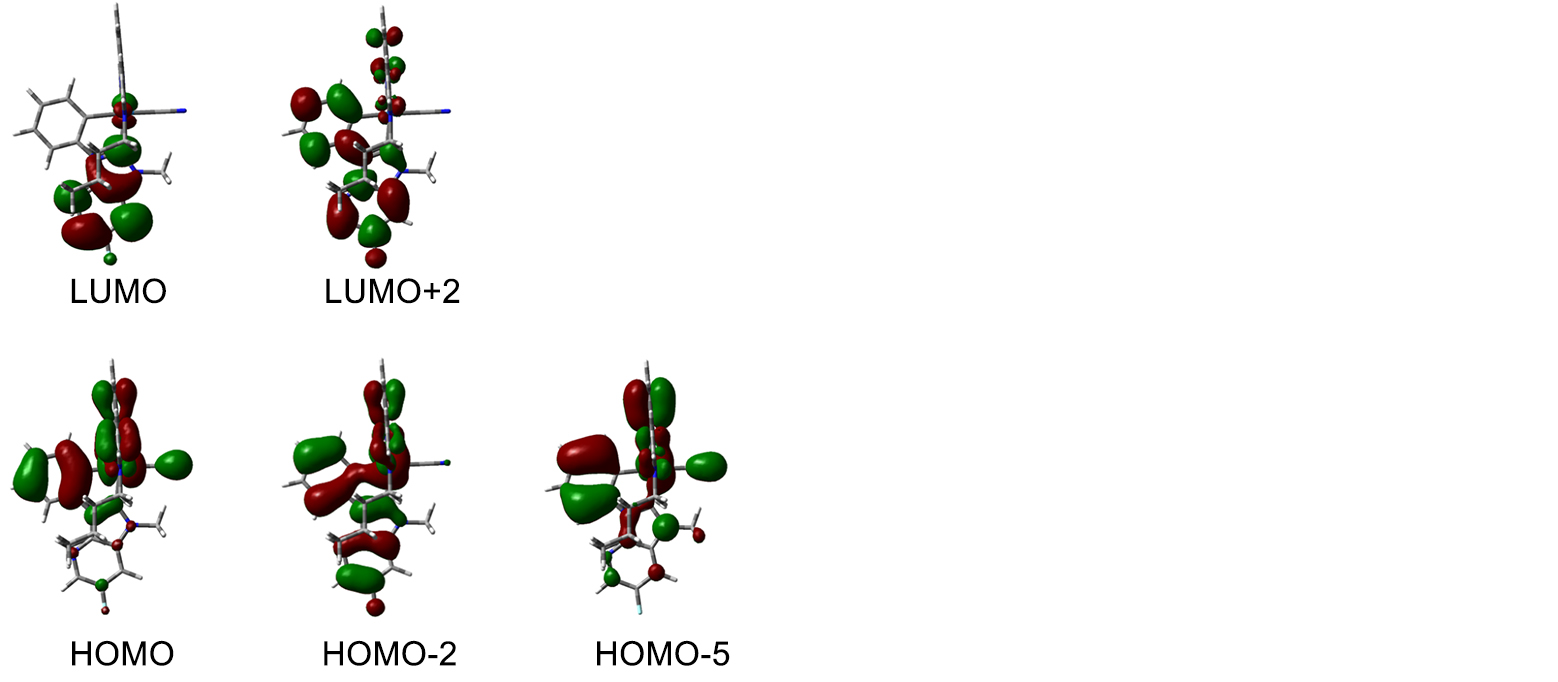
**

**Figure S70.** Spatial plots (isovalue = 0.03) of selected molecular orbitals of **F-2** at the optimized B3LYP ground-state geometry.


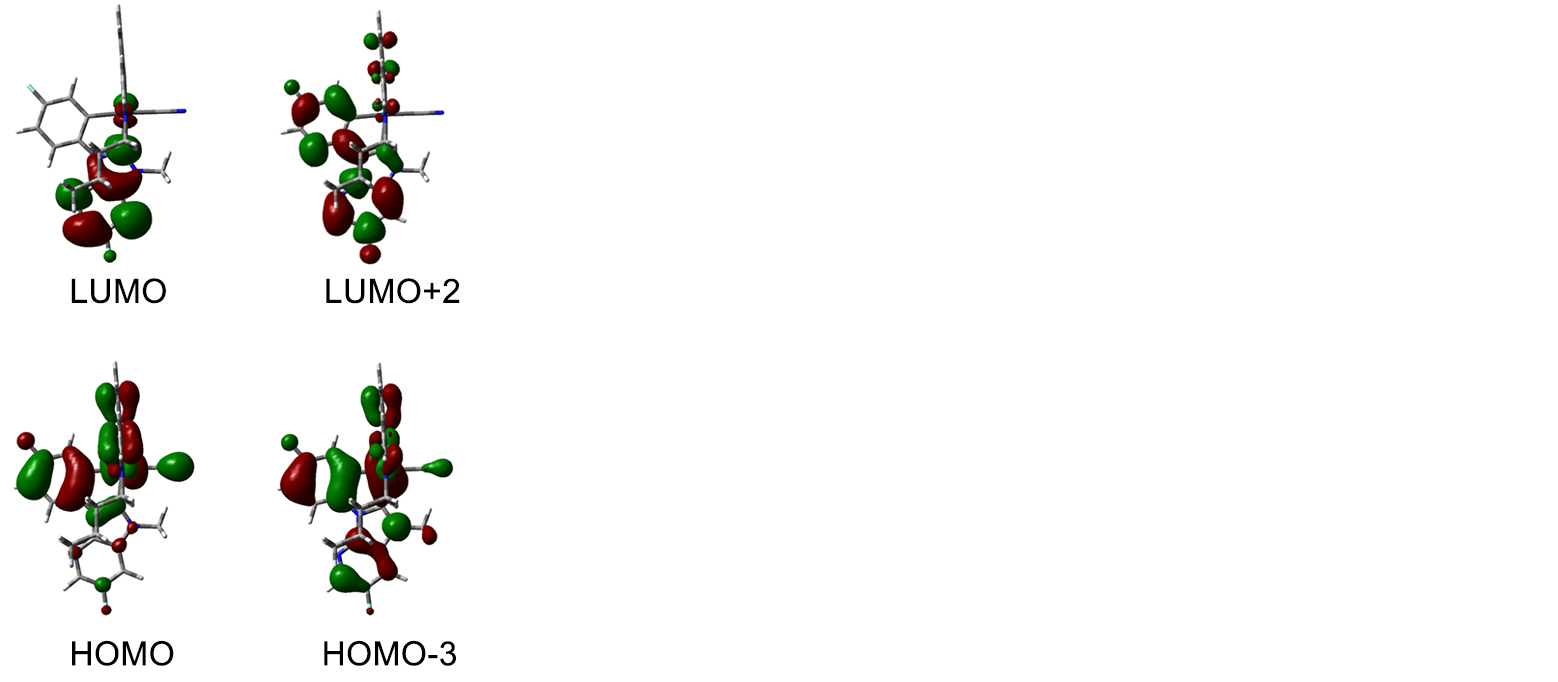


**Figure S71.** Spatial plots (isovalue = 0.03) of selected molecular orbitals of **F-3** at the optimized B3LYP ground-state geometry.


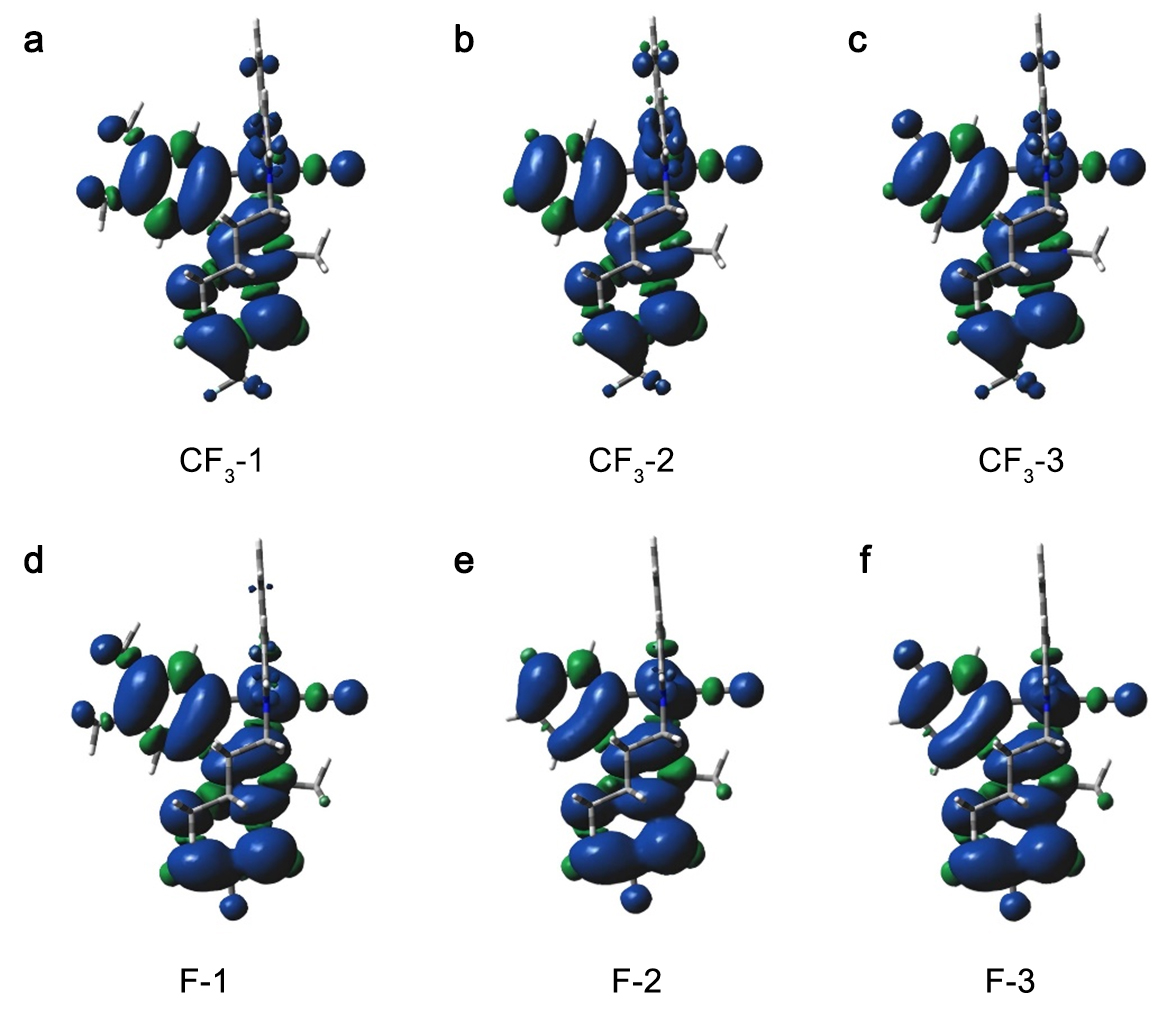


**Figure S72.** Plots of spin density (isovalue = 0.002) of the T_1_ states of the six iridium complexes optimized at the PBE0 level.

**Table S4**. Calculated first 15 singlet excited state energies (λ / nm), the associated oscillator strengths (*f*) and the nature of the transitions at the optimized ground state (S_0_) geometries of the six iridium complexes in the dichloromethane (DCM) by TD-B3LYP. The values in the parentheses are the % contributions of that particular configuration state function (CSF).

| **CF_3_-1** | Energy (eV) | λ (nm) | ƒ | Major contribs |
| --- | --- | --- | --- | --- |
| 1 | 3.0819 | 402.3 | 0.0068 | H → L 98.4% |
| 2 | 3.3366 | 371.59 | 0.0000 | H-1 → L 97.3% |
| 3 | 3.6372 | 340.88 | 0.0681 | H-2 → L 94.6% |
| 4 | 3.7504 | 330.59 | 0.1142 | H-3 → L 80.7%, H → L+1 14.5% |
| 5 | 3.9132 | 316.84 | 0.1934 | H → L+1 78.3%, H-3 → L 15.9% |
| 6 | 4.075 | 304.26 | 0.1544 | H-4 → L 84.6% |
| 7 | 4.1489 | 298.84 | 0.0004 | H-1 → L+1 58.4%, H-5 → L 38.1% |
| 8 | 4.1608 | 297.98 | 0.0001 | H-5 → L 60.0%, H-1 → L+1 38.3% |
| 9 | 4.1908 | 295.85 | 0.0037 | H → L+2 89.8% |
| 10 | 4.27 | 290.36 | 0.0447 | H-1 → L+2 73.0%,  H → L+3 21.5% |
| 11 | 4.3297 | 286.36 | 0.0883 | H → L+3 74.0%, H-1 → L+2 20.0% |
| 12 | 4.3464 | 285.26 | 0.0011 | H-7 → L 95.0% |
| 13 | 4.4089 | 281.21 | 0.0439 | H-2 → L+1 60.6%, H-6 → L 24.2% |
| 14 | 4.4493 | 278.66 | 0.097 | H-6 → L 50.8%, H-2 → L+1 19.6%, H-3 → L+1 19.5%, H → L+4 5.7% |
| 15 | 4.5114 | 274.82 | 0.043 | H-3 → L+1 45.9%, H → L+4 23.7%, H-6 → L 17.2%,  H-2 → L+1 8.4% |
| **CF_3_-2** | Energy (eV) | λ (nm) | ƒ | Major contribs |
| 1 | 3.1883 | 388.87 | 0.0054 | H → L 97.5% |
| 2 | 3.3388 | 371.34 | 0 | H-1 → L 97.2% |
| 3 | 3.7444 | 331.12 | 0.1965 | H-2 → L 91.5% |
| 4 | 3.8225 | 324.35 | 0.0745 | H-3 → L 86.1%, H → L+1 6.1% |
| 5 | 3.9946 | 310.38 | 0.1539 | H → L+1 85.2%, H-3 → L 7.6% |
| 6 | 4.1308 | 300.15 | 0.0007 | H-1 → L+1 89.0%, H-4 → L 6.9% |
| 7 | 4.1328 | 300 | 0.0722 | H-5 → L 84.6% |
| 8 | 4.1572 | 298.24 | 0 | H-4 → L 91.2%, H-1 → L+1 7.3% |
| 9 | 4.2824 | 289.52 | 0.0196 | H → L+2 89.1%, H-1 → L+3 5.6% |
| 10 | 4.2894 | 289.05 | 0.0795 | H-1 → L+2 88.7% |
| 11 | 4.3539 | 284.77 | 0.0001 | H-6 → L 95.9% |
| 12 | 4.3888 | 282.5 | 0.0587 | H → L+3 92.9% |
| 13 | 4.457 | 278.18 | 0.0182 | H-7 → L 67.7%, H-2 → L+1 19.7% |
| 14 | 4.4773 | 276.92 | 0.059 | H-2 → L+1 64.2%, H-7 → L 15.7%, H-3 → L+1 14.0% |
| 15 | 4.581 | 270.65 | 0.0462 | H → L+4 48.4%, H-3 → L+1 29.6%, H-7 → L 9.6%, H-2 → L+1 6.1% |
| **CF_3_-3** | Energy (eV) | λ (nm) | ƒ | Major contribs |
| 1 | 3.2164 | 385.48 | 0.0104 | H → L 97.3% |
| 2 | 3.353 | 369.77 | 0 | H-1 → L 97.2% |
| 3 | 3.6827 | 336.67 | 0.097 | H-2 → L 93.4% |
| 4 | 3.8507 | 321.98 | 0.1843 | H-3 → L 84.0%, H → L+1 8.8% |
| 5 | 4.0419 | 306.75 | 0.1936 | H → L+1 85.9%, H-3 → L 9.4% |
| 6 | 4.1623 | 297.87 | 0.0007 | H-1 → L+1 67.4%, H-5 → L 28.7% |
| 7 | 4.1723 | 297.16 | 0.0243 | H-4 → L 90.8% |
| 8 | 4.1776 | 296.78 | 0.0003 | H-5 → L 69.3%, H-1 → L+1 29.4% |
| 9 | 4.3091 | 287.73 | 0.0851 | H-1 → L+2 90.2% |
| 10 | 4.3237 | 286.75 | 0.0214 | H → L+2 88.6%, H-1 → L+3 6.7% |
| 11 | 4.3892 | 282.48 | 0 | H-6 → L 96.3% |
| 12 | 4.4167 | 280.72 | 0.0512 | H → L+3 93.6% |
| 13 | 4.4559 | 278.25 | 0.069 | H-2 → L+1 83.5%, H-7 → L 7.7% |
| 14 | 4.5141 | 274.66 | 0.0538 | H-7 → L 78.9%, H-2 → L+1 7.6%, H-3 → L+1 5.2% |
| 15 | 4.602 | 269.41 | 0.1077 | H-3 → L+1 62.7%, H-1 → L+3 24.2% |
| **F-1** | Energy (eV) | λ (nm) | ƒ | Major contribs |
| 1 | 3.1682 | 391.34 | 0.0055 | H → L 98.4% |
| 2 | 3.4387 | 360.56 | 0 | H-1 → L 97.2% |
| 3 | 3.7028 | 334.84 | 0.0989 | H-2 → L 95.4% |
| 4 | 3.8476 | 322.24 | 0.1888 | H-3 → L 92.8% |
| 5 | 4.1445 | 299.15 | 0.0718 | H → L+1 46.3%, H-4 → L 43.6% |
| 6 | 4.164 | 297.75 | 0.0028 | H → L+1 48.5%, H-4 → L 41.1% |
| 7 | 4.2519 | 291.6 | 0.0403 | H-1 → L+1 68.6%, H → L+3 23.3% |
| 8 | 4.2661 | 290.63 | 0.0021 | H-5 → L 95.4% |
| 9 | 4.3109 | 287.61 | 0.2123 | H → L+2 82.8%, H-4 → L 7.4% |
| 10 | 4.313 | 287.47 | 0.0912 | H → L+3 72.1%, H-1 → L+1 22.2% |
| 11 | 4.4485 | 278.71 | 0 | H-7 → L 94.0% |
| 12 | 4.5289 | 273.76 | 0.0265 | H-6 → L 68.2%, H → L+4 15.1%, H-1 → L+3 8.3% |
| 13 | 4.5438 | 272.86 | 0.0004 | H-1 → L+2 86.8%, H-1 → L+4 8.3% |
| 14 | 4.5702 | 271.29 | 0.0002 | H-1 → L+3 66.1%, H-2 → L+1 19.4% |
| 15 | 4.6214 | 268.28 | 0.0074 | H-2 → L+1 42.3%, H-3 → L+1 30.1%, H → L+4 14.3%, H-1 → L+3 8.2% |
| **F-2** | Energy (eV) | λ (nm) | ƒ | Major contribs |
| 1 | 3.277 | 378.35 | 0.0045 | H → L 97.6% |
| 2 | 3.4393 | 360.49 | 0 | H-1 → L 97.1% |
| 3 | 3.784 | 327.65 | 0.2159 | H-2 → L 94.6% |
| 4 | 3.9222 | 316.11 | 0.126 | H-3 → L 90.4% |
| 5 | 4.2197 | 293.82 | 0.0289 | H-5 → L 80.8%, H-3 → L 6.5% |
| 6 | 4.2503 | 291.71 | 0.047 | H → L+1 90.3% |
| 7 | 4.2633 | 290.82 | 0.0016 | H-4 → L 94.7% |
| 8 | 4.2723 | 290.2 | 0.0767 | H-1 → L+1 81.9% |
| 9 | 4.3692 | 283.77 | 0.1293 | H → L+2 82.2%, H-5 → L 5.3% |
| 10 | 4.3736 | 283.48 | 0.0595 | H → L+3 92.1% |
| 11 | 4.4519 | 278.5 | 0.0001 | H-6 → L 89.3% |
| 12 | 4.5053 | 275.2 | 0.001 | H-1 → L+2 79.8%, H-6 → L 7.0%, H-1 → L+4 6.8% |
| 13 | 4.5544 | 272.23 | 0.0294 | H-7 → L 74.3%, H-1 → L+3 13.0% |
| 14 | 4.5983 | 269.63 | 0.0034 | H-1 → L+3 68.8%, H-7 → L 11.9%, H-2 → L+1 8.0% |
| 15 | 4.6607 | 266.02 | 0.005 | H-2 → L+1 69.5%, H-3 → L+1 17.2% |
| **F-3** | Energy (eV) | λ (nm) | ƒ | Major contribs |
| 1 | 3.3005 | 375.65 | 0.0093 | H → L 97.4% |
| 2 | 3.4544 | 358.92 | 0 | H-1 → L 97.2% |
| 3 | 3.7413 | 331.39 | 0.1149 | H-2 → L 94.7% |
| 4 | 3.9369 | 314.93 | 0.2523 | H-3 → L 91.3% |
| 5 | 4.272 | 290.23 | 0.0028 | H-4 → L 88.7% |
| 6 | 4.2829 | 289.49 | 0.0038 | H-5 → L 92.4% |
| 7 | 4.2903 | 288.99 | 0.0488 | H → L+1 90.9%, H-1 → L+3 5.1% |
| 8 | 4.2924 | 288.85 | 0.08 | H-1 → L+1 82.3%, H-5 → L 5.4% |
| 9 | 4.4004 | 281.76 | 0.053 | H → L+3 92.3% |
| 10 | 4.4201 | 280.5 | 0.1274 | H → L+2 84.5% |
| 11 | 4.4891 | 276.19 | 0 | H-6 → L 93.7% |
| 12 | 4.558 | 272.01 | 0.0008 | H-1 → L+2 85.3%, H-1 → L+4 6.7% |
| 13 | 4.5845 | 270.44 | 0.0297 | H-1 → L+3 51.8%, H-7 → L 31.7% |
| 14 | 4.6123 | 268.81 | 0.0052 | H-7 → L 52.9%, H-1 → L+3 22.8%, H-2 → L+1 13.0% |
| 15 | 4.6849 | 264.65 | 0.021 | H-2 → L+1 57.7%, H-3 → L+1 20.1%, H-1 → L+3 10.6% |

**Table S5**. Relative energies of the lowest triplet excited states (T_1_) of the six iridium complexes optimized at the B3LYP level.

| **Complex** | **ΔE/ eV (T_1_-S_0_) ^a^** | **λ/ nm ^a^** | **λ/ nm ^b^** |
| --- | --- | --- | --- |
| CF_3_-1 | 2.9662 | 418 | 456 |
| CF_3_-2 | 2.9752 | 417 | 437 |
| CF_3_-3 | 3.0146 | 411 | 425 |
| F-1 | 3.0001 | 413 | 442 |
| F-2 | 3.0333 | 409 | 419 |
| F-3 | 3.0659 | 404 | 416 |

*^a^* Energy difference between the lowest triplet excited state (T_1_) and the ground state (S_0_) at the corresponding optimized geometries in toluene solution; ^b^ the emission values in the bracket are from the experimental data.

**Table S6**. Cartesian coordinates for optimized structures of **CF_3_-1**.

| Ground state of **CF_3_-1** (S_0_) | | | | Excited state of **CF_3_-1** (T_1_) | | | |
| --- | --- | --- | --- | --- | --- | --- | --- |
| Ir | 1.429575 | 0.011856 | -0.78515 | Ir | 1.467456 | 0.001874 | -0.7124 |
| N | 1.264828 | 3.229381 | -0.74131 | N | 1.330798 | 3.224778 | -0.82444 |
| N | 3.202668 | 2.323294 | -0.45387 | N | 3.250077 | 2.306884 | -0.44035 |
| N | 3.244342 | -2.27426 | -0.50811 | N | 3.255457 | -2.30005 | -0.44952 |
| N | 1.322567 | -3.208 | -0.81399 | N | 1.338291 | -3.2209 | -0.83698 |
| C | 2.179442 | 4.26416 | -0.55102 | C | 2.256623 | 4.253136 | -0.67354 |
| H | 1.880244 | 5.299544 | -0.56866 | H | 1.975649 | 5.290423 | -0.7575 |
| N | -1.67433 | -0.00547 | -1.65767 | N | -1.70627 | 0.001153 | -1.59863 |
| N | -1.20748 | -0.02112 | 0.488491 | N | -1.22355 | -0.00379 | 0.532476 |
| N | -3.48404 | -0.04921 | 1.37667 | N | -3.48341 | -0.00812 | 1.488083 |
| C | 4.102379 | 1.229174 | -0.33605 | C | 4.138458 | 1.205121 | -0.28052 |
| C | -0.17804 | 3.407092 | -0.88001 | C | -0.1056 | 3.402773 | -1.04545 |
| H | -0.54123 | 2.577081 | -1.4876 | H | -0.4393 | 2.548093 | -1.63496 |
| H | -0.35114 | 4.33231 | -1.43756 | H | -0.24027 | 4.306235 | -1.64667 |
| C | 1.873435 | 2.023813 | -0.67693 | C | 1.919177 | 2.019904 | -0.67657 |
| C | 4.124233 | -1.16716 | -0.36467 | C | 4.141276 | -1.19687 | -0.28534 |
| C | -0.6249 | -0.00528 | -0.77378 | C | -0.62984 | -0.00022 | -0.75923 |
| C | -0.11773 | -3.40739 | -0.94954 | C | -0.09777 | -3.40132 | -1.05805 |
| H | -0.278 | -4.3267 | -1.52048 | H | -0.2307 | -4.3029 | -1.66248 |
| H | -0.49771 | -2.5742 | -1.54243 | H | -0.43368 | -2.54525 | -1.64428 |
| C | 3.399596 | 3.692767 | -0.37558 | C | 3.465378 | 3.674875 | -0.43623 |
| H | 4.369347 | 4.134187 | -0.21693 | H | 4.437499 | 4.112432 | -0.28121 |
| C | -1.57353 | 0.007678 | -3.11143 | C | -1.63929 | 0.004772 | -3.04983 |
| H | -2.0681 | 0.899418 | -3.50737 | H | -2.14422 | 0.894121 | -3.44045 |
| H | -2.05808 | -0.88275 | -3.52248 | H | -2.14485 | -0.88227 | -3.44482 |
| H | -0.52291 | 0.016248 | -3.39265 | H | -0.5968 | 0.005175 | -3.36064 |
| C | -2.88637 | -0.01772 | -0.98322 | C | -2.91879 | -0.00118 | -0.90892 |
| C | -2.58667 | -0.02932 | 0.399369 | C | -2.61586 | -0.00465 | 0.457056 |
| C | 2.255498 | -4.2304 | -0.64722 | C | 2.26648 | -4.24769 | -0.68999 |
| H | 1.974332 | -5.27024 | -0.687 | H | 1.987852 | -5.2853 | -0.7778 |
| C | 3.426479 | 0.026422 | -0.48967 | C | 3.455192 | 0.003572 | -0.4078 |
| C | -2.33795 | -3.56481 | 0.280002 | C | -2.39172 | -3.56412 | 0.013176 |
| H | -2.70248 | -2.73683 | -0.34121 | H | -2.70184 | -2.71875 | -0.61222 |
| H | -2.60473 | -4.48544 | -0.25581 | H | -2.62546 | -4.47323 | -0.55692 |
| C | -0.33407 | -0.0265 | 1.614597 | C | -0.37409 | -0.00517 | 1.613801 |
| C | -0.79845 | -0.04319 | 2.930013 | C | -0.83469 | -0.00859 | 2.940231 |
| H | -1.86229 | -0.05205 | 3.13322 | H | -1.90236 | -0.01034 | 3.122831 |
| C | 0.121977 | -0.04858 | 3.981437 | C | 0.081603 | -0.00958 | 3.978042 |
| C | 1.500485 | -0.03696 | 3.684588 | C | 1.492136 | -0.00703 | 3.684166 |
| C | 1.91905 | -0.02011 | 2.347142 | C | 1.918139 | -0.00376 | 2.365138 |
| H | 2.986936 | -0.01113 | 2.144675 | H | 2.984829 | -0.00191 | 2.164792 |
| C | 1.027498 | -0.01413 | 1.269353 | C | 1.03057 | -0.00268 | 1.269462 |
| C | 1.9097 | -1.99356 | -0.72325 | C | 1.923883 | -2.01524 | -0.68459 |
| C | -4.7652 | -0.05435 | 0.975325 | C | -4.79269 | -0.00722 | 1.06802 |
| H | -5.51388 | -0.07619 | 1.760021 | H | -5.53545 | -0.00953 | 1.857649 |
| C | 3.46558 | -3.64144 | -0.46028 | C | 3.473909 | -3.66754 | -0.45056 |
| H | 4.443183 | -4.06889 | -0.31204 | H | 4.447039 | -4.10341 | -0.29717 |
| C | -0.81561 | -3.46803 | 0.4099 | C | -0.8805 | -3.48958 | 0.251974 |
| H | -0.54272 | -2.57448 | 0.980061 | H | -0.64279 | -2.60977 | 0.857966 |
| H | -0.43312 | -4.32749 | 0.974943 | H | -0.54356 | -4.36481 | 0.822211 |
| C | -0.88467 | 3.435476 | 0.475981 | C | -0.88922 | 3.484588 | 0.264462 |
| H | -0.60212 | 2.537276 | 1.034077 | H | -0.65044 | 2.602838 | 0.867221 |
| H | -0.51937 | 4.291748 | 1.057051 | H | -0.5541 | 4.358191 | 0.838266 |
| C | -2.40746 | 3.511165 | 0.336206 | C | -2.40044 | 3.557463 | 0.025048 |
| H | -2.75425 | 2.689658 | -0.30341 | H | -2.70872 | 2.714174 | -0.60404 |
| H | -2.68434 | 4.437456 | -0.18447 | H | -2.63547 | 4.468523 | -0.54138 |
| C | 6.150638 | 0.044939 | 0.037409 | C | 6.176785 | 0.00573 | 0.100659 |
| H | 7.216706 | 0.052254 | 0.239639 | H | 7.243464 | 0.006586 | 0.298901 |
| C | 5.493727 | -1.18709 | -0.10184 | C | 5.511937 | -1.22371 | -0.02779 |
| H | 6.048297 | -2.11477 | -0.00169 | H | 6.06321 | -2.15334 | 0.071605 |
| C | -4.20544 | -0.02486 | -1.39487 | C | -4.25465 | -0.00047 | -1.34251 |
| H | -4.49522 | -0.02395 | -2.43875 | H | -4.55915 | 0.001159 | -2.37864 |
| C | 5.471314 | 1.267739 | -0.07244 | C | 5.509047 | 1.23411 | -0.02283 |
| H | 6.008822 | 2.202698 | 0.05021 | H | 6.058132 | 2.164612 | 0.080367 |
| C | -3.12191 | 3.439475 | 1.687427 | C | -3.20358 | 3.530131 | 1.327195 |
| H | -2.8868 | 2.503361 | 2.20285 | H | -3.03283 | 2.595349 | 1.869826 |
| H | -4.20838 | 3.488757 | 1.564864 | H | -4.277 | 3.598679 | 1.126555 |
| H | -2.81797 | 4.267692 | 2.336934 | H | -2.92726 | 4.365475 | 1.980909 |
| C | -5.16257 | -0.04283 | -0.36776 | C | -5.19636 | -0.0036 | -0.25134 |
| C | -3.04193 | -3.52965 | 1.638197 | C | -3.19412 | -3.54368 | 1.315904 |
| H | -2.71938 | -4.36502 | 2.26931 | H | -2.91526 | -4.38076 | 1.966315 |
| H | -4.12855 | -3.59381 | 1.524155 | H | -4.26749 | -3.61425 | 1.115689 |
| H | -2.81698 | -2.5998 | 2.169171 | H | -3.02548 | -2.61046 | 1.861872 |
| C | -6.61954 | 0.012853 | -0.71779 | C | -6.64662 | 0.002309 | -0.59079 |
| F | -7.04218 | 1.286236 | -0.90366 | F | -6.99386 | 1.099686 | -1.31737 |
| F | -6.88119 | -0.65301 | -1.86591 | F | -6.993 | -1.06697 | -1.35827 |
| F | -7.39427 | -0.52087 | 0.250481 | F | -7.45461 | -0.01808 | 0.494412 |
| C | 1.697335 | 0.038403 | -2.82438 | C | 1.741975 | 0.006537 | -2.75913 |
| N | 1.85963 | 0.054518 | -3.98484 | N | 1.89621 | 0.009446 | -3.91986 |
| C | 2.521035 | -0.04232 | 4.79576 | C | 2.49104 | -0.00783 | 4.807343 |
| H | 2.410162 | 0.829334 | 5.452597 | H | 2.367203 | 0.869626 | 5.453172 |
| H | 2.420879 | -0.92867 | 5.434381 | H | 2.369341 | -0.88782 | 5.450105 |
| H | 3.537289 | -0.03192 | 4.393311 | H | 3.512548 | -0.0059 | 4.421524 |
| C | -0.36533 | -0.06679 | 5.408974 | C | -0.39444 | -0.01336 | 5.402894 |
| H | -0.00333 | -0.9496 | 5.950182 | H | -0.02488 | -0.89298 | 5.943207 |
| H | -0.01381 | 0.808439 | 5.969076 | H | -0.02673 | 0.864507 | 5.9473 |
| H | -1.45763 | -0.07376 | 5.450855 | H | -1.48479 | -0.01464 | 5.451887 |

**Table S7**. Cartesian coordinates for optimized structures of **CF_3_-2**.

| Ground state of **CF_3_-2** (S_0_) | | | | Excited state of **CF_3_-2** (T_1_) | | | |
| --- | --- | --- | --- | --- | --- | --- | --- |
| Ir | -1.4879 | -0.00625 | -0.52789 | Ir | -1.52127 | -0.00076 | -0.45902 |
| N | -1.33141 | -3.22433 | -0.5201 | N | -1.38718 | -3.22377 | -0.5789 |
| N | -3.25863 | -2.31633 | -0.17325 | N | -3.29891 | -2.30542 | -0.15649 |
| N | -3.29186 | 2.281919 | -0.19867 | N | -3.30265 | 2.301481 | -0.15993 |
| N | -1.37735 | 3.213767 | -0.55227 | N | -1.39234 | 3.222304 | -0.58334 |
| C | -2.2442 | -4.25875 | -0.31967 | C | -2.31066 | -4.25105 | -0.41437 |
| H | -1.9487 | -5.29466 | -0.35679 | H | -2.03257 | -5.28842 | -0.50635 |
| N | 1.589191 | 0.011142 | -1.48326 | N | 1.626025 | 0.000143 | -1.4338 |
| N | 1.181215 | 0.018001 | 0.674682 | N | 1.201547 | 0.002654 | 0.711947 |
| N | 3.481814 | 0.042473 | 1.500137 | N | 3.485505 | 0.005405 | 1.603933 |
| C | -4.15284 | -1.22104 | -0.02642 | C | -4.18378 | -1.20359 | 0.022275 |
| C | 0.106416 | -3.40382 | -0.70257 | C | 0.044501 | -3.40255 | -0.83087 |
| H | 0.453287 | -2.57037 | -1.31506 | H | 0.365979 | -2.54808 | -1.4275 |
| H | 0.260728 | -4.32532 | -1.27159 | H | 0.164938 | -4.30598 | -1.435 |
| C | -1.93457 | -2.0182 | -0.42567 | C | -1.97203 | -2.01925 | -0.41635 |
| C | -4.17026 | 1.175552 | -0.04004 | C | -4.18574 | 1.198492 | 0.020447 |
| C | 0.56442 | 0.007927 | -0.57156 | C | 0.572428 | 0.000721 | -0.56439 |
| C | 0.058657 | 3.411766 | -0.72956 | C | 0.039143 | 3.402997 | -0.83496 |
| H | 0.202953 | 4.330977 | -1.30486 | H | 0.158424 | 4.305622 | -1.44052 |
| H | 0.420337 | 2.578515 | -1.33374 | H | 0.362296 | 2.548039 | -1.42999 |
| C | -3.45783 | -3.68611 | -0.10686 | C | -3.51483 | -3.67251 | -0.15235 |
| H | -4.42462 | -4.12667 | 0.071012 | H | -4.48376 | -4.11025 | 0.020986 |
| C | 1.450153 | 0.003866 | -2.93411 | C | 1.521076 | -0.00187 | -2.88277 |
| H | 1.934717 | -0.88609 | -3.34604 | H | 2.015821 | -0.89059 | -3.28757 |
| H | 1.923907 | 0.896039 | -3.35375 | H | 2.015478 | 0.885925 | -3.29 |
| H | 0.39265 | -0.00367 | -3.18844 | H | 0.471124 | -0.00244 | -3.16694 |
| C | 2.819424 | 0.020353 | -0.84196 | C | 2.85539 | 0.001547 | -0.77701 |
| C | 2.558177 | 0.026403 | 0.547929 | C | 2.58918 | 0.003379 | 0.597904 |
| C | -2.30486 | 4.237058 | -0.36253 | C | -2.31744 | 4.248346 | -0.42012 |
| H | -2.02375 | 5.276605 | -0.4089 | H | -2.04094 | 5.286029 | -0.51331 |
| C | -3.47757 | -0.01864 | -0.18492 | C | -3.50262 | -0.00209 | -0.11162 |
| C | 2.315137 | 3.566069 | 0.430365 | C | 2.355643 | 3.563987 | 0.184291 |
| H | 2.65836 | 2.737249 | -0.20181 | H | 2.651366 | 2.719213 | -0.44894 |
| H | 2.565977 | 4.485956 | -0.1143 | H | 2.575953 | 4.473497 | -0.39044 |
| C | 0.341021 | 0.017763 | 1.825871 | C | 0.380973 | 0.002967 | 1.817724 |
| C | 0.849799 | 0.027046 | 3.125845 | C | 0.883586 | 0.004603 | 3.133083 |
| H | 1.918383 | 0.03476 | 3.297554 | H | 1.954796 | 0.005709 | 3.289076 |
| C | -0.05741 | 0.026245 | 4.186586 | C | -0.02368 | 0.004669 | 4.17499 |
| C | -1.42952 | 0.016273 | 3.928859 | C | -1.41968 | 0.003135 | 3.922008 |
| H | -2.134 | 0.01567 | 4.756597 | H | -2.10964 | 0.003213 | 4.7608 |
| C | -1.90407 | 0.00689 | 2.612866 | C | -1.90215 | 0.00154 | 2.626184 |
| H | -2.97568 | -0.00102 | 2.436482 | H | -2.97256 | 0.000369 | 2.451478 |
| C | -1.03336 | 0.007269 | 1.515796 | C | -1.03233 | 0.001353 | 1.510357 |
| C | -1.96342 | 2.000164 | -0.44686 | C | -1.97531 | 2.017067 | -0.41934 |
| C | 4.751606 | 0.049478 | 1.063842 | C | 4.782043 | 0.004975 | 1.149804 |
| H | 5.521446 | 0.068677 | 1.827826 | H | 5.54586 | 0.006279 | 1.918974 |
| C | -3.51051 | 3.649302 | -0.14554 | C | -3.52072 | 3.66823 | -0.15746 |
| H | -4.48363 | 4.077685 | 0.02734 | H | -4.49034 | 4.104635 | 0.01532 |
| C | 0.797405 | 3.471907 | 0.608084 | C | 0.850217 | 3.488955 | 0.457695 |
| H | 0.540826 | 2.579236 | 1.187149 | H | 0.62617 | 2.608267 | 1.067572 |
| H | 0.433947 | 4.332166 | 1.184259 | H | 0.52636 | 4.363198 | 1.036755 |
| C | 0.853855 | -3.44345 | 0.631026 | C | 0.856419 | -3.48508 | 0.461475 |
| H | 0.588637 | -2.55015 | 1.205247 | H | 0.631598 | -2.60358 | 1.069895 |
| H | 0.506531 | -4.30449 | 1.215932 | H | 0.534047 | -4.35871 | 1.042291 |
| C | 2.371577 | -3.51722 | 0.443156 | C | 2.361794 | -3.55858 | 0.187331 |
| H | 2.697933 | -2.68943 | -0.19916 | H | 2.656019 | -2.71462 | -0.44766 |
| H | 2.631887 | -4.43826 | -0.09503 | H | 2.583044 | -4.46888 | -0.38578 |
| C | -6.19084 | -0.03503 | 0.394053 | C | -6.21602 | -0.00388 | 0.433399 |
| H | -7.25277 | -0.04149 | 0.616914 | H | -7.27965 | -0.00459 | 0.647003 |
| C | -5.53437 | 1.196572 | 0.249289 | C | -5.55283 | 1.225514 | 0.296962 |
| H | -6.08527 | 2.124648 | 0.364664 | H | -6.10254 | 2.155065 | 0.404692 |
| C | 4.126674 | 0.028945 | -1.29001 | C | 4.178871 | 0.001253 | -1.24542 |
| H | 4.387528 | 0.031731 | -2.34146 | H | 4.455477 | 0.000605 | -2.28934 |
| C | -5.51649 | -1.25852 | 0.263405 | C | -5.55081 | -1.2324 | 0.298863 |
| H | -6.05376 | -2.19311 | 0.389687 | H | -6.099 | -2.16268 | 0.408058 |
| C | 3.128606 | -3.45871 | 1.771624 | C | 3.194619 | -3.53416 | 1.470681 |
| H | 2.909539 | -2.52839 | 2.304345 | H | 3.037083 | -2.60053 | 2.019215 |
| H | 4.210638 | -3.50561 | 1.613928 | H | 4.263054 | -3.60317 | 1.245189 |
| H | 2.84613 | -4.29382 | 2.421919 | H | 2.9328 | -4.37043 | 2.129017 |
| C | 5.111655 | 0.043059 | -0.28964 | C | 5.149663 | 0.002983 | -0.18083 |
| C | 3.062385 | 3.530825 | 1.765204 | C | 3.187753 | 3.543172 | 1.468167 |
| H | 2.761012 | 4.366556 | 2.406147 | H | 2.923908 | 4.379944 | 2.125049 |
| H | 4.144793 | 3.594175 | 1.616063 | H | 4.256189 | 3.613933 | 1.243218 |
| H | 2.854216 | 2.601388 | 2.303724 | H | 3.031744 | 2.610051 | 2.018 |
| C | 6.559053 | -0.01109 | -0.67869 | C | 6.590579 | -0.00143 | -0.56018 |
| F | 6.979767 | -1.28451 | -0.86728 | F | 6.916233 | -1.09543 | -1.30096 |
| F | 6.787541 | 0.648161 | -1.83745 | F | 6.914759 | 1.071565 | -1.33151 |
| F | 7.357625 | 0.530935 | 0.265175 | F | 7.427296 | 0.013797 | 0.502615 |
| C | -1.80975 | -0.01965 | -2.55863 | C | -1.8558 | -0.00263 | -2.49765 |
| N | -2.00108 | -0.02748 | -3.71464 | N | -2.04557 | -0.0037 | -3.65306 |
| H | 0.311639 | 0.03338 | 5.207649 | H | 0.335206 | 0.005892 | 5.199395 |

**Table S8**. Cartesian coordinates for optimized structures of **CF_3_-3**.

| Ground state of **CF_3_-3** (S_0_) | | | | Excited state of **CF_3_-3** (T_1_) | | | |
| --- | --- | --- | --- | --- | --- | --- | --- |
| Ir | -1.42657 | -0.00691 | -0.66199 | Ir | -1.46141 | -0.00102 | -0.61213 |
| N | -1.2673 | -3.22568 | -0.6559 | N | -1.32409 | -3.22514 | -0.70019 |
| N | -3.20258 | -2.3196 | -0.35183 | N | -3.24452 | -2.30577 | -0.32645 |
| N | -3.24089 | 2.279312 | -0.37881 | N | -3.24851 | 2.301234 | -0.3314 |
| N | -1.32048 | 3.213688 | -0.69119 | N | -1.32962 | 3.22314 | -0.70675 |
| C | -2.18361 | -4.26106 | -0.47749 | C | -2.25053 | -4.2528 | -0.54771 |
| H | -1.88651 | -5.29669 | -0.50913 | H | -1.96949 | -5.29039 | -0.62716 |
| N | 1.679342 | 0.013866 | -1.52926 | N | 1.70296 | -9.3E-05 | -1.4856 |
| N | 1.207118 | 0.019608 | 0.615006 | N | 1.219078 | 0.002753 | 0.649908 |
| N | 3.480145 | 0.043697 | 1.510566 | N | 3.473816 | 0.005788 | 1.609678 |
| C | -4.10126 | -1.2251 | -0.22533 | C | -4.13337 | -1.20365 | -0.17176 |
| C | 0.174236 | -3.40381 | -0.80901 | C | 0.111146 | -3.40619 | -0.92584 |
| H | 0.532843 | -2.56907 | -1.41284 | H | 0.444467 | -2.55383 | -1.51895 |
| H | 0.340697 | -4.32411 | -1.37642 | H | 0.241722 | -4.31074 | -1.52622 |
| C | -1.8736 | -2.02052 | -0.57374 | C | -1.91349 | -2.01966 | -0.56065 |
| C | -4.12132 | 1.171605 | -0.23968 | C | -4.13546 | 1.197932 | -0.17438 |
| C | 0.628068 | 0.009591 | -0.64861 | C | 0.623859 | 0.000536 | -0.65128 |
| C | 0.118576 | 3.413441 | -0.84049 | C | 0.105394 | 3.406184 | -0.93211 |
| H | 0.272588 | 4.332168 | -1.4139 | H | 0.234734 | 4.309704 | -1.5343 |
| H | 0.492988 | 2.580086 | -1.43666 | H | 0.440477 | 2.553141 | -1.52327 |
| C | -3.40219 | -3.68955 | -0.29142 | C | -3.45979 | -3.67356 | -0.31587 |
| H | -4.37237 | -4.13102 | -0.13566 | H | -4.43206 | -4.11034 | -0.15977 |
| C | 1.582459 | 0.006989 | -2.98362 | C | 1.641767 | -0.00228 | -2.93781 |
| H | 2.07761 | -0.88338 | -3.38174 | H | 2.148537 | -0.89125 | -3.3264 |
| H | 2.068662 | 0.898896 | -3.38926 | H | 2.148363 | 0.885639 | -3.32903 |
| H | 0.532645 | 0.000309 | -3.26783 | H | 0.600545 | -0.00283 | -3.25268 |
| C | 2.889938 | 0.022983 | -0.8514 | C | 2.91289 | 0.00151 | -0.79186 |
| C | 2.586888 | 0.028318 | 0.529868 | C | 2.606744 | 0.003554 | 0.573942 |
| C | -2.25374 | 4.235798 | -0.52458 | C | -2.2578 | 4.24953 | -0.55629 |
| H | -1.97333 | 5.275721 | -0.56677 | H | -1.97848 | 5.287441 | -0.63767 |
| C | -3.42466 | -0.02183 | -0.37005 | C | -3.45173 | -0.00241 | -0.30669 |
| C | 2.351969 | 3.574248 | 0.362468 | C | 2.403812 | 3.568636 | 0.1289 |
| H | 2.709934 | 2.743574 | -0.25899 | H | 2.711776 | 2.7251 | -0.50024 |
| H | 2.610449 | 4.492381 | -0.18149 | H | 2.633948 | 4.478957 | -0.44052 |
| C | 0.334684 | 0.018773 | 1.740776 | C | 0.368612 | 0.003312 | 1.728861 |
| C | 0.802945 | 0.028779 | 3.055013 | C | 0.84214 | 0.005295 | 3.060827 |
| H | 1.865334 | 0.037427 | 3.261289 | H | 1.909833 | 0.006455 | 3.241074 |
| C | -0.12907 | 0.027554 | 4.093995 | C | -0.08205 | 0.005634 | 4.082229 |
| C | -1.47964 | 0.016394 | 3.767676 | C | -1.45766 | 0.004 | 3.758762 |
| C | -1.94046 | 0.006305 | 2.454841 | C | -1.93418 | 0.002057 | 2.462325 |
| H | -3.0102 | -0.00229 | 2.275358 | H | -3.00451 | 0.000875 | 2.292387 |
| C | -1.03203 | 0.007167 | 1.390699 | C | -1.03498 | 0.001619 | 1.383058 |
| C | -1.90693 | 1.999715 | -0.59635 | C | -1.91697 | 2.016933 | -0.56495 |
| C | 4.762907 | 0.050221 | 1.113627 | C | 4.781067 | 0.005295 | 1.193279 |
| H | 5.50896 | 0.068412 | 1.900805 | H | 5.523228 | 0.006707 | 1.983278 |
| C | -3.46298 | 3.64638 | -0.33336 | C | -3.4661 | 3.668672 | -0.32344 |
| H | -4.44041 | 4.073423 | -0.18296 | H | -4.43911 | 4.104086 | -0.1682 |
| C | 0.831294 | 3.47648 | 0.511077 | C | 0.893663 | 3.492503 | 0.374624 |
| H | 0.566021 | 2.583548 | 1.085913 | H | 0.65885 | 2.611983 | 0.98084 |
| H | 0.454742 | 4.336252 | 1.079417 | H | 0.55893 | 4.366293 | 0.948053 |
| C | 0.894534 | -3.44584 | 0.539383 | C | 0.900313 | -3.48849 | 0.380622 |
| H | 0.618612 | -2.55304 | 1.109452 | H | 0.664687 | -2.60696 | 0.985052 |
| H | 0.534915 | -4.30741 | 1.115953 | H | 0.56716 | -4.36149 | 0.956177 |
| C | 2.415731 | -3.5206 | 0.381975 | C | 2.410421 | -3.56302 | 0.134098 |
| H | 2.75614 | -2.69024 | -0.24961 | H | 2.716778 | -2.72051 | -0.49718 |
| H | 2.685505 | -4.43945 | -0.15521 | H | 2.641531 | -4.47432 | -0.43335 |
| C | -6.14903 | -0.04149 | 0.151247 | C | -6.16938 | -0.0042 | 0.219943 |
| H | -7.21524 | -0.04922 | 0.352291 | H | -7.23466 | -0.0049 | 0.425142 |
| C | -5.49131 | 1.190921 | 0.020448 | C | -5.50464 | 1.225046 | 0.091109 |
| H | -6.04549 | 2.118228 | 0.125083 | H | -6.054 | 2.154725 | 0.199168 |
| C | 4.210439 | 0.031546 | -1.2587 | C | 4.249501 | 0.001222 | -1.22237 |
| H | 4.503845 | 0.03509 | -2.30153 | H | 4.55725 | 0.000493 | -2.25728 |
| C | -5.47072 | -1.26418 | 0.03527 | C | -5.50249 | -1.23256 | 0.093821 |
| H | -6.00924 | -2.1993 | 0.151319 | H | -6.05022 | -2.16295 | 0.203966 |
| C | 3.146182 | -3.47004 | 1.725575 | C | 3.219977 | -3.53711 | 1.432253 |
| H | 2.918061 | -2.54227 | 2.259015 | H | 3.050149 | -2.60429 | 1.978716 |
| H | 4.231037 | -3.51756 | 1.589043 | H | 4.292508 | -3.60341 | 1.226295 |
| H | 2.84994 | -4.30815 | 2.365724 | H | 2.948302 | -4.37421 | 2.085403 |
| C | 5.164013 | 0.044392 | -0.22832 | C | 5.18707 | 0.003084 | -0.12767 |
| C | 3.07358 | 3.548095 | 1.711564 | C | 3.212542 | 3.546935 | 1.427648 |
| H | 2.757646 | 4.386167 | 2.342287 | H | 2.938672 | 4.384746 | 2.078972 |
| H | 4.158407 | 3.614047 | 1.582712 | H | 4.285077 | 3.615094 | 1.222313 |
| H | 2.858277 | 2.620888 | 2.251223 | H | 3.044321 | 2.614804 | 1.975774 |
| C | 6.622472 | -0.01085 | -0.57402 | C | 6.639554 | -0.00138 | -0.46372 |
| F | 7.044396 | -1.28403 | -0.76077 | F | 6.985179 | -1.09563 | -1.19378 |
| F | 6.8872 | 0.657211 | -1.71989 | F | 6.984721 | 1.071754 | -1.22449 |
| F | 7.393682 | 0.520855 | 0.397807 | F | 7.443352 | 0.013895 | 0.623729 |
| C | -1.69001 | -0.02073 | -2.69921 | C | -1.73485 | -0.00361 | -2.65152 |
| N | -1.84875 | -0.02921 | -3.86001 | N | -1.8871 | -0.00522 | -3.81261 |
| H | 0.179911 | 0.035053 | 5.13279 | H | 0.216757 | 0.007099 | 5.124091 |
| F | -2.38791 | 0.015219 | 4.776458 | F | -2.33011 | 0.004381 | 4.781287 |

**Table S9**. Cartesian coordinates for optimized structures of **F-1**.

| Ground state of **F-1** (S_0_) | | | | Excited state of **F-1** (T_1_) | | | |
| --- | --- | --- | --- | --- | --- | --- | --- |
| Ir | -1.06419 | -3E-06 | -0.74799 | Ir | 1.080649 | 0.000013 | -0.71228 |
| N | -0.92593 | -3.21862 | -0.75069 | N | 0.946383 | 3.224873 | -0.77893 |
| N | -2.82306 | -2.29893 | -0.28767 | N | 2.836051 | 2.303079 | -0.27971 |
| N | -2.82304 | 2.29894 | -0.28769 | N | 2.836065 | -2.30306 | -0.27979 |
| N | -0.9259 | 3.218611 | -0.75072 | N | 0.946403 | -3.22485 | -0.77904 |
| C | -1.83019 | -4.2474 | -0.49095 | C | 1.857201 | 4.252497 | -0.54516 |
| H | -1.54339 | -5.28509 | -0.54357 | H | 1.580192 | 5.290706 | -0.63054 |
| N | 1.955518 | -2.1E-05 | -1.89525 | N | -2.00675 | 0.00003 | -1.83109 |
| N | 1.677884 | -3E-06 | 0.281871 | N | -1.69405 | -1.2E-05 | 0.345977 |
| N | 4.033082 | -4E-06 | 0.959136 | N | -4.01373 | -0.00003 | 1.10745 |
| C | -3.69835 | -1.19838 | -0.08011 | C | 3.710734 | 1.200646 | -0.06256 |
| C | 0.499437 | -3.40664 | -1.00704 | C | -0.47275 | 3.406843 | -1.08549 |
| H | 0.819966 | -2.57626 | -1.63791 | H | -0.77243 | 2.555929 | -1.69857 |
| H | 0.619247 | -4.33063 | -1.58057 | H | -0.57088 | 4.314401 | -1.688 |
| C | -1.51506 | -2.00831 | -0.62239 | C | 1.526142 | 2.01723 | -0.61394 |
| C | -3.69834 | 1.198396 | -0.08012 | C | 3.710741 | -1.20063 | -0.06261 |
| C | 0.988167 | -0.00001 | -0.91819 | C | -0.99179 | 0.000013 | -0.91478 |
| C | 0.499459 | 3.406618 | -1.00708 | C | -0.47273 | -3.40681 | -1.0856 |
| H | 0.619272 | 4.330597 | -1.58063 | H | -0.57086 | -4.31435 | -1.68814 |
| H | 0.81998 | 2.576228 | -1.63793 | H | -0.77241 | -2.55588 | -1.69866 |
| C | -3.02518 | -3.66738 | -0.20516 | C | 3.047206 | 3.67159 | -0.23507 |
| H | -3.98152 | -4.10193 | 0.033507 | H | 4.005137 | 4.106789 | -0.00387 |
| C | 1.724198 | -0.00002 | -3.33351 | C | -1.82672 | 0.000059 | -3.27398 |
| H | 2.175591 | -0.89086 | -3.78019 | H | -2.29781 | 0.888964 | -3.70514 |
| H | 2.175519 | 0.890858 | -3.78018 | H | -2.29781 | -0.88883 | -3.70518 |
| H | 0.652585 | -0.00006 | -3.52011 | H | -0.76277 | 0.000066 | -3.50112 |
| C | 3.221864 | -1.8E-05 | -1.3328 | C | -3.25918 | 0.000018 | -1.23716 |
| C | 3.050454 | -8E-06 | 0.069488 | C | -3.06972 | -0.00001 | 0.159093 |
| C | -1.83015 | 4.247403 | -0.49099 | C | 1.857228 | -4.25247 | -0.5453 |
| H | -1.54335 | 5.28509 | -0.54361 | H | 1.580225 | -5.29068 | -0.63072 |
| C | -3.02583 | 0.000005 | -0.27779 | C | 3.039704 | 0.000012 | -0.251 |
| C | 2.817735 | 3.534664 | 0.02838 | C | -2.82706 | -3.56086 | -0.15013 |
| H | 3.119936 | 2.711683 | -0.63158 | H | -3.09981 | -2.72206 | -0.8017 |
| H | 3.044863 | 4.46043 | -0.51697 | H | -3.02683 | -4.47566 | -0.72416 |
| C | 0.91059 | 0.000007 | 1.481756 | C | -0.93034 | -3.2E-05 | 1.485893 |
| C | 1.491883 | 0.000018 | 2.749785 | C | -1.49349 | -5.8E-05 | 2.777879 |
| H | 2.569901 | 0.000018 | 2.855503 | H | -2.57161 | -6.4E-05 | 2.879062 |
| C | 0.670634 | 0.000029 | 3.880855 | C | -0.66153 | -7.7E-05 | 3.881782 |
| C | -0.72891 | 0.000028 | 3.710862 | C | 0.762957 | -6.8E-05 | 3.693192 |
| C | -1.2672 | 0.000017 | 2.416566 | C | 1.29017 | -4.2E-05 | 2.404955 |
| H | -2.34917 | 0.000017 | 2.311305 | H | 2.369656 | -3.6E-05 | 2.28953 |
| C | -0.47724 | 0.000007 | 1.262455 | C | 0.492117 | -2.3E-05 | 1.250298 |
| C | -1.51504 | 2.008306 | -0.62241 | C | 1.526155 | -2.0172 | -0.61401 |
| C | 5.275982 | -9E-06 | 0.448811 | C | -5.29644 | -2.1E-05 | 0.591432 |
| H | 6.108214 | -5E-06 | 1.145504 | H | -6.11941 | -3.6E-05 | 1.296657 |
| C | -3.02515 | 3.667389 | -0.20519 | C | 3.047229 | -3.67157 | -0.2352 |
| H | -3.98149 | 4.10195 | 0.033479 | H | 4.005163 | -4.10677 | -0.00401 |
| C | 1.311663 | 3.446956 | 0.288062 | C | -1.33264 | -3.4819 | 0.176057 |
| H | 1.082477 | 2.548459 | 0.869375 | H | -1.13106 | -2.59578 | 0.785877 |
| H | 0.985717 | 4.302391 | 0.893335 | H | -1.03022 | -4.35078 | 0.774639 |
| C | 1.311631 | -3.44696 | 0.288108 | C | -1.33266 | 3.48189 | 0.176175 |
| H | 1.082446 | -2.54845 | 0.869405 | H | -1.13108 | 2.595761 | 0.785973 |
| H | 0.985675 | -4.30238 | 0.893393 | H | -1.03024 | 4.350759 | 0.774779 |
| C | 2.817704 | -3.53468 | 0.028439 | C | -2.82708 | 3.560857 | -0.15001 |
| H | 3.119915 | -2.71171 | -0.63154 | H | -3.09983 | 2.722065 | -0.80159 |
| H | 3.044831 | -4.46046 | -0.51689 | H | -3.02685 | 4.475664 | -0.72402 |
| C | -5.69525 | 0.000018 | 0.480204 | C | 5.709425 | 0.000007 | 0.487418 |
| H | -6.74025 | 0.000023 | 0.772593 | H | 6.756013 | 0.000006 | 0.773703 |
| C | -5.04014 | 1.22764 | 0.299081 | C | 5.054918 | -1.22877 | 0.30898 |
| H | -5.57491 | 2.159121 | 0.456081 | H | 5.593076 | -2.15918 | 0.460449 |
| C | 4.500878 | -2.1E-05 | -1.87015 | C | -4.56545 | 0.000028 | -1.77553 |
| H | 4.711214 | -2.7E-05 | -2.93276 | H | -4.80601 | 0.000049 | -2.82813 |
| C | -5.04014 | -1.22761 | 0.299092 | C | 5.054911 | 1.228788 | 0.309022 |
| H | -5.57492 | -2.15909 | 0.456099 | H | 5.593063 | 2.15919 | 0.460522 |
| C | 3.635651 | -3.47623 | 1.320332 | C | -3.70367 | 3.528321 | 1.103707 |
| H | 3.451859 | -2.53911 | 1.854289 | H | -3.5672 | 2.588989 | 1.64804 |
| H | 4.708842 | -3.53723 | 1.114234 | H | -4.76394 | 3.603179 | 0.843769 |
| H | 3.373283 | -4.30334 | 1.989192 | H | -3.46117 | 4.357823 | 1.77788 |
| C | 5.526543 | -1.7E-05 | -0.92487 | C | -5.55575 | 0.000006 | -0.76827 |
| C | 3.635691 | 3.476227 | 1.320268 | C | -3.70365 | -3.52835 | 1.103586 |
| H | 3.373339 | 4.303359 | 1.98911 | H | -3.46115 | -4.35786 | 1.777744 |
| H | 4.708881 | 3.53721 | 1.11416 | H | -4.76392 | -3.6032 | 0.843645 |
| H | 3.45189 | 2.539126 | 1.854248 | H | -3.56718 | -2.58903 | 1.647936 |
| C | -1.50846 | -1.7E-05 | -2.75559 | C | 1.513395 | 0.000045 | -2.72496 |
| N | -1.76557 | -2.8E-05 | -3.89902 | N | 1.752268 | 0.000063 | -3.87168 |
| C | -1.64441 | 0.000039 | 4.910185 | C | 1.677247 | -8.8E-05 | 4.88622 |
| H | -1.48097 | -0.87902 | 5.545932 | H | 1.507407 | 0.878394 | 5.520465 |
| H | -1.48097 | 0.879112 | 5.545917 | H | 1.507413 | -0.8786 | 5.520431 |
| H | -2.69303 | 0.000037 | 4.601392 | H | 2.724677 | -7.8E-05 | 4.577179 |
| C | 1.285916 | 0.000041 | 5.258285 | C | -1.24432 | -0.00011 | 5.268223 |
| H | 0.980896 | 0.8791 | 5.839358 | H | -0.92035 | -0.87915 | 5.837968 |
| H | 0.980897 | -0.87901 | 5.839374 | H | -0.92036 | 0.878912 | 5.838003 |
| H | 2.377563 | 0.000041 | 5.200608 | H | -2.33555 | -0.00011 | 5.233435 |
| F | 6.806024 | -1.9E-05 | -1.34862 | F | -6.84832 | 0.000013 | -1.16664 |

**Table S10**. Cartesian coordinates for optimized structures of **F-2**.

| Ground state of **F-2** (S_0_) | | | | Excited state of **F-2** (T_1_) | | | |
| --- | --- | --- | --- | --- | --- | --- | --- |
| Ir | -1.07458 | 0.000003 | -0.51115 | Ir | 1.08558 | -2E-06 | -0.50115 |
| N | -0.93983 | -3.21875 | -0.52714 | N | 0.949758 | 3.221697 | -0.52394 |
| N | -2.83329 | -2.2991 | -0.04911 | N | 2.842965 | 2.30187 | -0.03622 |
| N | -2.83332 | 2.29908 | -0.04909 | N | 2.842964 | -2.30187 | -0.03621 |
| N | -0.93988 | 3.218759 | -0.52712 | N | 0.949758 | -3.2217 | -0.52393 |
| C | -1.84434 | -4.24775 | -0.26923 | C | 1.857139 | 4.250108 | -0.27839 |
| H | -1.55933 | -5.28552 | -0.32917 | H | 1.575753 | 5.287976 | -0.3521 |
| N | 1.93695 | 0.000025 | -1.67268 | N | -1.96619 | -8E-06 | -1.63825 |
| N | 1.671184 | 0.000002 | 0.505754 | N | -1.68063 | 0.000002 | 0.547862 |
| N | 4.030686 | -3E-06 | 1.169757 | N | -3.99857 | 0.000008 | 1.292119 |
| C | -3.70768 | -1.19846 | 0.162668 | C | 3.718219 | 1.199548 | 0.174812 |
| C | 0.482491 | -3.40722 | -0.79998 | C | -0.46738 | 3.406968 | -0.83716 |
| H | 0.796632 | -2.57543 | -1.43221 | H | -0.76423 | 2.562834 | -1.46095 |
| H | 0.594557 | -4.32949 | -1.37773 | H | -0.56031 | 4.319409 | -1.43308 |
| C | -1.52667 | -2.00865 | -0.38845 | C | 1.536007 | 2.015225 | -0.37351 |
| C | -3.7077 | 1.198424 | 0.162678 | C | 3.718218 | -1.19955 | 0.17482 |
| C | 0.975075 | 0.000015 | -0.69099 | C | -0.96142 | -3E-06 | -0.70659 |
| C | 0.482444 | 3.407244 | -0.79994 | C | -0.46738 | -3.40697 | -0.83715 |
| H | 0.594504 | 4.32953 | -1.37767 | H | -0.56031 | -4.31942 | -1.43306 |
| H | 0.796597 | 2.575476 | -1.43219 | H | -0.76423 | -2.56284 | -1.46095 |
| C | -3.03702 | -3.66772 | 0.026107 | C | 3.049018 | 3.670549 | 0.025365 |
| H | -3.9927 | -4.10231 | 0.267322 | H | 4.004893 | 4.106195 | 0.263838 |
| C | 1.698652 | 0.000068 | -3.10992 | C | -1.77107 | -1.6E-05 | -3.08084 |
| H | 2.147988 | -0.89077 | -3.55859 | H | -2.23734 | 0.889357 | -3.5152 |
| H | 2.147866 | 0.890998 | -3.55852 | H | -2.23734 | -0.8894 | -3.51519 |
| H | 0.626211 | 0.000003 | -3.29145 | H | -0.70501 | -1.6E-05 | -3.29666 |
| C | 3.206615 | 0.000024 | -1.11702 | C | -3.21812 | -4E-06 | -1.05783 |
| C | 3.04325 | 0.000006 | 0.285802 | C | -3.03866 | 0.000003 | 0.347606 |
| C | -1.8444 | 4.247741 | -0.2692 | C | 1.857136 | -4.25011 | -0.27836 |
| H | -1.55941 | 5.285513 | -0.32914 | H | 1.57575 | -5.28798 | -0.35207 |
| C | -3.03539 | -1.3E-05 | -0.03477 | C | 3.049872 | -1E-06 | -0.02594 |
| C | 2.813178 | 3.540537 | 0.204332 | C | -2.82755 | -3.56329 | 0.080221 |
| H | 3.107367 | 2.714119 | -0.45501 | H | -3.09896 | -2.73151 | -0.58142 |
| H | 3.031492 | 4.463326 | -0.34956 | H | -3.01775 | -4.48325 | -0.48871 |
| C | 0.912524 | -1.3E-05 | 1.711064 | C | -0.91959 | 0.000007 | 1.707729 |
| C | 1.509216 | -2.8E-05 | 2.973015 | C | -1.50174 | 0.000012 | 2.990933 |
| H | 2.587256 | -0.00003 | 3.07046 | H | -2.5798 | 0.000013 | 3.085302 |
| C | 0.677335 | -0.00004 | 4.094233 | C | -0.65899 | 0.000015 | 4.08895 |
| C | -0.70917 | -3.6E-05 | 3.932294 | C | 0.738762 | 0.000013 | 3.90964 |
| H | -1.35481 | -4.5E-05 | 4.806759 | H | 1.387741 | 0.000016 | 4.781125 |
| C | -1.27339 | -2.1E-05 | 2.65181 | C | 1.294745 | 0.000008 | 2.632431 |
| H | -2.35469 | -1.9E-05 | 2.549407 | H | 2.374265 | 0.000007 | 2.522512 |
| C | -0.48062 | -0.00001 | 1.497433 | C | 0.492685 | 0.000005 | 1.481024 |
| C | -1.52669 | 2.008645 | -0.38843 | C | 1.536006 | -2.01523 | -0.37349 |
| C | 5.270874 | 0.000007 | 0.65268 | C | -5.26686 | 0.000005 | 0.755717 |
| H | 6.106818 | -1E-06 | 1.344841 | H | -6.10403 | 0.000009 | 1.444269 |
| C | -3.03707 | 3.667691 | 0.026136 | C | 3.049016 | -3.67055 | 0.025387 |
| H | -3.99276 | 4.102265 | 0.267355 | H | 4.004891 | -4.1062 | 0.263864 |
| C | 1.31075 | 3.452655 | 0.484683 | C | -1.33629 | -3.47425 | 0.41867 |
| H | 1.090103 | 2.556046 | 1.072135 | H | -1.14268 | -2.58365 | 1.024386 |
| H | 0.992086 | 4.30973 | 1.091361 | H | -1.03559 | -4.33744 | 1.02599 |
| C | 1.310802 | -3.45265 | 0.484641 | C | -1.33628 | 3.474254 | 0.418661 |
| H | 1.090156 | -2.55606 | 1.072114 | H | -1.14267 | 2.583652 | 1.02438 |
| H | 0.992143 | -4.30974 | 1.091301 | H | -1.03558 | 4.337447 | 1.025977 |
| C | 2.813229 | -3.54053 | 0.20428 | C | -2.82754 | 3.563294 | 0.080217 |
| H | 3.107413 | -2.71409 | -0.45504 | H | -3.09896 | 2.73152 | -0.58142 |
| H | 3.031541 | -4.4633 | -0.34963 | H | -3.01775 | 4.483254 | -0.48871 |
| C | -5.7041 | -3.5E-05 | 0.724279 | C | 5.712294 | 0.000001 | 0.741814 |
| H | -6.74886 | -4.4E-05 | 1.01741 | H | 6.755946 | 0.000001 | 1.038417 |
| C | -5.04929 | 1.22765 | 0.542574 | C | 5.058578 | -1.22824 | 0.559478 |
| H | -5.58421 | 2.159072 | 0.699249 | H | 5.59304 | -2.15903 | 0.720669 |
| C | 4.482458 | 0.000036 | -1.66174 | C | -4.51965 | -7E-06 | -1.61518 |
| H | 4.686737 | 0.000053 | -2.72551 | H | -4.74834 | -1.3E-05 | -2.66986 |
| C | -5.04928 | -1.22771 | 0.542563 | C | 5.058579 | 1.228242 | 0.559471 |
| H | -5.58418 | -2.15914 | 0.699231 | H | 5.593041 | 2.159029 | 0.720656 |
| C | 3.64923 | -3.49121 | 1.484916 | C | -3.71517 | 3.525994 | 1.326006 |
| H | 3.473893 | -2.55777 | 2.028128 | H | -3.58701 | 2.583002 | 1.865935 |
| H | 4.719335 | -3.55152 | 1.263204 | H | -4.77288 | 3.607938 | 1.057509 |
| H | 3.395795 | -4.32259 | 2.151842 | H | -3.47469 | 4.349994 | 2.007344 |
| C | 5.513565 | 0.000027 | -0.72236 | C | -5.51295 | -2E-06 | -0.61597 |
| C | 3.649172 | 3.491193 | 1.484971 | C | -3.71518 | -3.52598 | 1.326006 |
| H | 3.395732 | 4.322555 | 2.151916 | H | -3.4747 | -4.34998 | 2.007346 |
| H | 4.719278 | 3.55151 | 1.263267 | H | -4.77289 | -3.60792 | 1.057505 |
| H | 3.473834 | 2.557734 | 2.028161 | H | -3.58702 | -2.58299 | 1.865935 |
| C | -1.52882 | 0.000009 | -2.51597 | C | 1.538638 | -8E-06 | -2.50573 |
| N | -1.79188 | 0.00001 | -3.65795 | N | 1.79237 | -1.1E-05 | -3.64934 |
| H | 1.116259 | -5.2E-05 | 5.087378 | H | -1.07785 | 0.000019 | 5.090221 |
| F | 6.790283 | 0.000035 | -1.15342 | F | -6.80029 | -4E-06 | -1.01472 |

**Table S11**. Cartesian coordinates for optimized structures of **F-3**.

| Ground state of **F-3** (S_0_) | | | | Excited state of **F-3** (T_1_) | | | |
| --- | --- | --- | --- | --- | --- | --- | --- |
| Ir | 1.024575 | -2E-06 | -0.64341 | Ir | 1.035751 | -3E-06 | -0.63179 |
| N | 0.890645 | 3.219493 | -0.66158 | N | 0.903264 | 3.221808 | -0.66686 |
| N | 2.796017 | 2.299518 | -0.23452 | N | 2.806047 | 2.302052 | -0.21897 |
| N | 2.796049 | -2.29949 | -0.23449 | N | 2.806002 | -2.30209 | -0.21895 |
| N | 0.890693 | -3.2195 | -0.66156 | N | 0.903204 | -3.22181 | -0.66684 |
| C | 1.802611 | 4.248402 | -0.43106 | C | 1.817548 | 4.250459 | -0.44842 |
| H | 1.516583 | 5.286208 | -0.48513 | H | 1.535691 | 5.288268 | -0.52111 |
| N | -2.02378 | -0.00002 | -1.71303 | N | -2.05275 | 0.000013 | -1.6686 |
| N | -1.68887 | -5E-06 | 0.455304 | N | -1.69596 | 0.000016 | 0.506708 |
| N | -4.02419 | -3E-06 | 1.196117 | N | -3.98628 | 0.000015 | 1.32892 |
| C | 3.675745 | 1.19853 | -0.04638 | C | 3.685766 | 1.199529 | -0.02789 |
| C | -0.53859 | 3.407918 | -0.89601 | C | -0.52063 | 3.406859 | -0.94733 |
| H | -0.86985 | 2.57475 | -1.51755 | H | -0.83228 | 2.561134 | -1.56163 |
| H | -0.66609 | 4.328814 | -1.47263 | H | -0.62725 | 4.317536 | -1.54354 |
| C | 1.480552 | 2.009677 | -0.53693 | C | 1.491295 | 2.015388 | -0.52361 |
| C | 3.675761 | -1.19849 | -0.04636 | C | 3.685742 | -1.19958 | -0.02788 |
| C | -1.03135 | -1.2E-05 | -0.76252 | C | -1.0172 | 0.000013 | -0.77291 |
| C | -0.53854 | -3.40795 | -0.896 | C | -0.52069 | -3.40684 | -0.94732 |
| H | -0.66603 | -4.32885 | -1.4726 | H | -0.62732 | -4.31751 | -1.54353 |
| H | -0.86981 | -2.57479 | -1.51755 | H | -0.83233 | -2.56111 | -1.56161 |
| C | 3.002688 | 3.66811 | -0.16777 | C | 3.01547 | 3.67087 | -0.17003 |
| H | 3.964937 | 4.10251 | 0.046091 | H | 3.977458 | 4.106324 | 0.042808 |
| C | -1.83118 | -3.1E-05 | -3.1573 | C | -1.90658 | 0.000016 | -3.11708 |
| H | -2.29466 | 0.890843 | -3.5912 | H | -2.38744 | 0.889409 | -3.53518 |
| H | -2.29465 | -0.89092 | -3.59118 | H | -2.38743 | -0.88938 | -3.53518 |
| H | -0.76505 | -2.7E-05 | -3.37304 | H | -0.84839 | 0.000019 | -3.36888 |
| C | -3.27512 | -1.7E-05 | -1.11699 | C | -3.28579 | 0.000014 | -1.04619 |
| C | -3.06681 | -8E-06 | 0.279697 | C | -3.05997 | 0.000015 | 0.351208 |
| C | 1.802673 | -4.24839 | -0.43103 | C | 1.81747 | -4.25048 | -0.4484 |
| H | 1.516661 | -5.2862 | -0.4851 | H | 1.535595 | -5.28829 | -0.52108 |
| C | 2.998498 | 0.000014 | -0.22496 | C | 3.012407 | -2.1E-05 | -0.2118 |
| C | -2.84098 | -3.54492 | 0.171348 | C | -2.85861 | -3.56555 | 0.027078 |
| H | -3.15383 | -2.71693 | -0.47734 | H | -3.1461 | -2.73104 | -0.62419 |
| H | -3.07353 | -4.46634 | -0.37891 | H | -3.06341 | -4.48319 | -0.54041 |
| C | -0.89403 | 0.000002 | 1.636022 | C | -0.89969 | 0.000016 | 1.639023 |
| C | -1.4484 | 0.000006 | 2.916174 | C | -1.44293 | 0.000021 | 2.940985 |
| H | -2.52232 | 0.000005 | 3.050968 | H | -2.51782 | 0.000024 | 3.06828 |
| C | -0.58768 | 0.000011 | 4.015533 | C | -0.57454 | 0.00002 | 4.016959 |
| C | 0.781505 | 0.000012 | 3.780443 | C | 0.805788 | 0.000015 | 3.764159 |
| C | 1.328933 | 0.000008 | 2.501048 | C | 1.347796 | 0.000009 | 2.485716 |
| H | 2.408355 | 0.000009 | 2.392687 | H | 2.426015 | 0.000005 | 2.372778 |
| C | 0.493569 | 0.000003 | 1.378824 | C | 0.506933 | 0.000008 | 1.368402 |
| C | 1.48058 | -2.00967 | -0.53691 | C | 1.491256 | -2.0154 | -0.5236 |
| C | -5.28062 | -8E-06 | 0.719808 | C | -5.27258 | 0.000015 | 0.836603 |
| H | -6.09364 | -4E-06 | 1.438697 | H | -6.0856 | 0.000016 | 1.553366 |
| C | 3.00274 | -3.66808 | -0.16773 | C | 3.0154 | -3.67091 | -0.17001 |
| H | 3.964995 | -4.10247 | 0.046128 | H | 3.97738 | -4.10638 | 0.042837 |
| C | -1.33146 | -3.45694 | 0.410693 | C | -1.35942 | -3.47855 | 0.328697 |
| H | -1.09525 | -2.56162 | 0.994204 | H | -1.15068 | -2.5901 | 0.932675 |
| H | -0.99594 | -4.31533 | 1.006307 | H | -1.04426 | -4.34382 | 0.925612 |
| C | -1.33151 | 3.45692 | 0.410679 | C | -1.35935 | 3.478568 | 0.328689 |
| H | -1.0953 | 2.561594 | 0.994188 | H | -1.15061 | 2.590109 | 0.93266 |
| H | -0.99597 | 4.315297 | 1.006294 | H | -1.04418 | 4.34383 | 0.925606 |
| C | -2.84103 | 3.544916 | 0.171338 | C | -2.85855 | 3.565579 | 0.027078 |
| H | -3.15388 | 2.716919 | -0.47735 | H | -3.14605 | 2.731072 | -0.62419 |
| H | -3.07357 | 4.466332 | -0.37892 | H | -3.06334 | 4.483216 | -0.54041 |
| C | 5.686686 | 0.000036 | 0.459871 | C | 5.693158 | -4.4E-05 | 0.489381 |
| H | 6.738895 | 0.000045 | 0.724807 | H | 6.743706 | -5.4E-05 | 0.760419 |
| C | 5.027302 | -1.22765 | 0.29635 | C | 5.035253 | -1.22827 | 0.323206 |
| H | 5.566293 | -2.15893 | 0.438753 | H | 5.573628 | -2.15899 | 0.470881 |
| C | -4.5681 | -2.2E-05 | -1.61957 | C | -4.60508 | 0.000015 | -1.55865 |
| H | -4.80702 | -2.9E-05 | -2.67608 | H | -4.87007 | 0.000009 | -2.60476 |
| C | 5.027285 | 1.227714 | 0.296336 | C | 5.035278 | 1.22819 | 0.323195 |
| H | 5.566264 | 2.159004 | 0.438729 | H | 5.573671 | 2.158903 | 0.470862 |
| C | -3.64193 | 3.500115 | 1.474392 | C | -3.71477 | 3.533125 | 1.29486 |
| H | -3.45387 | 2.567544 | 2.014922 | H | -3.57145 | 2.593337 | 1.836709 |
| H | -4.71752 | 3.561913 | 1.281695 | H | -4.77901 | 3.61167 | 1.052625 |
| H | -3.36902 | 4.332472 | 2.132291 | H | -3.45897 | 4.361067 | 1.96572 |
| C | -5.56786 | -1.7E-05 | -0.64681 | C | -5.56439 | 0.000015 | -0.52562 |
| C | -3.64189 | -3.50011 | 1.474399 | C | -3.71484 | -3.53309 | 1.294856 |
| H | -3.36899 | -4.33247 | 2.132301 | H | -3.45905 | -4.36104 | 1.965715 |
| H | -4.71748 | -3.5619 | 1.281699 | H | -4.77908 | -3.61162 | 1.052615 |
| H | -3.45382 | -2.56754 | 2.014927 | H | -3.57151 | -2.5933 | 1.836706 |
| C | 1.416885 | -8E-06 | -2.65927 | C | 1.422437 | -1.6E-05 | -2.64704 |
| N | 1.644651 | -1.1E-05 | -3.80866 | N | 1.638276 | -2.3E-05 | -3.79824 |
| H | -0.96545 | 0.000015 | 5.031418 | H | -0.93192 | 0.000024 | 5.04004 |
| F | -6.85781 | -2.1E-05 | -1.0357 | F | -6.86396 | 0.000014 | -0.88127 |
| F | 1.620817 | 0.000017 | 4.848012 | F | 1.638012 | 0.000015 | 4.824193 |

**Table S12**. The charge character of the lowest triplet excited states (T_1_) of the complexes optimized by TD-B3LYP using the IFCT (Mulliken-like) method.

| **Complex** | **CT (Charge Transfer)** | | | **LE (Local excitation)** |
| --- | --- | --- | --- | --- |
|  | **^3^MLCT(%)** | **^3^LLCT(%)** | **SUM(%)** | **SUM(%)** |
| **CF_3_-1** | 32.7 | 61.8 | 94.5 | 5.5 |
| **CF_3_-2** | 32.3 | 62.3 | 94.6 | 5.4 |
| **CF_3_-3** | 31.3 | 63.7 | 95.0 | 5.0 |
| **F-1** | 33.1 | 14.3 | 47.4 | 52.6 |
| **F-2** | 31.8 | 15.9 | 47.7 | 52.3 |
| **F-3** | 29.2 | 13.7 | 42.9 | 57.1 |

Electroluminescence


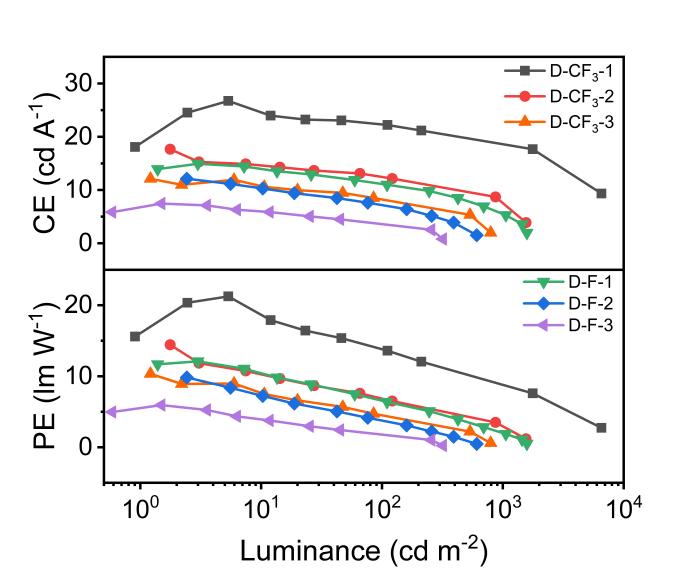


**Figure S73.** Luminance-current efficiency (CE) /power efficiency (PE) characteristics of devices **D-CF_3_-1/2/3** and **D-F-1/2/3**

**Table S13**. External quantum efficiency (EQE)-CIEy relationship of recently reported deep-blue Ph-OLED devices

| Complex | V_on_ (eV) | EQE_max_ | λ_EL_ (nm) | CIE (x,y) | citation |
| --- | --- | --- | --- | --- | --- |
| (TF)_2_Ir(fptz) | 3.3 | 8.4 | 448 | (0.15, 0.12) | ^1^ |
| (HF)_2_Ir(fptz) | 3.4 | 8.4 | 448 | (0.15, 0.13) | ^1^ |
| *fac*-Ir(pmp)_3_ | >7 | 10.1 | 418 | (0.16,0.09) | ^2^ |
| *mer*-Ir(pmp)_3_ | >7 | 14.4 | 465 | (0.16,0.15) | ^2^ |
| Ir1 | 3.5 | 11.2 | - | (0.15,0.13) | ^3^ |
| Ir2 | 3.5 | 13.0 | - | (0.14,0.11) | ^3^ |
| *m*-Ir-(dbfmi)_3_ | 2.5 | 18.2 | - | (0.14, 0.14) | ^4^ |
| Ir(dpt)_3_ | 3.9 | 22.5 | - | (0.15, 0.11) | ^5^ |
| Ir3 | - | 16.9 | - | (0.14,0.15) | ^6^ |
| *mer*-Ir(tfpmi)_3_ | 4.05 | 13.4 | 481 | (0.15,0.05) | ^7^ |
| *mer*-Ir(CF_3_pbp)_3_ | 5.8 | 21.2 | 420 | (0.16,0.05) | ^8^ |
| Ir(cb)_3_ | - | 27.6 | 456 | (0.12,0.13) | ^9^ |
| m-tz1 | 5.3 | 10.0 | 432,444 | (0.15,0.06) | ^10^ |
| m-tz2 | 4.1 | 7.0 | 432,444 | (0.15,0.07) | ^10^ |
| f-ct6a | 3.5 | 18.9 | 460 | (0.15,0.12) | ^11^ |
| f-ct6c | 3.4 | 20.1 | 460 | (0.14,0.13) | ^11^ |

**
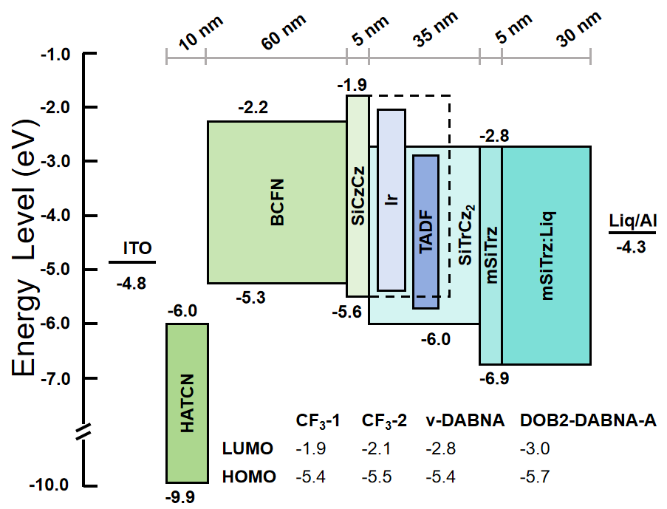
**

**Figure S74.** Device structure of Hyper-OLED and the corresponding energy levels of each layer

**
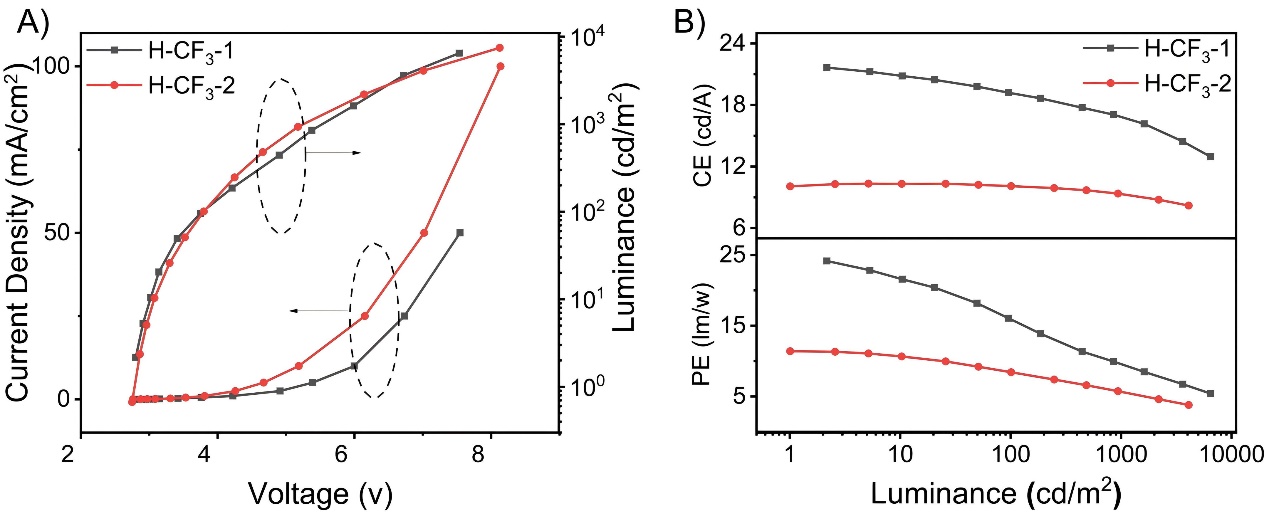
**

**Figure S75. A)** Current density-voltage-luminance (J-V-L) characteristics and B) Luminance-current efficiency (CE) /power efficiency (PE) characteristics of device **H-CF_3_-1 and H-CF_3_-2**


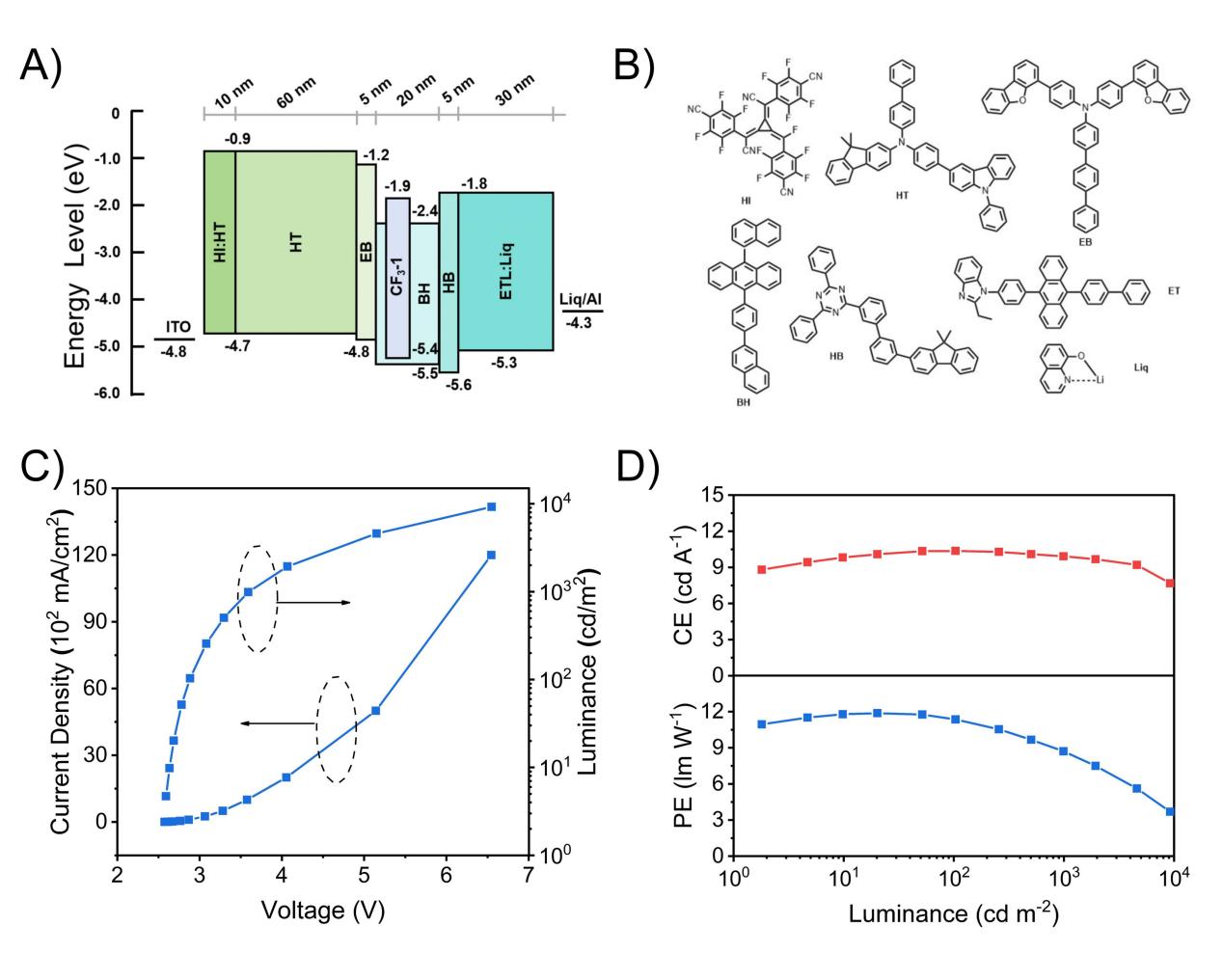


**Figure S76.** (a) Device structure of Ph-OLED and the corresponding energy levels of each layer, (b) Molecular involved in this Ph-OLED, (c) Current Density-Voltage-Luminance curve, (d) Current efficiency/Power efficiency-Lumiance curve

Reference

1. Lee, S. et al. Deep-Blue Phosphorescence from Perfluoro Carbonyl-Substituted Iridium Complexes. *Journal of the American Chemical Society* **38,** 14321 (2013).

2. Lee, J. et al. Thompson, M. E.; Forrest, S. R., Deep blue phosphorescent organic light-emitting diodes with very high brightness and efficiency. *Nature Materials* **15,** 92 (2016).

3. Feng, Y. et al. Rational design and characterization of heteroleptic phosphorescent iridium(iii) complexes for highly efficient deep-blue OLEDs. *Journal of Materials Chemistry C* **4,** 10246-10252 (2016).

4. Cho, Y.-J. et al. Probing photophysical properties of isomeric N-heterocyclic carbene Ir(iii) complexes and their applications to deep-blue phosphorescent organic light-emitting diodes. *Journal of Materials Chemistry C* **5,** 1651-1659 (2017).

5. Li, X. et al. Deep Blue Phosphorescent Organic Light-Emitting Diodes with CIEy Value of 0.11 and External Quantum Efficiency up to 22.5%. *Advanced Materials* **30**, 1705005 (2018).

6. Kim, S. et al. Degradation of blue-phosphorescent organic light-emitting devices involves exciton-induced generation of polaron pair within emitting layers. *Nature Communications* **9**, 1211 (2018).

7. Pal, A. K. et al. High-Efficiency Deep-Blue-Emitting Organic Light-Emitting Diodes Based on Iridium(III) Carbene Complexes. *Advanced Materials* **30,** 1804231 (2018).

8. Kumaresan, R. et al. High Performance Solution-Processed Deep-Blue Phosphorescence Organic Light-Emitting Diodes with EQE Over 24% by Employing New Carbenic Ir(III) Complexes. *Advanced Optical Materials* **10**, 2101686 (2022).

9. Jung, M. et al. A bipolar host based high triplet energy electroplex for an over 10 000 h lifetime in pure blue phosphorescent organic light-emitting diodes. *Materials Horizons* **7**, 559 (2020).

10. You, C. et al. Homoleptic Ir(III) Phosphors with 2-Phenyl-1,2,4-triazol-3-ylidene Chelates for Efficient Blue Organic Light-Emitting Diodes. *ACS Applied Materials & Interfaces* **13**, 59023 (2021).

11. Yan, J. et al. Blue Electrophosphorescence from Iridium(III) Phosphors Bearing Asymmetric Di-N-aryl 6-(trifluoromethyl)-2H-imidazo[4,5-b]pyridin-2-ylidene Chelates. *Advanced Materials* **10**, 2305273 (2023).

12. Mackenzie, C. F. R. et al. Bulky Iridium NHC Complexes for Bright, Efficient Deep-Blue OLEDs. *Advanced Optical Materials* **11**, 2201495 (2023).
